# Neural bases of self‐ and object‐motion in a naturalistic vision

**DOI:** 10.1002/hbm.24862

**Published:** 2019-11-11

**Authors:** Sabrina Pitzalis, Chiara Serra, Valentina Sulpizio, Giorgia Committeri, Francesco de Pasquale, Patrizia Fattori, Claudio Galletti, Rosamaria Sepe, Gaspare Galati

**Affiliations:** ^1^ Department of Movement, Human and Health Sciences University of Rome Foro Italico Rome Italy; ^2^ Cognitive and Motor Rehabilitation Unit Santa Lucia Foundation (IRCCS Fondazione Santa Lucia) Rome Italy; ^3^ Department of Biomedical and Neuromotor Sciences University of Bologna Bologna Italy; ^4^ Laboratory of Neuropsychology and Cognitive Neuroscience, Department of Neuroscience Imaging and Clinical Sciences, and Institute for Advanced Biomedical Technologies (ITAB), University G. d'Annunzio Chieti Italy; ^5^ Faculty of Veterinary Medicine University of Teramo Teramo Italy; ^6^ Department of Pharmacy and Biotechnology University of Bologna Bologna Italy; ^7^ Brain Imaging Laboratory, Department of Psychology Sapienza University Rome Italy

**Keywords:** area V6, brain mapping, flow parsing, fMRI, optic flow, wide‐field

## Abstract

To plan movements toward objects our brain must recognize whether retinal displacement is due to self‐motion and/or to object‐motion. Here, we aimed to test whether motion areas are able to segregate these types of motion. We combined an event‐related functional magnetic resonance imaging experiment, brain mapping techniques, and wide‐field stimulation to study the responsivity of motion‐sensitive areas to pure and combined self‐ and object‐motion conditions during virtual movies of a train running within a realistic landscape. We observed a selective response in MT to the pure object‐motion condition, and in medial (PEc, pCi, CSv, and CMA) and lateral (PIC and LOR) areas to the pure self‐motion condition. Some other regions (like V6) responded more to complex visual stimulation where both object‐ and self‐motion were present. Among all, we found that some motion regions (V3A, LOR, MT, V6, and IPSmot) could extract object‐motion information from the overall motion, recognizing the real movement of the train even when the images remain still (on the screen), or moved, because of self‐movements. We propose that these motion areas might be good candidates for the “flow parsing mechanism,” that is the capability to extract object‐motion information from retinal motion signals by subtracting out the optic flow components.

## 1 INTRODUCTION

Detection of moving objects or living entities in our surroundings is among the most fundamental abilities of the visual system. Cells specialized in the detection of movement within their receptive fields exist in multiple brain regions at all levels of the visual processing hierarchy. However, a shift of the retinal image of an object may occur not only when the object moves, but also when we move within an otherwise static environment, or when we move our eyes or head. On the other side, in some cases the retinal image of a moving object may not move at all, for example, when we follow a moving object with our gaze, maintaining its image still on the fovea (smooth pursuit). Thus, our motion perception consists of much more than the detection of retinal shifts and involves recognizing whether retinal shifts are due to true object displacements or generated by our own movements, or by some combination of the two. This recognition mechanism has been called flow parsing (Warren & Rushton, 2009a, 2009b) and would consist in the segregation of retinal motion information into an object‐motion and an ego component. The self‐motion (egomotion) component would be estimated based on an analysis of the optic flow (as well as of vestibular, proprioceptive, and other nonvisual sources of information), that is, the visual stimulation generated on the retina by the observer's movement (Koenderink & Physics, 1986); then, self‐motion would be “subtracted” from retinal motion to compute an estimation of “real” object‐motion.

Neural representation of optic flow has been extensively studied in macaques and humans. In monkeys, optic flow activates several higher‐level motion areas, including the middle superior temporal area (MST; Duffy, 1998), the ventral intraparietal area (VIP; Duhamel, Colby, & Goldberg, 1998; Bremmer et al., 2001) and the caudal area PE (PEc) in the posterior parietal cortex (Raffi, Squatrito, & Maioli, 2002). Human neuroimaging studies have found specificity for optic flow in a larger cortical network of temporal, parietal, insular, and cingulate regions, including MST (or MT+; Tootell et al., 1995; Morrone et al., 2000; Pitzalis, Sdoia, et al., 2013), VIP (Bremmer et al., 2001; Cardin & Smith, 2010; Sereno & Huang, 2006), V6 (Pitzalis et al., 2010; Serra et al., 2019), PCi and CSv (Cardin & Smith, 2010), the parieto‐insular vestibular cortex (PIVC or PIC; Cardin & Smith, 2010), and the newly defined human homolog of macaque PEc in the anterior precuneus (Pitzalis et al.,2019).

Regarding object‐motion, several cortical areas of the dorsal visual stream in the monkey contain many “real motion cells” (for a review, see Galletti & Fattori, 2003), that is, cells activated by the actual movement of an object in the visual field, but not, or not so strongly, by an identical shift of the object retinal image produced by an eye movement. These cells were found in V1 (Bridgeman, 1973; Galletti, Squatrito, Battaglini, & Grazia Maioli, 1984), V2 (Galletti, Battaglini, & Aicardi, 1988), V3A (Galletti, Battaglini, & Fattori, 1990), V6 (Galletti & Fattori, 2003), MT/V5 (Erickson & Thier, 1991), MST (Erickson & Thier, 1991), and 7a (Sakata, Shibutan, Kawano, & Harrington, 1985). Likely, this type of cells is involved in object‐motion detection in the visual field (Galletti & Fattori, 2003), even in such critical situations as when the retinal images are continuously in motion because of self‐motion (Grossberg, Mingolla, & Pack, 1999). Galletti and Fattori (2003) suggested that the activity of these cells is responsible for the flow‐parsing mechanism and hypothesized that these cells, besides recognizing real motion in the visual field, are the elements of a cortical network that represents an internal map of a stable visual world.

In humans, little is known about the neural basis of the mechanisms allowing to disentangle self‐ and object‐motion from retinal information. Recent functional magnetic resonance imaging (fMRI) data suggest that some dorsal motion areas (like V3A and V6) likely contribute to perceptual stability during pursuit eye movements (Fischer, Bülthoff, Logothetis, & Bartels, 2012). In particular, V6 could be involved in “subtracting out” self‐motion signals across the whole visual field and in providing information about moving objects, as originally suggested by our group (Galletti & Fattori, 2003; Pitzalis et al., 2010, 2015; Pitzalis, Fattori, & Galletti, 2013) and later on by others (Arnoldussen, Goossens, & van den Ber, 2013; Cardin, Sherrington, et al., 2012; Fischer et al., 2012). These suggestions remain however at a speculative level and there is not yet a clear and complete picture of the brain regions involved in flow parsing, as well as it is still unclear whether distinct cortical areas are engaged in the detection of object‐ and self‐motion as previously suggested (Previc, 1998; Previc, Liotti, Blakemore, Beer, & Fox, 2000; Rosa & Tweedale, 2001).

The aim of the present fMRI work is to identify human cortical motion‐sensitive areas responding to object‐ and/or to self‐motion, and whether they are capable to extract object‐motion signals from the overall motion. To this aim, we used an event‐related fMRI experiment, brain mapping methods, and wide‐field stimulation to compare visually induced self‐ and object‐motion in a realistic virtual environment, in conditions of natural vision (free scanning). We also investigated the flow‐parsing phenomenon using a complex motion stimulation combining self‐ and object‐motion, where the object‐motion must be inferred (i.e., extracted) from the overall motion by subtracting out optic flow information. To reproduce a realistic motion stimulation, we used a virtual reality software simulating a train moving in a natural landscape and movies as motion conditions. To minimize the effect of possible physical differences between trials, we estimated the quantity and direction of motion embedded in each single frame through a block‐matching algorithm (BMA; see Bartels, Zeki, & Logothetis, 2008 for a similar approach). The use of wide‐field stimuli (as described in Pitzalis et al., 2010) increased the realism of the virtual environment and made the illusory impression of self‐motion (vection) particularly compelling.

Importantly, we performed preliminary psychophysical experiments to verify and quantify the vection evoked by the different types of visual motion. In addition, we used dedicated functional localizers in each individual subject to map the position of area V6 (Pitzalis et al., 2010; Serra et al., 2019) and to distinguish specific partitions within the MT+ complex, that is, MT/V5 and MST+ (Dukelow et al., 2001; Huk, Dougherty, & Heeger, 2002). This was necessary because while the MT+ complex is universally recognized as motion sensitive, the different role of MT/V5 and MST+ in self‐motion perception is still debated (Huang, Chen, & Sereno, 2015; Kleinschmidt et al., 2002; Pitzalis, Sdoia, et al., 2013; Wall & Smith, 2008). We also mapped the position of scene‐responsive regions (parahippocampal place area [PPA] and retrosplenial complex [RSC]; Epstein & Kanwisher, 1998; Sulpizio, Committeri, Lambrey, Berthoz, & Galati, 2013; Sulpizio, Committeri, & Galati, 2014) to verify their possible response to self‐ or object‐motion, since it has been recently shown that the PPA responds to visual motion in ecological scenes (Korkmaz Hacialihafiz & Bartels, 2015), but its role in self‐motion processing is still unclear.

## 2 METHODS

### 2.1 Participants

Fourteen healthy adults (mean age 24 years, range 20–26, six females) participated to the behavioral study and to two fMRI acquisition sessions in separate days, the first one to perform the main experiment and the second one to perform two localizer scans to map areas V6+ and PPA/RSC. A subgroup of subjects (*n* = 8) underwent a third fMRI acquisition session in a separate day to perform a localizer scan to map areas MT and MST+. All participants had normal or corrected‐to‐normal visual acuity and no history of psychiatric or neurological disease. All participants had extensive experience in psychophysical and fMRI experiments and were paid for their participation. They also gave written informed consent and all procedures were approved by the Ethics Committee of Fondazione Santa Lucia, Rome, Italy.

### 2.2 Train experiment

**Figure 1:**
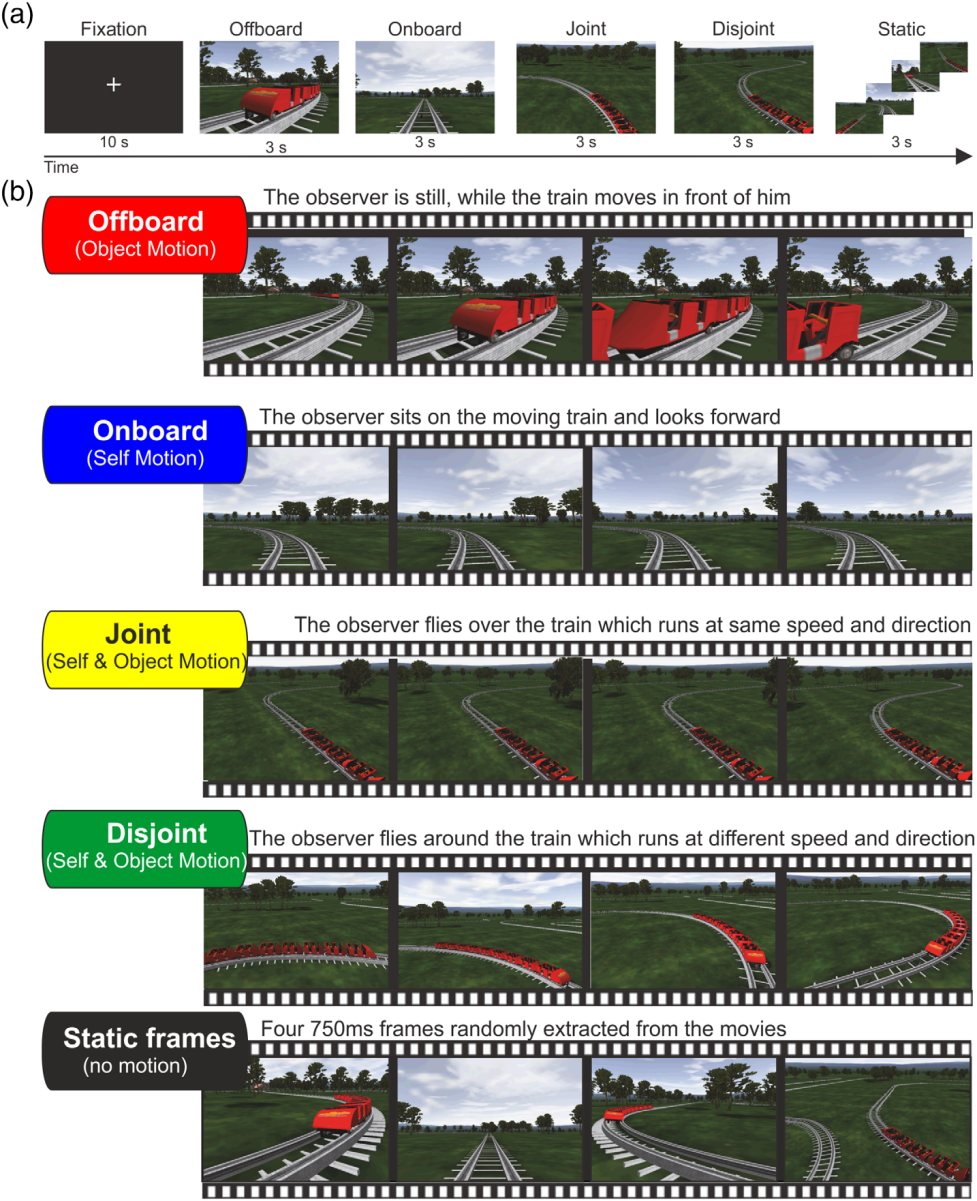
Schematic representation of the stimuli we used in the motion fMRI experiment (train). (a) Summary of trials sequence used in the experiment. (b) Example of the 3 s movie types for the four motion conditions (Offboard‐red, Onboard‐blue, Joint‐yellow, and Disjoint‐green) and static frames (Black). fMRI, functional magnetic resonance imaging

In the main event‐related fMRI experiment, hereafter called train experiment, we presented movies and snapshots from a realistic virtual environment representing different combinations of visually induced self‐ and object‐motion. To reproduce a realistic motion stimulation, we used virtual reality gaming software (NoLimits Roller Coaster Simulation, Mad‐Data GmbH & Co. KG, Erkrath, Germany). The environment included a train moving on a railway (speed: 60 km/hr), alternating straight and curvilinear plain stretches, within a realistic natural landscape rich in details (see Figure 1 and Supporting Information movie files). Each trial consisted in the passive observation of a 3 s movie belonging to either of five different experimental conditions (each of which has been consistently associated for clarity to a specific color in all figures in the article):

1. Offboard condition (red): the subject is still and observes the train moving in front of him (see Movie S1). This visual stimulation is consistent with pure object‐motion in the absence of self‐motion.
2. Onboard condition (blue): the subject is virtually sitting on the moving train and looks forward ahead (see Movie S2). This visual stimulation is consistent with pure self‐motion within a static environment.
3. Joint condition (yellow): the subject virtually runs next to the moving train at a fixed distance (see Movie S3). In this condition, both the subject and the train move together along the same trajectory and at the same speed. Interestingly, although this results in a combination of self‐ and object‐motion, the train has a fixed position on the screen throughout the movie. In this situation, the subject perceives the train, which is fixed on the retina, as moving, and the external environment, which is shifting on the retina, as static.
4. Disjoint condition (green): as above, but the train is not moving along the same trajectory and at the same speed as the subject, thus its image on the screen is not fixed (see Movie S4).
5. Static condition (black): these pseudo‐movies were a sequence of four frames lasting 750 ms each, randomly extracted from four different movies belonging to the four previously described categories, in order not to evoke any motion sensation.

In each condition (Onboard, Joint, Disjoint, and Offboard) we used three different types of movies showing a train moving along a (a) rightward curvilinear trajectory, (b) leftward curvilinear trajectory, and (c) straight trajectory. We ensured that each condition had equally many target‐movie instances to avoid that attentional modulation could bias responses for particular conditions. It is noteworthy to say that we used four different categories of sample movie clips with different perspectives. As shown in Figure 1, Onboard and Offboard conditions are earthbound with a similar horizon, while the more complex motion conditions (Joint and Disjoint) are actually in aerial views (simulating the view of an observer flying over the train). Thus, the low‐level stimulus features in the complex motion conditions cannot be considered a direct summation of those from the pure self‐motion (Onboard) and object‐motion (Offboard). We restricted the duration of each movie to 3 s both to take into account the strong BOLD response adaptation to flow and to minimize the amount of unwanted spontaneous eye movements during the visualization of the visual scene (which is an important methodological point since we placed no constraints on eye movements).

Each participant underwent six consecutive fMRI acquisition scans lasting approximately 5′30″ each. During each scan, we presented a different pseudorandomized sequence (i.e., trial order was different for each scan but fixed across subjects), which included 15 different trials for each of the five conditions, plus 5 target trials (see below), for a total of 80 trials. The trial sequence was balanced such that each trial was preceded with equal frequency by all trial types. Trials were presented every 3 s and the intertrial interval was set to zero so that the movies were displayed one after the other (without any gap between them), to minimize the brisk on–off switch of the movies that could bias the neural response. Every set of 10 trials was interleaved with a 10 s fixation period. The fixation period (white central cross on a black background) constituted the low‐level baseline for the study. This procedure yielded 80 trials and 8 fixation periods per subject for each scan.

In order to obtain a visual experience as natural as possible, we did not constrain subjects to maintain fixation while viewing movies. To keep their attention high during the experiment, subjects were engaged in a one‐back task. Subjects pressed a button with the right index finger each time the very same movie (not an instance of the same category) was repeated twice in a row (target trials). Target trials were generated by duplicating five randomly selected trials in each scan. Thus, they occurred randomly and quite rarely, approximately once per minute.

### 2.3 Objective motion measure (BMA)

Movies constitute an excellent experimental approximation to our real‐life visual input and have a great ecological validity. However, they are uncontrolled visual stimuli and need further analysis to control for possible physical differences between stimuli. To summarize the properties of motion in each movie, we estimated a set of parameters derived from the amplitude and the direction of a set of motion vectors estimated from the movie frames.

**Figure 2:**
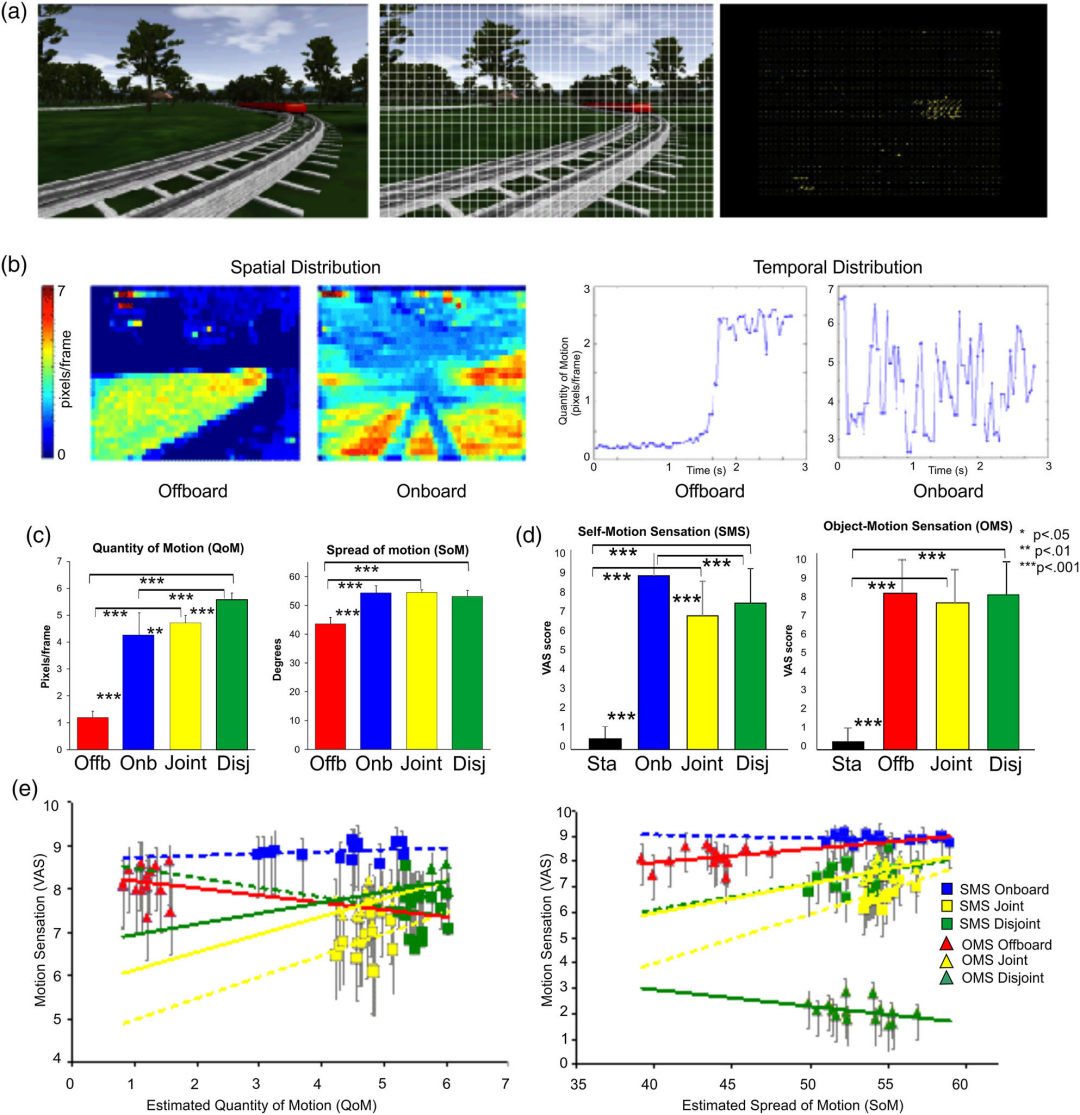
Motion estimation procedures in the train experiment. (a) Block‐matching design Algorithm (BMA). Example of estimation procedure for an Offboard movie. Movie frames (left panel) are tessellated through a set of macro‐blocks (central panel‐yellow grid). The resulting motion vector field obtained by BMA is shown on the right panel. (b) Spatial (left) and temporal distribution (right) of the estimated quantity of motion for two representative movies (Offboard and Onboard). (c) Quantity of motion (QoM) and spread of motion (SoM) for the four movie types and static condition averaged across trials. (d) Psychophysical results. Histograms show the intensity of self‐motion sensation (SMS) and object‐motion sensation (OMS) revealed by VAS scale across subjects. Bars represent the mean VAS scores ± *SE* of the mean across runs and participants. In (c) and (d) name abbreviations for some of the conditions are as follows: Sta (Static), Offb (Offboard), Onb (Onboard), Disj (Disjoint). (e) Scatterplots of SMS and OMS versus QoM and SoM in the Offboard (Red), Onboard (Blue), Joint (Yellow), and Disjoint (Green) conditions. QoM, quantity of motion; SoM, spread of motion; VAS, visual‐analog scale

To this aim, we developed a BMA. In the literature, BMA is a well‐established class of algorithms for object tracking, video encoding, and compression (Bashkaran & Konstantinides, 1995; Chen, 2009; Chen, Hung, & Fuh, 2001; Gallant, Cote, & Kossentini, 1999; Hui & Siu, 2007; Jain & Jain, 1981; Moshe & Hel‐Or, 2009; Nie & Ma, 2002). The basic assumption is that the spatial patterns corresponding to objects and background in a frame of a video sequence move to form corresponding objects in the subsequent frame. To estimate this movement, BMA tessellates every movie frame into a set of macroblocks (see Figure 2a). Then, the movement of each macroblock at the current frame is estimated by comparing the macroblock with its neighbors in the previous frame. This is achieved by minimizing a cost function. The movement is represented by a motion vector whose 2D components are stored so that the amplitude and the direction of the motion can be recovered for each macroblock (see Figure 2a). This step is repeated at each macroblock and for every pair of frames comprising the movie under investigation to provide a sequence of vectors that allows estimating the evolution of motion through time. It is thus possible to have a spatial and temporal distribution of the quantity of motion (QoM) present in each 3 s movie (see Figure 2b).

Important parameters must be set to obtain reliable estimates of movement such as: the macroblock size, the search area, the strategy adopted to explore the neighborhood and the cost function used to match macroblocks across frames. These parameters were set by starting from typical values reported in the literature (Nie & Ma, 2002), that were manually optimized on a set of test frames. Since each frame was composed by 640 by 480 pixels, squares of side 16 pixels were used as macroblocks so that every image was tessellated into 1,350 tiles. The search area was constrained up to *p* pixels on all four sides of the corresponding macroblock. Here, we set *p* = 7 since this corresponds to a maximum shift of 10 cm. This is the maximum movement expected between two successive frames based on the simulated speed of the train.

The block‐matching step in which the “new” location of a given block is estimated was performed by minimizing a cost function depending on the values of the pixels within the considered block. Here, we adopted the mean absolute difference (MAD) given by:

$$\mathrm{MAD}=\frac{1}{N^{2}}\sum_{i=0}^{N-1}\sum_{j=0}^{N-1}\left|C_{\mathrm{ij}}-R_{\mathrm{ij}}\right|$$

in which *N* is the side of the macroblock, and *C*_*ij*_ and *R*_*ij*_ are the pixels being compared in the current and the reference macroblock, respectively. The adoption of MAD is a typical choice in the literature (see, e.g., Chen et al., 2001). The noise in the data may lead to an estimate of movement between still macroblocks in adjacent frames. In order to minimize this effect, we did not include the contribution in MAD of those pixels whose motion did not exceed a threshold called the zero motion threshold (Nie & Ma, 2002). This was estimated by selecting a region of interest corresponding to still objects, for example, trees. This has the advantage to reduce the computational burden of this approach and to make the estimate more robust against noise.

In addition, to reduce the computational time, we implemented a two‐step strategy, the adaptive rood pattern search (ARPS) followed by the diamond search pattern (DSP). ARPS exploits the assumption that typically the motion in a frame is coherent, that is, neighboring macroblocks very likely share similar motion. Thus, for each macroblock its first‐order neighbor on the left predicts its own motion. Once the search is directed to an area of high probability of matching the block, the strategy is changed to DSP in which a DSP is adopted (for details see Nie & Ma, 2002).

We computed two parameters for each movie to characterize the estimated motion: the QoM and the spread of motion (SoM). QoM was defined as the average, across pixels and frames, of the amplitude of the motion vectors estimated in each pixel. SoM was the *SD* of the distribution of orientation values across pixels, averaged across frames. QoM is estimated at each pixel separately and then the mean value is computed within each frame so that the variation of the motion amplitude can be represented over time. SoM is instead a measure of the overall spread of the orientation of the motion in each movie, obtained by estimating the *SD* of the orientation values obtained from all the pixels in all frames of the movie.

Figure 2c shows values, averaged across trials, for QoM (left side) and SoM (right side) in the four motion conditions (Offboard, Onboard, Joint, and Disjoint). The values underwent a separate one‐way ANOVA for QoM and SoM with motion conditions as factor. The analyses were significant for both QoM (*F*[3, 42] = 2,890.1, *p* < .001) and SoM (*F*[3, 42] = 107.6, *p* < .001). A post‐hoc analysis (Duncan's test) on the main effect revealed that both QoM (*p* < .0001) and SoM (*p* < .0001) were significantly lower in the Offboard condition than in the other three conditions. Moreover, QoM was higher in the Disjoint than in the Joint condition (*p* < .0001), and in the Joint than in the Onboard condition (*p* < .01). Overall, as shown in Figure 2c, the QoM reached the higher value in the Disjoint condition, while the Offboard condition was the condition with less QoM and SoM. To correct for the different sensorial contributions between trials and between conditions on the brain activations, we included QoM and SoM in the fMRI analysis as parametric modulators (see 2.8 Statistical analyses of fMRI data).

### 2.4 Subjective motion measure (psychophysical experiment)

While the algorithm described above objectively estimated the QoM present in the different movies, we also performed a psychophysical experiment to quantify the perception of motion experienced by our subjects. This was done in a preliminary behavioral training session, where visual stimuli were presented on a 17′ computer display that subtended the same degrees of visual angle as in the fMRI scanner (70° × 55°, see Section 2.6). Subjects were seated in front of the display in complete darkness, with the head mechanically stabilized with a chin rest and a head holder. Subjects viewed the same stimuli as in the main experiment in a randomized sequence, and answered to the following question immediately after viewing each movie: (a) “How intense was your sensation that you were moving in the space?” (self‐motion sensation [SMS]; for Onboard movies); and (b) “How intense was your sensation that the train was moving?” (object‐motion sensation [OMS]; for Offboard movies). Both questions were also asked for Joint and Disjoint movies and, as a control, for static frames. Participants indicated the intensity of SMS and OMS through a visual‐analog scale (VAS). The VAS was shown on the screen as a 10 cm white horizontal line on a dark background intersected by a small vertical mark. Subjects made the mark slide along the horizontal line by using the computer mouse and clicked when the mark was located at the point they felt to correspond with the subjective intensity of their sensations. The left and right ends of the horizontal line represented no sensation at all and maximal sensation, respectively. The VAS score was determined as the distance (in cm) of the mark from the left end of the line, and thus ranged across a continuum from none (0/10) to a maximum amount of motion sensation (10/10).

### 2.5 Localizer scans

**Figure 3:**
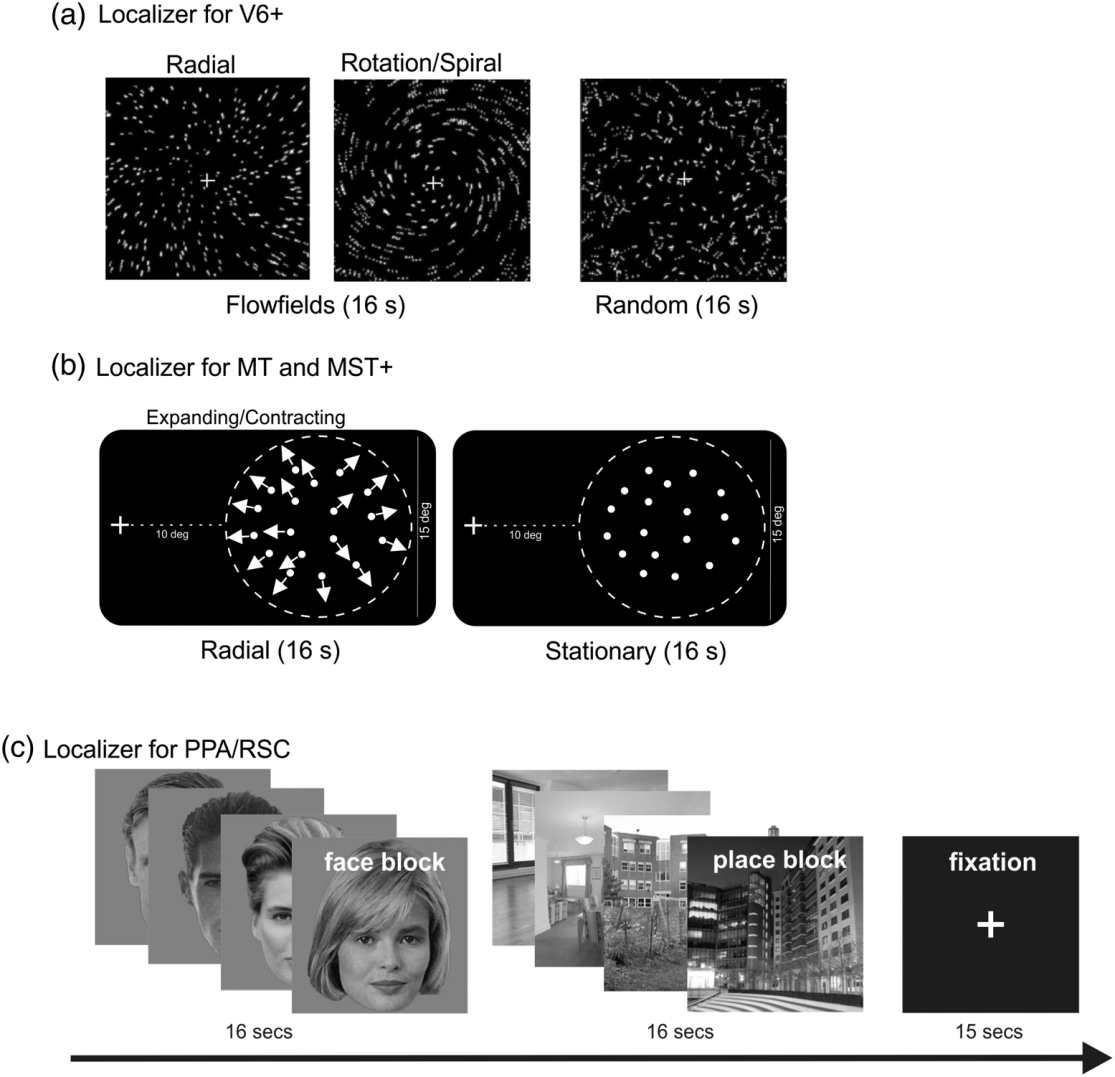
Schematic representation of the stimuli we used for the Localizer scans. (a) Localizer for V6 (flow fields). In this visual motion paradigm, 16‐s blocks of coherently moving fields (flow fields) were interleaved with 16‐s blocks of randomly moving fields. The first two frames phase show the two different types of coherent motion (radial and rotation‐spiral motion) that switched almost every 500 ms during the coherent motion blocks. For both radial and spiral motions, we tested both expansion and contraction components. Modified from Pitzalis et al. (2010). (b) Localizer for MT and MST+ (ipsilateral stimulation). Responses to ipsilateral stimulation were assessed by presenting a peripheral dot patch in either the left or right visual field. The 15° diameter field of dots alternated between moving (16 s) and stationary (16 s), while subjects maintained fixation on a small, high‐contrast white cross 10° from the nearest edge of the dot patch. Modified from Huk et al. (2002). (c) Localizer for PPA/RSC (Face/Place). In this visual paradigm, 16‐s blocks of pictures of human faces (male and female) were interleaved with 16‐s blocks of pictures of places (indoor and outdoor) and with 15‐s block of fixation. Modified from Sulpizio et al. (2013, 2014). PPA, parahippocampal place area; RSC, retrosplenial complex

In a second set of fMRI experiments, several localizer scans were conducted to define V6+, MT/MST+, and PPA/RSC regions of interest (ROIs). V6+ ROI was defined by the flow field stimulus (see Figure 3a) based on the method used in Pitzalis et al. (2010). MT and MST+ were defined by the use of an ipsilateral stimulus (see Figure 3b) based on the method used in Huk et al. (2002). PPA and RSC were defined by contrasting pictures of places/scenes versus faces (see Figure 3c) as described in Epstein (2008). Further details are provided in Supporting Information.

### 2.6 Apparatus

Visual stimuli were generated by control computers (a standard PC and an SGI O2, both equipped with a standard 3D graphics card) located outside the MR room. For the train experiment and the V6+ and PPA/RSC localizer scans, stimuli were presented with an in‐house software, implemented in MATLAB (The MathWorks Inc., Natick, MA) using Cogent 2000 (developed at FIL and ICN, UCL, London, UK) and Cogent Graphics (developed by John Romaya at the LON, Wellcome Department of Imaging Neuroscience, UCL, London, UK). For the MT‐MST+ localizer scan, stimuli were presented with Presentation 9.9 (Neurobehavioral System Inc., Albany, Canada) code by A. Bultrini. These software packages allowed time‐locked presentation of visual stimuli, while maintaining millisecond timing accuracy over a period of minutes and triggering the acquisition of functional MR images. An LCD video projector (Sharp GX‐3800, 640 × 480 pixels, 60 Hz refresh) with a customized lens projected the visual stimuli onto a back‐projection screen attached to the back of the head coil.

For the train experiment and V6+ localizer scans we used a wide‐field setup similar to that originally described by our group (Pitzalis et al., 2006) and then routinely used in many fMRI experiment from our and other laboratories (Cardin & Smith, 2010; Huang et al., 2015; Huang & Sereno, 2013; Pitzalis et al., 2010; Pitzalis, Bozzacchi, et al., 2013; Pitzalis, Sdoia, et al., 2013; Pitzalis, Sereno, et al., 2013; Serra et al., 2019; Strappini et al., 2015, 2017). Visual stimuli were seen in binocular view via a mirror and subtended 70° (±35°) horizontally, 55° (±27.5°) vertically, and 82° (±41°) in an oblique direction. In addition to better reveal areas that emphasize the periphery, the wide‐field stimulation is particularly indicated in those studies (as the present one) where one wants to evoke in the observer a vection sensation, that is the illusory impression of self‐motion induced by virtually manipulating the optic flow changes on the retina (Palmisano, Allison, Schira, & Barry, 2015). Using a wide‐field stimulation, the subject felt to be immersed in the virtual reality environment and induced vection was particularly compelling as revealed by the psychophysical experiment (see behavioral results). For the MT/MST+ and PPA/RSC localizers, we used a standard setup where the average viewing distance was 66.5 cm, subtending a visual screen of 23° × 12°.

In the train experiment, we used free viewing while in all the other sessions subjects were required to gaze at a central cross throughout the period of scan acquisition. The wide‐field visual projection setup did not allow for eye tracking. However, to promote stable fixation (requested during the three functional localizer scans), the fixation point was continuously visible at a fixed position on the screen and only expert subjects with a good fixation stability were used. In the train experiment where subjects were asked to perform a task, responses were given through MR‐compatible push buttons. In all experiments, fixation distance and head alignment were held constant by a chin rest mounted inside the head coil. Subjects' heads were stabilized with foam padding to minimize movement during the scans.

### 2.7 Image acquisition and preprocessing

The MR examinations were conducted at the Santa Lucia Foundation (Rome, Italy) on a 3T Siemens Allegra MR system (Siemens Medical Systems, Erlangen, Germany) using a standard head coil. Functional T2*‐weighted images were collected using a gradient echo EPI sequence using blood‐oxygenation level‐dependent imaging (Kwong et al., 1992). For the train experiment and V6+ and MT/MST+ localizers we acquired 30 slices (no gap, interleaved excitation order) oriented approximately parallel to the calcarine sulcus (voxel resolution 3 × 3 × 4 mm; repetition time [TR] 2 s; echo time [TE] 30 ms, flip angle 70°; bandwidth 2,298 Hz/pixel; field of view [FOV] 192 × 192 mm). From the superior convexity, sampling included almost all the cerebral cortex, excluding only the ventral portion of the cerebellum. For the PPA/RSC localizer, MR slices were 3 × 3 × 2.5 mm oriented approximately perpendicular to the calcarine sulcus covering only the posterior part of the brain. The number of volumes per acquisition scan was 160 for the train experiment, 128 for the V6+ and MT/MST+ localizer scans, and 242 for the PPA/RSC localizer scan. In each scan, the first four volumes were discarded from data analysis to achieve a steady state, and the stimuli started at the beginning of the fifth volume. Each participant underwent six consecutive fMRI scans for the train experiment and four scans for the localizers (two for V6+ and two for PPA/RSC). Eight participants underwent also two scans for the MT/MST+ localizer. Overall, a total of 156 scans were carried out on the 14 subjects (84 scans for the train experiment and 72 scans for the functional localizer). Structural images were collected using a sagittal magnetization‐prepared rapid acquisition gradient echo (MPRAGE) T1‐weighted sequence (TR = 2,000 ms, TE = 4.38 ms, TI = 910 ms, flip angle = 8°, bandwidth = 130 Hz/pixel, 2563 × 256 image matrix, 1 mm^3^ voxels, 176 contiguous slices).

Structural images were analyzed using FreeSurfer 5.1 (http://surfer.nmr.mgh.harvard.edu/) to obtain a surface representation of each individual cortical hemisphere in a standard space based on standard procedures described in Supporting Information.

Functional images were realigned within and across scans to correct for head movement and coregistered with structural MPRAGE scans using SPM12 (Wellcome Department of Cognitive Neurology, London, UK). Images were then spatially normalized using an automatic nonlinear stereotaxic normalization procedure (final voxel size: 3 × 3 × 3 mm) and spatially smoothed with a three‐dimensional Gaussian filter (6 mm full‐width‐half‐maximum). The template image for spatial normalization was based on average data provided by the Montreal Neurological Institute (Mazziotta, Toga, Evans, Fox, & Lancaster, 1995) and conforms to a standard coordinate referencing system (Talairach & Tournoux, 1988).

### 2.8 Statistical analyses of fMRI data

#### 2.8.1 Train experiment

The time series of functional MR images was first analyzed separately for each participant. The effects of the experimental paradigm were estimated on a voxel‐by‐voxel basis, according to the general linear model. For the train experiment, the onset of each trial constituted a neural event, which was modeled through a canonical hemodynamic response function, chosen to represent the relationship between neuronal activation and blood flow changes. Separate regressors were included for each trial type (Offboard, Onboard, Joint, Disjoint, and Static), yielding parameter estimates for the average hemodynamic response evoked by each trial type. We did not explicitly modeled blocks of fixation motion as GLM regressors that were rather treated as part of the residual variance.

Further regressors were added as parametric modulators, which modeled the (linear) contribution of the estimated motion parameters (QoM and SoM) and of the subjective motion sensation intensities (SMS and OMS) on the neural response of each specific trial. Thus, the response to each trial was modeled as a linear combination of the average response to that trial type, and of the effect of QoM, SoM, SMS, and OMS. Motion parameters (QoM and SoM) were modeled independently of the specific trial type, while subjective motion sensation intensities (SMS and OMS) were allowed to have a differential impact on each trial type. In other words, an additional regressor (“movement”) was added to control for the presence of any type of objective motion, independently of the specific motion condition and then used to test the effect of QoM and SoM (see below). On the other side, SMS and OMS were differently used as parametric modulators in specific trial types. Both SMS and OMS were used as parametric modulators in both Joint and Disjoint conditions. However, since SMS and OMS scores are absent, respectively, for the Offboard and Onboard trials, because these two conditions only include object‐ and self‐motion, respectively (see Section 3), SMS (but not OMS) was used to model the Onboard condition, and OMS (but not SMS) was used to model the Offboard condition. No parametric modulators were used for the Static condition. Thus, the resulting model included a total of 15 regressors for each scan: one “movement” regressor and the corresponding two “objective” parametric modulators (movement*QoM, movement*SoM), five regressors for each trial type (Offboard, Onboard, Joint, Disjoint, and Static) and the corresponding six “subjective” parametric modulators (offboard*OMS, Onboard*SMS, joint* OMS, joint* SMS, Disjoint* OMS, Disjoint* SMS), plus one further regressor added to model response trials to the one‐back task.

Beyond controlling for motion inequalities across conditions, this analysis also allowed us to test the potential impact of motion parameters on neural activity. At the group level, the effect of objective (QoM and SoM) and subjective (SMS and OMS) motion parameters were defined as significant loading of the corresponding parameter estimate (tested through a series of one‐sample *t*‐tests across subjects), indicating that the BOLD response is significantly modulated by motion inequalities. For these analyses we applied a Bonferroni correction to account for multiple comparisons (*p* = .05/*N* = number of regions).

Note that standard methods of GLM parameter estimation automatically removes the effects of shared variability, so that each effect is adjusted for all others. Specifically, SPM software package automatically performs orthogonalization for parametrically modulated regressors, based on the order in which the regressors are specified in the model. In our case, the “unmodulated” regressors entered before the “modulated” regressors, so that the latter are automatically orthogonalized with respect to the former.

The model also included a temporal high‐pass filter, to remove low‐frequency confounds with a period above 128 s. Serial correlations in the fMRI time series were estimated with a restricted maximum likelihood (ReML) algorithm using an autoregressive AR(1) model during parameter estimation, assuming the same correlation structure for each voxel, within each run. The ReML estimates were then used to whiten the data.

**Figure 4:**
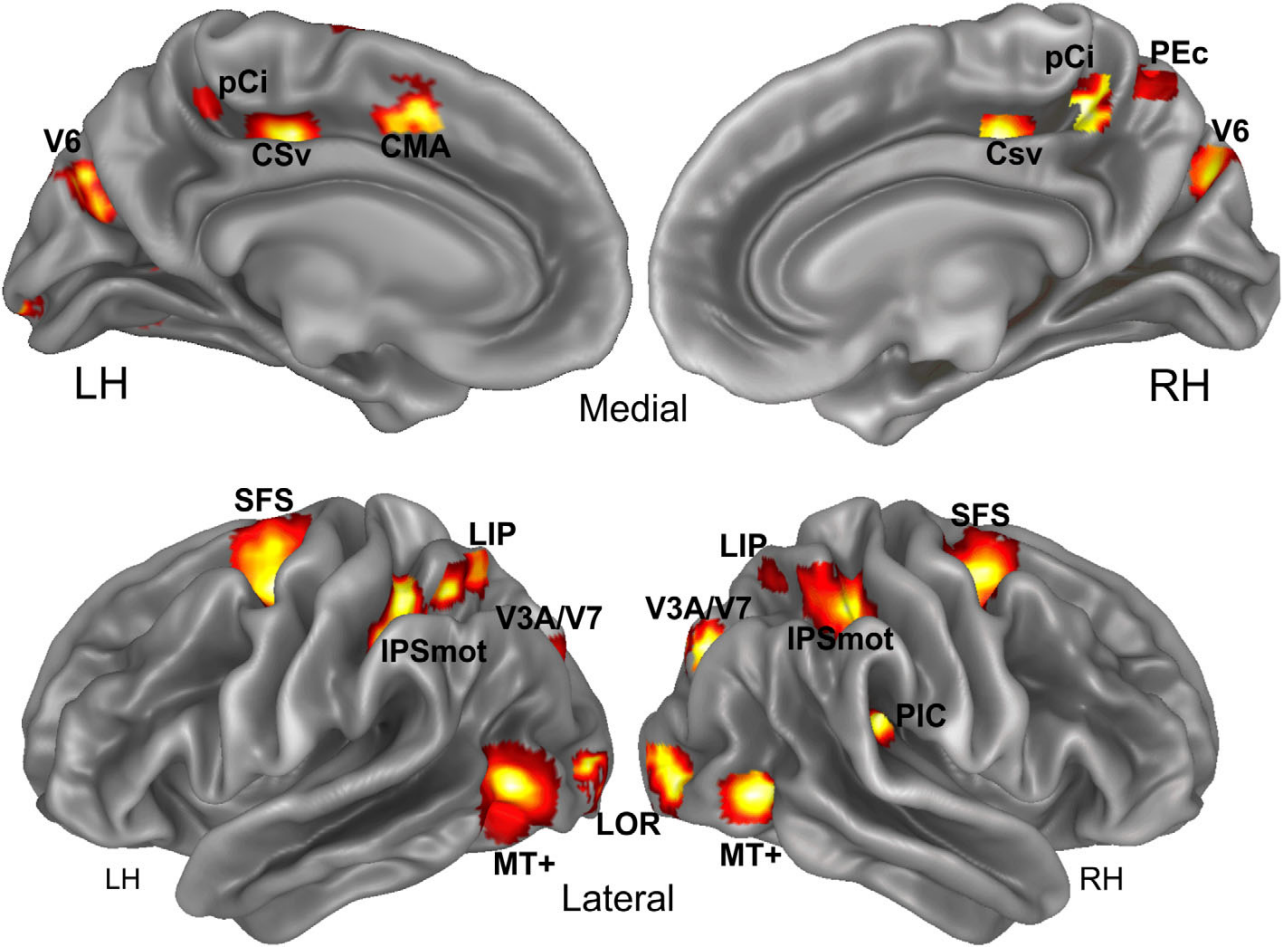
Motion areas. In color are the cortical regions more activated in at least one of the motion conditions relative to the static condition: V6, PEc, pCi, CSv (visual cingulate sulcus), CMA (cingulate motor area), MT+ (middle temporal complex), LOR (lateral occipital region), PIC (parietal insular cortex), V3A/V7, LIP (lateral intraparietal), IPSmot (intraparietal sulcus motion area), SFS (superior frontal sulcus region). Results are displayed on the medial and lateral folded representation of the right and left hemispheres of the template brain

These subject‐specific models were used to compute a set of contrast images per subject, each representing higher estimated amplitude of the hemodynamic response in one trial type as compared to the fixation baseline. Contrast images from all subjects were entered into a within‐subjects ANOVA with nonsphericity correction, where subjects were considered as a random effect, thus allowing to draw inferences related to the whole population our participants were extracted from. This model was used to search the whole brain for regions differentiating any of the four motion conditions (Offboard, Onboard, Joint, and Disjoint movies) from the Static condition (static frames). The resulting statistical parametric map of the *F* statistics was thresholded at the voxel level and by cluster size. Correction for multiple comparisons was performed using approximations from the Gaussian field theory (*p* < .01 FWE; extent threshold = 20 voxels). The resulting regions are listed in Table 1 and rendered in Figure 4, and include all voxels showing a reliable positive BOLD response during motion relative to static frames, irrespective of the kind and amount of motion. In‐house software (BrainShow, written in Matlab) was used to visualize the resulting regions onto a population‐average, landmark‐ and surface‐based (PALS) atlas (Van Essen, 2005), and to assign anatomical labels to activated areas at the level of Brodmann areas and cortical gyri. Brodmann areas were derived from the Talairach Daemon public database (Lancaster et al., 2000), while cortical gyri were derived from a macroscopical anatomical parcellation of the MNI single‐subject brain (Tzourio‐Mazoyer et al., 2002).

**Table 1:** MNI coordinates (mm) and sizes (mm^3^) of cortical regions identified in the group whole‐brain analysis comparing all motion conditions versus the static condition

	Left hemisphere				Right hemisphere				
Areas	X	Y	Z	Size	X	Y	Z	Size
PEc	[−]	[−]	[−]	[−]	9	−58	58	513
pCi	−18	−43	52	702	9	−43	49	837
CSv	−12	−19	43	2,241	12	−19	40	1,242
CMA	−9	17	46	972	[−]	[−]	[−]	[−]
PIC	[−]	[−]	[−]	[−]	39	−31	19	540
LOR	−24	−94	1	972	33	−88	−2	1,647
MT+	−42	−70	1	7,074	45	−67	−2	2,106
V6	−21	−79	31	1,107	18	−82	34	1,593
V3A/V7	−12	−85	34	1,350	30	−82	37	729
IPSmot	−30	−40	49	2,565	33	−40	49	3,645
LIP	−30	−52	55	1,566	18	−58	55	864
SFS	−21	−7	52	6,750	24	−4	55	5,427

*Note*: We report the peak coordinates of the corresponding cluster.

After identifying the regions differentiating motion from static frames, we searched for modulation of BOLD responses in these regions as a function of the movie type after controlling for motion inequalities across conditions. These steps were performed on regionally averaged data as follows. Each cluster of significantly activated adjacent voxels in the motion versus static group statistical map described above constituted a region to be analyzed. For each subject and region, we computed a spatial average (across all voxels in the region) of the preprocessed time series and derived hemodynamic response estimates for all parameters in the models. Finally, the regional hemodynamic responses, which are shown in the plots in Figure 4, were analyzed separately for each region by means of one‐way ANOVAs with condition (Offboard, Onboard, Joint, and Disjoint) as factor. In this ANOVA, we used Duncan test for conducting post‐hoc comparisons. Note that, although the analysis used to define the regions and the selective analysis conducted on the regionally averaged data are based on the same data set, they are inherently independent. The first step tests for the presence of any motion‐related neural response regardless of the kind and of the amount of motion, while the second step tests for modulations induced by the kind and by the amount of motion, thus avoiding the risk of “double dipping” (Kriegeskorte, Simmons, Bellgowan, & Baker, 2009).

#### 2.8.2 Functional localizer

Statistical analyses of functional images from the functional localizers used to define V6+, MT/MST+, and PPA/RSC ROIs are described in the Supporting Information. On these ROIs, we performed the same regional analysis as for the regions derived from the train experiment. Present data will be made available on request in compliance with the requirements of the funding institutes, and with the institutional ethics approval.

## 3 RESULTS

### 3.1 Behavioral results and motion parameters

Psychophysical data were collected during preliminary behavioral sessions, with stimuli identical to those in the functional MR session, but with the subjects that judge the degree of perceived movement after each stimulus with a self‐report measurement.

Figure 2d shows scores, averaged across subjects, for SMS (left side) in four conditions (Static, Onboard, Joint, Disjoint) and OMS (right side) in the same four conditions. Note that the SMS and OMS average scores are absent, respectively, in the Offboard and Onboard conditions, because these two conditions only include object‐ and self‐motion, respectively. Data were submitted to a separate one‐way ANOVA for SMS and for OMS with stimulus conditions as factor, where the three motion conditions (Onboard, Joint, Disjoint for SMS and Offboard, Joint, Disjoint for OMS) and the control static condition were considered as four levels of a single variable. The analyses were significant for SMS (*F*_[3, 39]_ = 173,2, *p* < .001) and for OMS (*F*_[3, 39]_ = 167,8, *p* < .001). For the SMS, a post‐hoc analysis revealed that subjects perceived higher self‐motion during Onboard than in the other three conditions (*p* < .001). In the Onboard trials, there was a first‐person real perspective which produce the typical retinal optic flow produced when the observer is moving through the environment and this likely led to more evidence of self‐displacement. Furthermore, subjects perceived equivalent self‐motion in Disjoint and in Joint condition (*p* = .159, n.s.). Finally, the SMS reported in the static condition (if any) was significantly lower than in any other condition (*p* < .001). Regard to OMS, the OMS reported in the static condition (if any) was significantly lower than any other condition (*p* < .001). Furthermore, subjects perceived identical OMS in Offboard, Joint and Disjoint conditions, supporting the hypothesis that motion sensation evoked by the train neither depends on target displacement on the screen nor on the type of object movements with respect to the subject.

We also aimed to test whether the perception of motion could be affected by the objective motion differences present across the various stimulus type. Thus, we correlated the subjective motion sensations with the objective motion parameters computed using the BMA (see Figure 2a–c and methods for details) to estimate the impact of using qualitatively different motion movies on the experienced vection sensation. Specifically, we tested the relationship between the subjective SMS and OMS and the two physical parameters (QoM, SoM) in each motion conditions (Offboard, Onboard, Joint, Disjoint), through Pearson's correlation analysis. The results of the correlations show that there was no relationship between QoM with SMS in Onboard (*r* = .3, *p* = .2), Joint (*r* = .3, *p* = .2), and Disjoint (*r* = −.1, *p* = .9) conditions, and between QoM with OMS in Offboard (*r* = −.8, *p* = .7) and Joint conditions (*r* = .3, *p* = .2). The results of the correlation between SoM with SMS in Onboard (*r* = −.2, *p* = .4), Joint (*r* = .3, *p* = .1), and Disjoint (*r* = .3, *p* = .1) conditions, and between SoM with OMS in Offboard (*r* = .3, *p* = .2) and Joint conditions (*r* = .3, *p* = .2) were not significant. Figure 2e shows the scatter plots where the *x*‐axis measures the QoM (left part) and SoM (right part) and *y*‐axis measures the SMS (squares) and OMS (triangles) reported by subjects in the Offboard (red), Onboard (blue), Joint (yellow), and Disjoint (green) condition. Each point represents one trial. The scatter plots present no correlation between the data sets, the points showing no significant clustering. This means that the two physical parameters (QoM and SoM) do not affect the SMS and OMS.

Overall, we aimed to test whether the perception of motion could be affected by the amount or the SoM, differently present across the various stimulus type. We conclude that the two classes of motion parameters are completely independent and that subjects perceived motion regardless of the strength of movement parameters.

### 3.2 Impact of motion measures on neural activity

We further explored whether each motion‐sensitive region was significantly modulated by the objective and subjective motion measures.

As to the objective motion measures, the QoM did not influence the activation in any of the observed regions (all *p* >.05, Bonferroni‐corrected), while the SoM gave a positive contribution to activation in LOR (*p* < .001) and a negatively contribution to activation in MT+ (*p* = .012) and V6+ (*p* = .036). Note that a positive and a negative relationship with SM can be interpreted as a preference for incoherent and for coherent motion, respectively.

As to the subjective motion measures, the amount of OMS experienced during the Offboard trials gave a positive contribution to activation in MT+ (*p* = .034) and V3A (*p* = .043). The only other significant relationship we found was a negative contribution of the amount of OMS experienced during the Disjoint trials (*p* = .015) on the V6+ (as defined by the localizer) activation. Any motion‐sensitive region was significantly modulated by the amount of SMS.

### 3.3 Imaging results

To reveal general differences in cortical areas specifically associated to the motion conditions, as a first step we selected regions showing greater fMRI responses in at least one of the motion conditions (Offboard, Onboard, Joint, and Disjoint) relative to the static condition. The rationale behind this approach is that we first wanted to isolate the areas responding to motion from other visual areas responding to the physical presence of the stimulus per se (i.e., sensorial response in early visual areas).

Results from the motion versus static (M‐S) contrast revealed significant activations in a network of 12 regions (V6, PEc, pCi, CSv, CMA, PIC, LOR, MT+, V3A/V7, IPSmot, LIP, and SFS) which are displayed on a semi‐inflated cortical surface reconstruction of the left and right hemispheres of an atlas brain (Figure 4). These areas cover a large cortical territory, spanning from the occipital, temporal and parietal to the insular and frontal cortex. The MNI coordinates of these regions are listed in Table 1.

As a second step, we defined a set of five ROIs using dedicated functional localizers. Specifically, we determined in individual subjects the objective position of areas MT and MST+ (Figure 7), V6+ (Figure 8), PPA and RSC (Figure 9) as mentioned in the Methods and detailed in the Supporting Information. The MNI coordinates of these regions are listed in Table 2.

**Table 2:** MNI coordinates (mm) and sizes (mm^3^) of ROIs defined in individual subjects using dedicated localizers

	Left hemisphere				Right hemisphere				
Areas	X	Y	Z	Size	X	Y	Z	Size
MT	−30	−73	6	1,342	43	−64	8	905
MST+	−35	−65	9	473	46	−57	14	627
V6+	−13	−78	30	1,141	16	−75	33	1,073
RSC	−17	−58	10	1,271	20	−56	12	1,483
PPA	−26	−50	−9	1,603	28	−49	−10	1,468

*Note*: We report the across subjects average of the centers of mass of individually defined regions.

Abbreviation: ROI, regions of interest.

**Figure 5:**
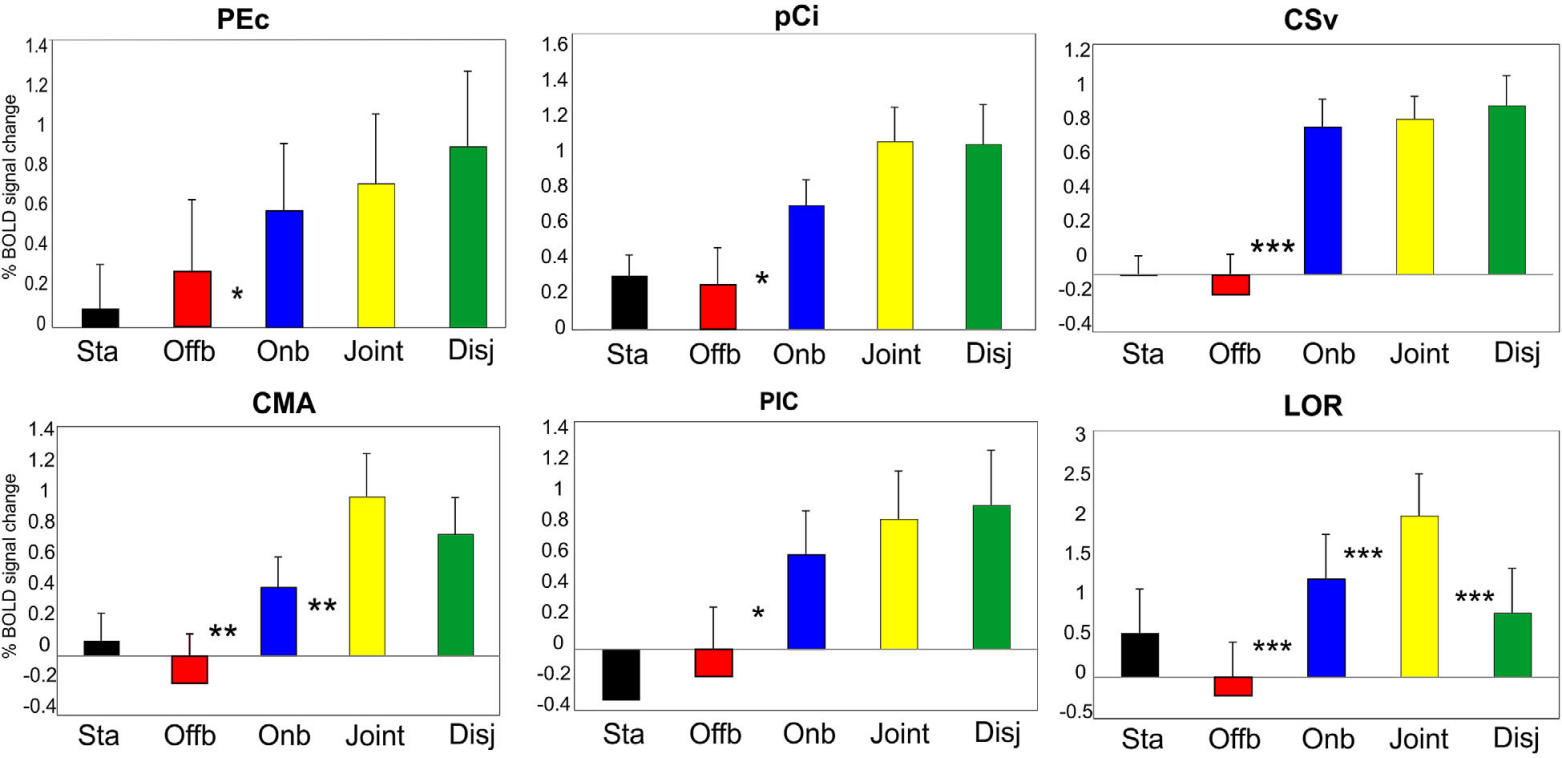
Motion areas preferring Onboard movies that induce self‐motion perception. The plots for each region represent the averaged BOLD percent signal change ± *SE* of the mean across subjects and hemispheres for each experimental condition: Static (Black), Offboard (Red), Onboard (Blue), Joint (Yellow), and Disjoint (Green). Name abbreviations for some of the conditions are as follows: Sta (Static), Offb (Offboard), Onb (Onboard), Disj (Disjoint). Significant comparisons are also reported. *p < .05; **p < .01; ***p < .001. Note that we refer to the BOLD percent signal change as the percentage of signal change with respect to a fixed value (grand mean scaling = 100), not scaled with respect to the specific voxel, as implemented in SPM. LH, left hemisphere; RH, right hemisphere

**Figure 6:**
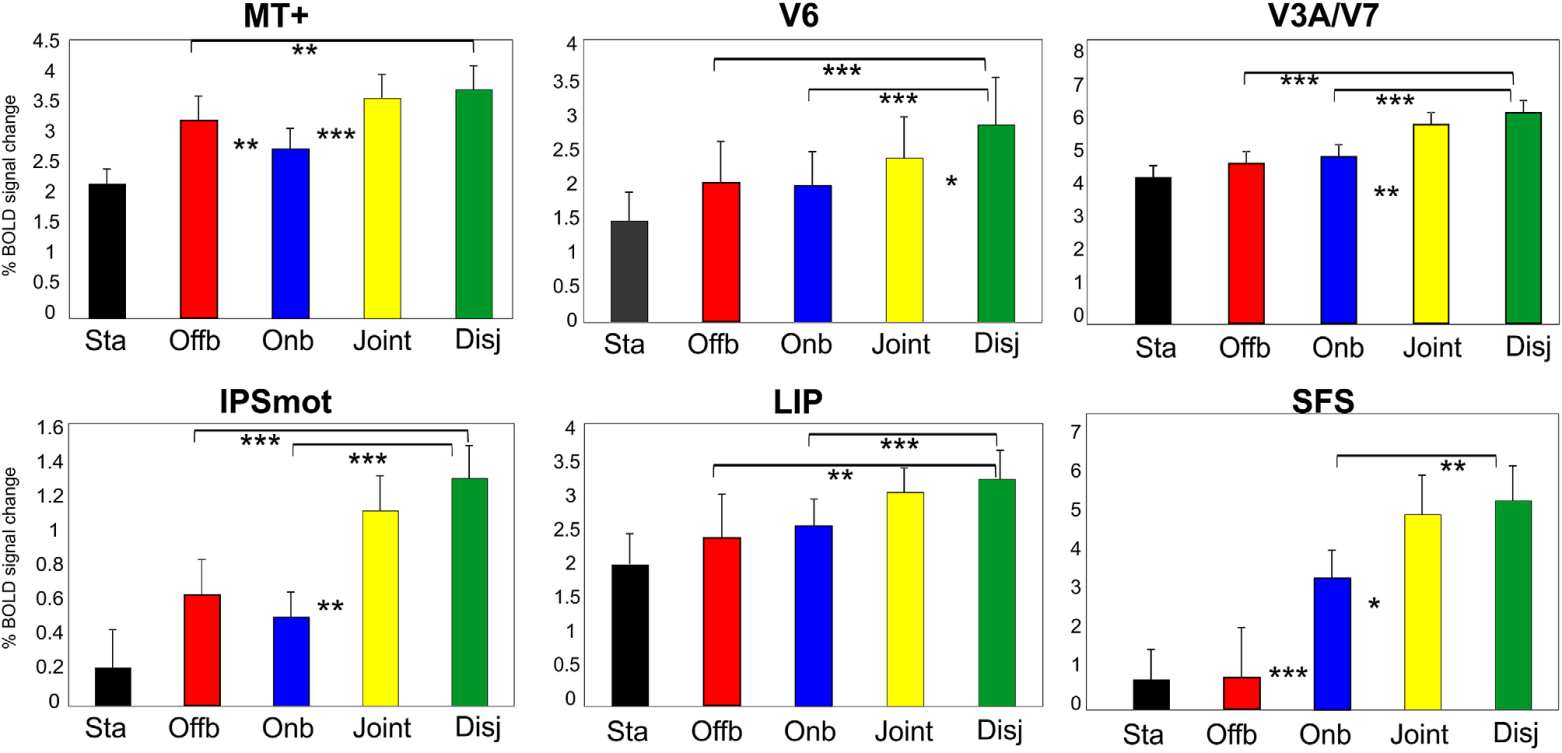
Motion areas preferring Offboard movies (inducing object‐motion) or Disjoint movies (inducing object‐ and self‐motion). Name abbreviations for some of the conditions are as follows: Sta (Static), Offb (Offboard), Onb (Onboard), Disj (Disjoint). Significant comparisons are also reported. *p < .05; **p < .01; ***p < .001. Other details and logos are as in Figure 4

**Figure 7:**
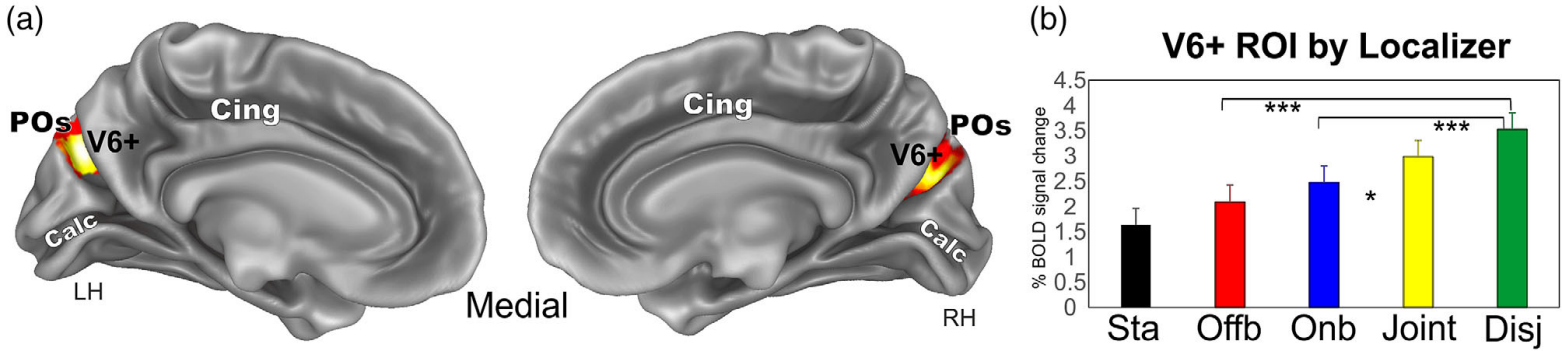
Areas MT and MST+ mapped by functional localizer (i.e., ipsilateral vs. contralateral radial motion). (a and b) MT (red; *N* = 16Hs) and MST+ (dark blue; *N* = 15Hs) ROIs are displayed on the lateral inflated cortical surface reconstruction of the left hemisphere of seven representative participants. (c) Plots represent the averaged BOLD percent signal changes ± *SEM* in the localizer‐defined areas MT and MST+. Name abbreviations for some of the conditions are as follows: Sta (Static), Offb (Offboard), Onb (Onboard), Disj (Disjoint). Significant comparisons are also reported. *p < .05; **p < .01; ***p < .001. (d) The overlap of the individually defined MT and MST+ ROIs displayed on the lateral folded representation of the right and left hemispheres of the template brain. The fundus of the main sulci is labeled: ITs, inferior temporal sulcus; MTs, middle temporal sulcus; ROI, regions of interest; STs, superior temporal sulcus; hIPS, horizontal segment of the intraparietal sulcus; pIPS, posterior segment of the intraparietal sulcus; PCs, postcentral sulcus; Cs, central sulcus, LOs, lateral occipital sulcus

**Figure 8:**
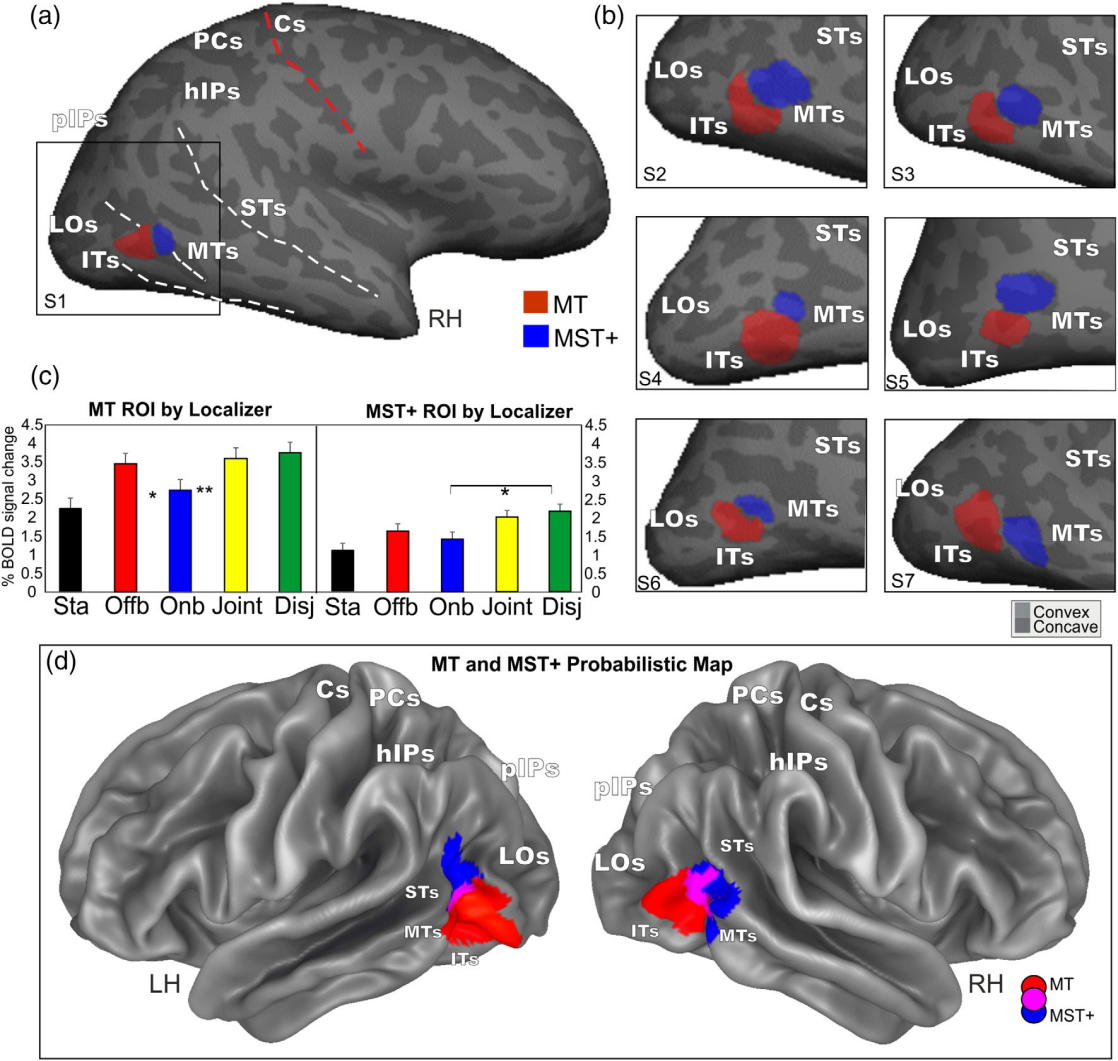
Area V6+ mapped by functional localizer (i.e., coherent flow vs. randomly moving dots). (a) The overlap of all the individually defined V6+ ROIs (*N* = 28 Hs) is displayed on the medial folded representation of the right and left hemispheres of the template brain. The fundus of the main sulci is labeled: POs, parieto‐occipital sulcus; Calc, calcarine sulcus; Cing, Cingulate cortex. (b) Plots represent the averaged BOLD percent signal changes ± *SEM* in the localizer‐defined area V6+. Significant comparisons are also reported. *p < .05; **p < .01; ***p < .001. Name abbreviations for some of the conditions are as follows: Sta (Static), Offb (Offboard), Onb (Onboard), Disj (Disjoint). ROI, regions of interest

**Figure 9:**
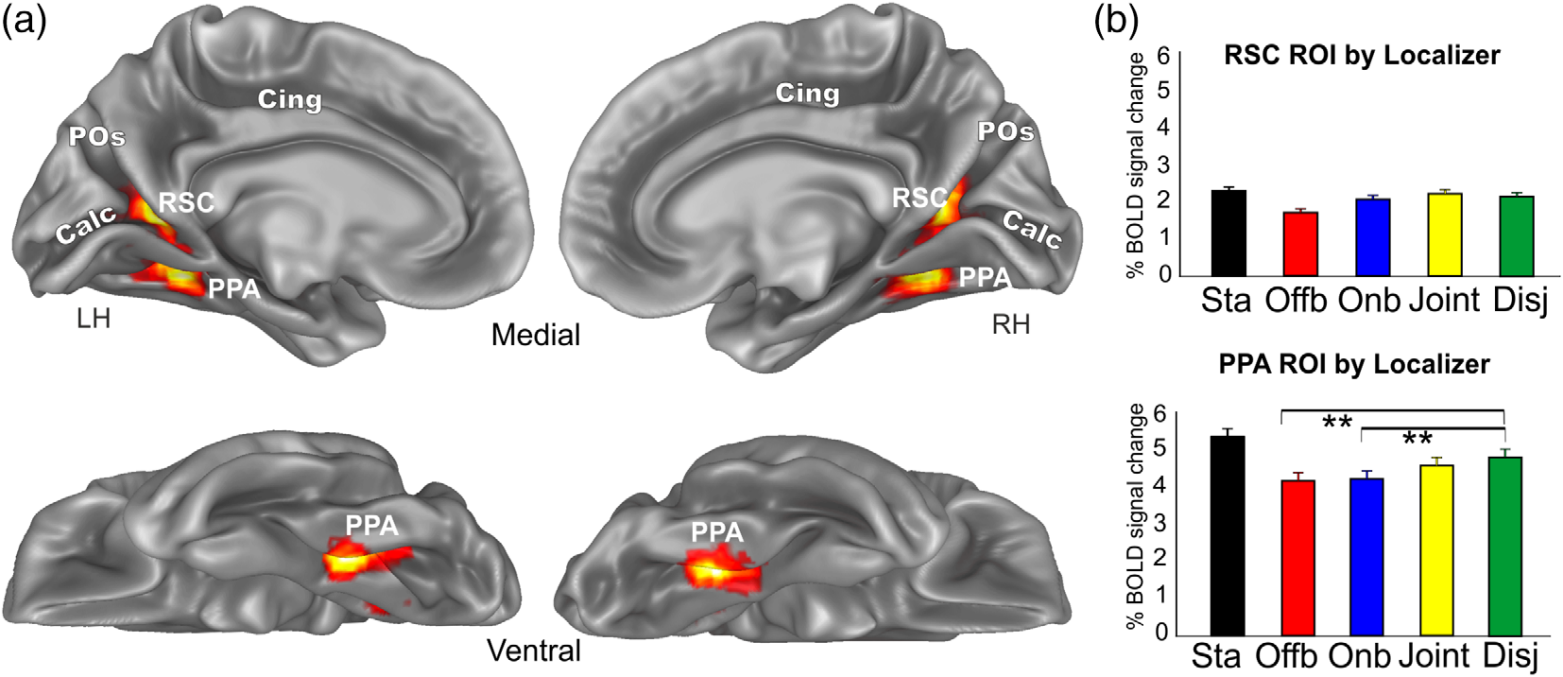
Areas PPA and RSC mapped by functional localizer (i.e., places vs. faces). (a) The overlap of all the individually defined PPA ROIs (*N* = 26 Hs) and RSC (*N* = 26 Hs) is displayed on the medial and ventral folded representation of the right and left hemispheres of the template brain. The fundus of the main sulci is labeled: POs, parieto‐occipital sulcus; Calc, calcarine sulcus; Cing, Cingulate cortex. (b) Plots represent the averaged BOLD percent signal changes ± *SEM* in the localizer‐defined areas PPA and RSC. Significant comparisons are also reported. *p < .05; **p < .01; ***p < .001. Name abbreviations for some of the conditions are as follows: Sta (Static), Offb (Offboard), Onb (Onboard), Disj (Disjoint). PPA, parahippocampal place area; ROI, regions of interest; RSC, retrosplenial complex

As a third step, we studied the functional response profile of the resulting 17 regions (V6 [V6+], PEc, pCi, CSv, CMA, PIC, LOR, MT+ [MT and MST+], V3A/V7, IPSmot, LIP, SFS, PPA, and RSC) to explore their sensitivity to different types of motion conditions. The mean percentage signal changes we observed in the motion and static conditions relative to the fixation baseline are plotted in the column histograms of Figures 5, 6, 7, 8, 9. Although the static condition did not enter in the regional analysis, the signal change obtained in this condition was plotted to illustrate the amount of motion responsiveness of the observed regions to this condition. Statistical results of this analysis are also detailed in Tables S1 and S2.

We were particularly interested in verifying some specific contrasts. First, we compared Onboard versus Offboard conditions (in histograms, blue vs. red), to reveal specific preferences for pure self‐ and object‐motion. Second, we compared the preferred condition of a specific region (i.e., Onboard, Offboard, or both in case of no preference) versus the Disjoint condition (in histograms, blue/red vs. green), to reveal preferences for a more complex and ecological condition. Note that in the Disjoint condition self‐ and object‐motion coexist but are independent of each other (i.e., the subject moves in one direction and the train move as well, but in another direction). In this respect, the Disjoint condition is the most complex one, similarly to what happens in daily life where people move in a dynamic and complex environment.

Third, we compared Joint versus Onboard (in histograms, yellow vs. blue), to reveal cortical areas able to extract object‐motion from the overall motion perceived on the screen. As described in the Methods, the Joint condition is extremely interesting because although it is a combination of self‐ and object‐motion (both the subject and the train are moving along the same direction and at the same velocity), the train has a fixed position on the screen throughout each movie. In the real world, when one's head moves, the image of a stationary object will move across the retina. If the head is moving yet the retinal image is static, the object must be moving (in synchrony with the head). In the Joint condition, we perceive the train (which is fixed on the screen) as moving and the external environment (which is shifting on the screen) as static. This means that in this specific condition, the perception of object‐motion (i.e., the train movement) is not simply the result of retinal/screen image motion (as generally supposed) but must be inferred (i.e., extracted) from the overall motion after estimating and subtracting out self‐motion information (i.e., the subject motion). Thus, a difference between Joint and Onboard conditions is an index of real motion extraction given that the two conditions have the same quantity of self‐motion.

The anatomical location and the functional profile of each region are described in detail below. Regions are grouped here and in Discussion by their functions independently to their anatomical position.

### 3.4 Regions preferring Onboard movies (inducing self‐motion perception)

#### 3.4.1 Area PEc

This region is located in the anterior precuneus just posterior to the dorsal tip of the cingulate sulcus (Figure 4). We found activation in this region only in the right hemisphere. This position, as well as the mean coordinates of this region (Table 1), well corresponds to the position of the newly define human homolog of macaque area PEc (Pitzalis et al., 2019). This position corresponds also to the PCu region found by Huang et al. (2015) and Fillimon, Rieth, Sereno, and Cottrell (2015). Plots in Figure 5 show that area PEc responds more to Onboard than Offboard.

#### 3.4.2 Area pCi

This region is located within the caudal part of the cingulate sulcus, in the ascending arm of this sulcus (Figure 4). This region was originally labeled Pc (as Precuneus) by Cardin and Smith (2010), but then also the same authors referred to it as the precuneus motion area (PcM) to distinguish it from other parts of the precuneus (Cardin & Smith, 2011; Uesaki & Ashida, 2015; Wada, Sakano, & Ando, 2016). Although the region found here strictly corresponds to area Pc (Cardin & Smith, 2010, 2011; see also Table 1 for MNI comparisons), here we prefer to call it posterior Cingulate (pCi) area to highlight its correct location within the cingulate sulcus. Plots in Figure 5 show that area pCi responds more to Onboard than Offboard.

#### 3.4.3 Area CSv

This region is located in the depth of the posterior part of the cingulate sulcus, anterior to the ascending portion of the cingulate sulcus (also called the marginal ramus of the cingulate sulcus; Figure 4). This location, as well as the mean coordinates of this region (Table 1), corresponds well to the original definition of human CSv provided by Wall and Smith (2008). Plots in Figure 5 show that area CSv responds more to Onboard than Offboard.

#### 3.4.4 Area CMA

We found significant activity in the middle portion of the cingulate gyrus (only in the left hemisphere; Figure 4). This region is still located in the cingulate cortex, but it is more anterior than CSv. Moreover, while CSv lies in the fundus of the cingulate sulcus, this region lies in the dorsal bank of the sulcus. It is slightly superior in terms of stereotaxic coordinates and overlaps part of the superior frontal gyrus. The position as well as the mean coordinates of this region (Table 1) corresponds to the cingulate motor area (CMA) which lies inferior to the presupplementary motor area (Amiez & Petrides, 2014; Picard & Strick, 1996) and that has been recently found as being motion sensitive (Field, Inman, & Li, 2015). Thus, here we refer to this region as CMA. Plots in Figure 5 show that area CMA responds more to Onboard than Offboard and more to Joint than Onboard.

#### 3.4.5 Area PIC

We found a significant area of signal increase at the junction between the parietal and the insular cortex, extending from the lateral sulcus into the posterior part of the insula (PIC). We found activation in this region only in the right hemisphere (Figure 4). Originally, a region in this location of the brain was called PIVC. It was a multisensory region in macaque (Grüsser, Pause, & Schreiter, 1990; Guldin & Grüsser, 1998), localized in humans with vestibular (Bottini et al., 1994; Brandt, Dieterich, & Danek, 1994; Bucher et al., 1998; Dieterich et al., 2003; Eickhoff, Amunts, Mohlberg, & Zilles, 2006; Fasold et al., 2002; Friberg, Olsen, Roland, Paulson, & Lassen, 1985; Indovina et al., 2005), or visual stimuli (Cardin & Smith, 2010). But recent evidence has showed that along the insula in humans there are two motion regions, named PIVC and PIC (Greenlee et al., 2016). PIVC is located more anteriorly, in correspondence of the lateral end of the central sulcus. PIC is located more posteriorly, in correspondence of the dorsal tip of the posterior end of the insula, entering the border between the supramarginal and angular gyri. While PIC is a multisensory region, responding to both vestibular and visual stimuli, PIVC responds to vestibular stimuli only. Thus, previously reported activations in posterior lateral sulcus during self‐motion induced by visual motion (Cardin & Smith, 2010; Huang et al., 2015; Uesaki & Ashida, 2015) might fall within PIC, or at least partially overlap with PIC, rather than PIVC (Frank et al., 2014; Frank, Sun, et al., 2016; Frank, Wirth, & Greenlee, 2016; Frank & Greenlee, 2014; Greenlee et al., 2016). Here, as we used a pure visual stimulation, we refer to this region as PIC area. Notice that the mean coordinates of this region (Table 1) and its anatomical position (Figure 4) strictly correspond to those provided in the original paper by Cardin and Smith (2010). Plots in Figure 5 show that area PIC responds more to Onboard than Offboard.

#### 3.4.6 Area LOR

This area is located on the lateral occipital sulcus, between dorsal V3A and MT+. The LOR position (Figure 4; Table 1) corresponds to a region where motion‐selective response for various types of moving stimuli was found in many previous fMRI studies (Dupont et al., 1997; Georgieva, Todd, Peeters, & Orban, 2008; Larsson & Heeger, 2006) and by our group (Pitzalis et al., 2010; Pitzalis, Strappini, De Gasperis, Bultrini, & Di Russo, 2012). The LOR is also part of the kinetic occipital motion‐sensitive region described by Orban's group (Van Oostende, Sunaert, Van Hecke, Marchal, & Orban, 1997). In absence of a retinotopic mapping, area LOR was defined here based on anatomical position, MNI coordinates, and neighboring relations with other areas. In addition, we showed in Figure S1 the overlay between LOR and the Conte69 surface‐based atlas (Van Essen, Glasser, Dierker, Harwell, & Coalson, 2011). This overlay suggests that this region overlaps retinotopic dorsal areas V3 (Sereno et al., 1995) and LO1/LO2 (Larsson & Heeger, 2006) which is also the typical reported position for the occipital place area (Sulpizio, Boccia, Guariglia, & Galati, 2018). Plots in Figure 5 show that LOR responds significantly more to Onboard than Offboard and to Joint than Disjoint. It responds more to Joint than Onboard.

### 3.5 Regions preferring Offboard movies (inducing object‐motion) or Disjoint movies (inducing object‐ and self‐motion)

#### 3.5.1 Area MT+ and its functional subdivisions MT and MST+

The mean MNI coordinates of MT+ (Table 1; Figure 4) are in good agreement with those of the classic motion‐sensitive region MT+ described in earlier studies using both PET (*x* = ±42, *y* = −69, and *z* = 0; Watson et al., 1993) and fMRI (*x* = ±45, *y* = −76, and *z* = +3; Tootell et al., 1995).

It is now generally acknowledged that the large motion‐sensitive region MT+ is a complex of several areas (Kolster, Peeters, & Orban, 2010; Pitzalis et al., 2010), which are also referred to as TO1 and TO2 (Wandell & Winawer, 2011). The subregions in the human MT+ complex surely include areas as MT and MST, which have different functional profiles and could be differently involved in egomotion perception (Fischer et al., 2012; Pitzalis, Sdoia, et al., 2013; Smith, Wall, Williams, & Singh, 2006; Wall & Smith, 2008). To check the preference of these two areas to different types of motion conditions used here, we mapped MT and MST+ using an independent functional localizer following standard procedures as described in the Methods (Figure 3b) and in many previous papers (Dukelow et al., 2001; Huk et al., 2002; Smith et al., 2006; Wall & Smith, 2008). The two regions resulting from this analysis are rendered in Figure 7a,b. The mean MNI coordinates of MT region and those of MST+ region (see Table 2) are similar to those provided in Kolster et al. (2010), respectively, for MT/V5 and pMSTv. Figure 7a,b shows the position of the two regions in the cortical surface reconstruction of the left hemisphere of some representative individual participants. Figure 7d shows the overlap of the individually defined MT and MST+ ROIs displayed on the template brain. MT (red) and MST+ (dark blue) regions occupy an anatomical position in between the inferior temporal sulcus (ITs) and the middle temporal sulcus (MTs), which is in line with the description of these two regions provided in previous fMRI studies where the two motion areas have been distinguished (Dukelow et al., 2001; Huk et al., 2002; Smith et al., 2006). Area MST+ (dark blue) was always anterior and often dorsal to MT, although there was some degree of variability across subjects. A constant evidence was that MST+ typically abutted MT, as previously reported by Huk et al. (2002). Plots in Figure 6 show that area MT+ responds more to Offboard than Onboard. It responds more to Disjoint than Offboard and more to Joint than Onboard. Furthermore, this region is so motion‐sensitive that, as area V6, responds more to *any* type of motion than to static stimuli. Plots in Figure 7c show that the localizer‐defined area MT responds more to Offboard than Onboard and more to Joint than Onboard. Conversely, the localizer‐defined area MST+ responds more to Disjoint than Onboard.

#### 3.5.2 Area V6

The position of this region (Figure 4) well corresponds to the location of human area V6 as defined in Pitzalis et al. (2006, 2010, 2012, 2015), Pitzalis, Bozzacchi, et al. (2013), Pitzalis, Fattori, and Galletti (2013), Pitzalis, Sdoia, et al. (2013), Pitzalis, Sereno, et al. (2013). The area found here is indeed located on the dorsal margin of the POs in correspondence of its posterior bank and has MNI coordinates (Table 1) well compatible with those of the originally defined V6 (Pitzalis et al., 2006, 2010). To check whether this region corresponds to area V6, we independently defined area V6+ according to the functional localizer (see Section 2 and Figure 3a) in all scanned subjects. The map found with the localizer in 28/28 hemispheres (Figure 8a) has MNI coordinates (Table 2) and position which closely resemble that obtained with the contrast M‐S (Figure 4). The results (plot in Figure 6) show that area V6 (as defined by the motion vs. static contrast) responds more to Disjoint than to any other condition and shows a tendency to respond more to Joint than Onboard, which however does not reach statistical significance. Furthermore, this region is so motion‐selective that responds more to *any* type of motion than to static stimuli. Plots in Figure 8b show that area V6+ (as defined by the localizer) has a functional profile very similar to that observed in Figure 6. In fact, it responds quite strongly but indifferently to Onboard and Offboard, although there is a tendency to respond more to Onboard than Offboard which however does not reach statistical significance. It responds more to Disjoint than any other condition. Furthermore, it responds more to *any* type of motion than to static stimuli. Interestingly, area V6+ as defined by the localizer responds more to Joint than Onboard, like the V6 region described above, but here the difference is statistically significant. This difference in the functional profile could be due to the more refined V6 localization obtained by the localizer, which includes only voxels touching the POS.

#### 3.5.3 Areas V3A/V7

This region, located in the ventral portion of the posterior intraparietal sulcus (pIPS; Figure 4) likely corresponds to the dorsal visual area V3A (Table 1; Tootell et al., 1997). The activation is located posteriorly and laterally to that of the V6 region, bordering its posterior part. In absence of a retinotopic mapping, V3A was defined here based on anatomical position, MNI coordinates and neighboring relations with other areas. In addition, we showed in Figure S1 the overlay between V3A and the Conte69 surface‐based atlas (Van Essen et al., 2011). This overlay suggests that this region seems to overlap mainly the retinotopic dorsal area V7 (Tootell et al., 1998) encompassing also area V3A, especially in the left hemisphere (Sereno et al., 1995), but not V3B (Smith, Greenlee, Singh, Kraemer, & Hennig, 1998). See Supporting Information for further details. While motion signals in V7 have been rarely (if any) reported, V3A is a consolidated dorsal motion area reported in several fMRI papers (Cardin & Smith, 2011, Cardin, Hemsworth, & Smith, 2012; Fischer et al., 2012; Helfrich, Becker, & Haarmeier, 2013; Pitzalis et al., 2010, 2012, 2013; Serra et al., 2019; Wall, Lingnau, Ashida, & Smith, 2008). For these reasons, we prefer being conservative labeling the region V3A/V7. Plots in Figure 6 show that the V3A/V7 region responds more to Disjoint than Offboard and Onboard, and more to Joint than to Onboard.

#### 3.5.4 Area IPSmot

This parietal region is located along the horizontal segment of the IPs (Figure 4) and may correspond to the human area VIP. In absence of a retinotopic mapping, IPSmot region was defined here based on anatomical position, MNI coordinates and neighboring relations with other areas. Four different locations have been obtained for VIP in humans by Bremmer et al. (2001), Sereno and Huang (2006), Bartels et al. (2008), and Cardin and Smith (2010), respectively. Thus, given that in absence of monkey fMRI data the homology question cannot be settled, we choose the neutral name of intraparietal sulcus motion region (IPSmot) as already done in another previous paper from our lab (Pitzalis, Sdoia, et al., 2013). The mean coordinates of this region (Table 1) and its anatomical position (Figure 4) are in line with those of the original IPSmot (Pitzalis, Sdoia, et al., 2013) which is however more lateral and slightly posterior than area VIP as described in other fMRI studies (Cardin & Smith, 2010; Huang et al., 2015; Sereno & Huang, 2006). Plots in Figure 6 show that area IPSmot responds equally well to Onboard and Offboard. It responds more to Disjoint than Offboard and Onboard and more to Joint than Onboard.

#### 3.5.5 Area LIP

This region is located in the dorsal portion of the intraparietal sulcus (IPS). Specifically, it is situated in the cortical region joining the horizontal segment of the IPS and the pIPS, anteriorly to V3A. The mean coordinates of this region (Table 1) and its anatomical position (Figure 4) corresponds to the position of the retinotopic area LIP provided in the original paper by Sereno, Pitzalis, and Martinez (2001). Other fMRI studies found a human homolog of macaque LIP in a similar position using saccades versus reaching paradigm (Galati et al., 2011, LD area; Schluppeck, Curtis, Glimcher, & Heeger, 2006; Hagler Jr, Riecke, & Sereno, 2007; Swisher, Halko, Merabet, McMains, & Somers, 2007; Tosoni, Galati, Romani, & Corbetta, 2008, pIPS area). In absence of a retinotopic mapping, LIP region was defined here based on anatomical position, MNI coordinates and neighboring relations with other areas. In addition, we showed in Figure S1 the overlay between this region and the Conte69 surface‐based atlas (Van Essen et al., 2011). This overlay suggests that this region overlaps mainly the retinotopic fields IPS3‐4, thus surely including the human homolog of area LIP (Schluppeck, Glimcher, & Heeger, 2005; Sereno et al., 2001; Silver, Ress, & Heeger, 2005; Swisher et al., 2007). Plots in Figure 6 show that area LIP responds equally well to Onboard and Offboard and it responds more to Disjoint than Offboard and Onboard.

#### 3.5.6 Area SFS

This region is located in the superior frontal sulcus (SFS). The mean coordinates of this region (Table 1) and its anatomical position (Figure 4) correspond to the SFS motion region recently described by Huang et al. (2015). This region partially overlaps with the superior part of the frontal eye fields (FEF) region, still located on the precentral sulcus but more inferiorly (see Huang et al., 2015 for a strict comparison between FEF and SFS). Plots in Figure 6 show that SFS responds more to Onboard than to Offboard. It responds more to Disjoint than Onboard. Finally, it does discriminate between Joint and Onboard.

### 3.6 Motion responses in ventral scene‐selective areas

The reason why we decided to investigate the response of scene‐selective areas in this study is the recently increased interest in finding motion responses in scene‐selective regions using visual stimuli ecologically relevant for the human navigation (Korkmaz Hacialihafiz & Bartels, 2015; Schindler & Bartels, 2016). The general assumption behind this interest is that motion information is a dominant cue for scene reconstruction and spatial updating (Britten, 2008; Frenz, Bremmer, & Lappe, 2003; Medendorp, Tweed, & Crawford, 2003). Consequently, the neural activity in scene‐selective regions should be modulated by visual motion cues, especially when ecological stimuli representing realistic environment with a clear scene layout are used.

To check for the presence of motion responses in the scene‐selective areas, we mapped PPA and RSC using an independent functional localizer following standard procedures as described in Section 2 (see Figure 3c) and in many previous papers (Epstein, 2008; Sulpizio et al., 2013, 2014). Figure 9a shows the position of the two regions in the medial and ventral folded representation of the right and left hemispheres of the template brain. The mean coordinates of PPA (yellow) and RSC (orange) regions (Table 2) and their anatomical position (Figure 9) are very much in line with the description of these two regions provided in previous fMRI studies where the two scene‐selective areas have been distinguished (Epstein, 2008; Sulpizio et al., 2013, 2014). Note that we use the term RSC to follow the mainstream of current literature on scene selectivity, although it is well known that, from an anatomical standpoint, the human scene‐selective RSC is mainly located within the ventral portion of the POS and minimally extends anteriorly into the retrosplenial cortex proper (see Silson, Steel, & Baker, 2016).

As shown in the plots of Figure 9b, RSC and PPA have a very different functional profile. While RSC does not discriminate between any pair of conditions, PPA responds more to Disjoint than Offboard and Onboard, thus preferring complex combinations of self‐ and object‐motion. Neither PPA nor RSC are involved in the flow‐parsing phenomenon, being unable to distinguish Onboard from Joint.

## 4 DISCUSSION

Event‐related fMRI experiments, functional localizers, and brain mapping methods were used to study the sensitivity of several human cortical regions to movies simulating self‐ and object‐motion in a wide‐field realistic virtual environment. We used two sets of regions. The first set included regions (V6, PEc, pCi, CSv, CMA, LOR, LIP, SFS, IPSmot, V3A/V7, and MT+) that were defined through the “train” experiment, selecting regions showing greater fMRI responses in at least one of the motion conditions (Offboard, Onboard, Joint, and Disjoint) relative to the Static condition (contrast M‐S). The second set included ROIs that were defined through the functional localizers (V6+, MT/MST+, and PPA/RSC). Results show that some areas prefer movies inducing the perception of self‐ or object‐motion, whereas other areas prefer movies inducing the perception of complex visual stimulation (Disjoint) where both self‐ and object‐motion are present in the stimulus. We suggest that some of these areas may be involved in disentangling real object‐motion from self‐induced optical flow.

Psychophysical tests showed that subjects perceived motion in the stimuli used in this work regardless of the QoM present in them. As in everyday life, the movement of an object can be perceived even with the image of the object is still on the retina (when we pursue a moving object), and the immobility of objects can be the perceptual result of retinal image motions (when we move around in a structured environment). Given that, Onboard and Offboard conditions are able to evoke a pure SMS and OMS, respectively, hereafter we will use indifferently the terms self‐ or object‐motion to refer to our two conditions Onboard and Offboard, respectively.

In the following sections, we will first report a separate description of the main results achieved on human dorsal motion areas, then we will combine the evidence from macaque and human brain to suggest the possible functional role played by some of these regions in the flow‐parsing phenomenon.

### 4.1 Cortical areas preferring Onboard movies (inducing self‐motion perception)

We found a remarkable preference for pure self‐motion with respect to pure object‐motion in several medial and lateral cortical areas (PEc, pCi, CSv, CMA, PIC, and LOR) involved in the analysis of visual motion. All these regions showed a stronger response to Onboard than Offboard. These areas exhibited such a selective response to pure self‐motion that none of them responded more if object‐motion was also present in the stimulus. In fact, in none of these regions the BOLD signal obtained in Disjoint was significantly higher than that found in Onboard. Finally, four of these regions (CSv, CMA, PIC, and LOR) not only preferred self‐motion to object‐motion, but their mean response to Offboard condition, that is in presence of pure object‐motion, was negative.

Previous works have shown that CSv is sensitive to wide‐field egomotion‐compatible stimuli (Antal, Baudewig, Paulus, & Dechent, 2008; Cardin & Smith, 2010; Field et al., 2015; Fischer et al., 2012; Pitzalis, Sdoia, et al., 2013; Wada et al., 2016) and receives vestibular as well as visual input (Greenlee et al., 2016; Smith, Wall, & Thilo, 2012). We found (Pitzalis, Sdoia, et al., 2013) that CSv does not discriminate between the various types of egomotion‐compatible stimulations we used. Here, we found that CSv was not activated at all during the Offboard and the Static visual stimulations, that is by any type of visual stimulation that have not a vestibular input counterpart in physiological conditions. In line with Pitzalis, Sdoia, et al. (2013), present results show that CSv responds to all egomotion conditions independently from the presence of object‐motion, confirming the suggested role of CSv in self‐motion processing (Wall & Smith, 2008).

Like CSv, CMA, and PIC also show vestibular responses (Fasold et al., 2002; Frank, Sun, et al., 2016; Frank, Wirth, & Greenlee, 2016). It is known that the cingulate cortex participates in the cortical network monitoring head and body movements in space, as well as in visuospatial attention (Corbetta, Miezin, Shulman, & Petersen, 1993; Gitelman et al., 1999; Kim et al., 1999; Mesulam, 1999). It is therefore not surprising to find a CMA activation in our experimental conditions. However, to date, motion‐related responses in CMA in fMRI studies on egomotion had never been observed (Greenlee et al., 2016; Pitzalis et al., 2010; Pitzalis, Bozzacchi, et al., 2013; Pitzalis, Fattori, & Galletti, 2013; Pitzalis, Sdoia, et al., 2013). It could be that the extremely vivid SMS evoked by the naturalistic virtual reality movies and the wide‐field screen vision used here represents in our subjects a stronger input to CMA than standard visual stimuli such as random dots.

Area PIC in the Sylvian fissure is a motion region responding to visual and vestibular motion, presumably supporting the integration of motion information from visual and vestibular senses for the perception of self‐motion (Frank, Sun, et al., 2016; Frank, Wirth, & Greenlee, 2016). A recent study by Huang et al. (2015) found that the right PIC (there called PIVC) responds to active dodges suggesting that it plays an active role in sensing and guiding translational egomotion. Similarly, we also found a significant BOLD response only in the right PIC. This asymmetry is consistent with a right hemispheric dominance of the vestibular cortex in right‐handed subjects, as suggested by previous neuroimaging studies using visual, optokinetic, and vestibular stimuli (Dieterich et al., 2003; Dieterich, Bucher, Seelos, & Brandt, 1998).

LOR is part of the kinetic occipital motion‐sensitive region originally described by Orban's group (Van Oostende et al., 1997). In recent studies by our group we observed in LOR both a motion‐selective response for radially moving stimuli (Pitzalis et al., 2010) and a speed‐o‐topic organization (Pitzalis et al., 2012). Here, we found a remarkable preference of this region for self‐motion. In addition, it is worthwhile noting that LOR is the only motion area reading the difference between the two complex motion conditions (Joint and Disjoint), and highly preferring the Joint condition (where the object‐motion does not create any slip of the image on the screen).

Overall, we found that four regions prefer self‐motion, and in all of them, the mean response to pure object‐motion was negative. As already suggested (Field et al., 2015; Pitzalis et al., 2010), it could be that in physiological conditions an excitatory vestibular input activates the visuo‐vestibular areas (like CSv, CMA, and PIC) during self‐motion, whereas in motion conditions not implying egomotion and vestibular input (like in our Static and Offboard) the areas get inhibited. Present data are in support of this speculative hypothesis.

The PEc and pCi preference for self‐motion confirms the involvement of the precuneus and cingulate cortex in egomotion. These two areas are differently known in the egomotion literature. Since the discovery of the pCi (or pC/PcM) as a motion area by Cardin and Smith (2010), this region was frequently associated to the motion network (Greenlee et al., 2016) in that it responds to egomotion‐compatible stimuli (Cardin & Smith, 2010, 2011; Serra et al., 2019). Here, we confirm that pCi is a motion area responding to self‐motion more than object‐motion. The PEc, conversely, is a newly defined region that respond not only to leg and arm movements but also to flow field visual stimulation, as that used here to map area V6+. Monkey PEc has visual neurons preferring optic flow with curve trajectories compatible with heading changes (Battaglia‐Mayer et al., 2001; Raffi et al., 2002; Raffi, Carrozzini, Maioli, & Squatrito, 2010). In line with these previous studies, here using wide‐field naturalistic stimuli simulating continuous heading changes we have found in the PEc motion responsiveness and a reliable preference for self‐motion. This reinforce the hypothesis suggested in Pitzalis et al. (2019) that area PEc integrates visually derived self‐motion signals with motor leg movement with the aim of guiding locomotion. We have also shown a preference for pure self‐motion in the frontal SFS, although this region was not as selective as others were. Indeed, the area responded more if object‐motion was also present in the stimulus (Disjoint > Onboard) and thus will be discussed in the next section.

### 4.2 Selective preference for Offboard movies (inducing object‐motion perception) in MT

Only the lateral motion area MT (as defined by functional localizer) showed a preference for pure object‐motion. This area responded maximally to the Offboard condition, and significantly more to the Offboard than to the Onboard condition. The high preference of this region for the object‐motion is also supported by a positive correlation between its BOLD activity and the OMS experienced during the Offboard condition. MT exhibited such a selective response to pure object‐motion that the BOLD signal in Disjoint was not significantly higher than that found in Offboard. This means that self‐motion (present in Disjoint together with object‐motion) did not increase the BOLD signal in MT. Note that this selectivity for object‐motion found in MT was not present in MT+ as defined in the group analysis or in MST+, both responding more to complex conditions. This strengthens the need to separately refer to the two subdivisions (MT and MST) to avoid masking effects or lack of significance due to the average, which in some cases could cancel out possible differential effects.

### 4.3 Cortical areas preferring Disjoint movies (inducing object‐ and self‐motion)

We found several areas (V6, IPSmot, MST+, V3A, LIP, SFS, and PPA) responding more to complex visual stimulation (Disjoint), where both self‐ and object‐motion are present, than to pure object‐motion. However, these areas exhibited different behaviors in terms of preference for pure object and self‐motion, in that while SFS responded more to Onboard than Offboard, the other cortical areas (V6, IPSmot, MST+, V3A, LIP, and PPA) showed no preferences, responding equally well to both Offboard and Onboard conditions.

Human V6, like macaque V6, is a retinotopic motion area that responds to unidirectional motion (Fattori, Pitzalis, & Galletti, 2009; Pitzalis et al., 2006, 2010). It has a strong preference for coherent motion (Cardin & Smith, 2010; Helfrich et al., 2013; Pitzalis et al., 2010; von Pföstl et al., 2009) and a recent combined VEPs/fMRI work (Pitzalis, Bozzacchi, et al., 2013) has shown that V6 is one of the earliest stations (together with MT) coding motion coherence. Human V6 is highly sensitive to flow fields (Arnoldussen, Goossens, & van den Berg, 2011; Cardin, Hemsworth, & Smith, 2012; Cardin & Smith, 2010; Pitzalis et al., 2010) and is able to distinguish between different 3D flow fields being selective to translational egomotion (Arnoldussen, Goossens, & van Den Berg, 2015; Pitzalis, Sdoia, et al., 2013). The view that V6 is involved in the estimation of self‐motion has been confirmed also in other recent fMRI studies (Cardin, Hemsworth, & Smith, 2012; Cardin, Sherrington, et al., 2012; Cardin & Smith, 2010, 2011; Huang et al., 2015; Sherrill et al., 2015). Since macaque V6 contains many real‐motion cells, that is cells activated by real object movement but not by self‐evoked retinal image movements (Galletti & Fattori, 2003), we suggested (Pitzalis et al., 2010) that human V6 is involved in object‐motion recognition. This area could also process visual egomotion signals to extract information about the relative *distance* of objects, in order to act on them, or to avoid them, an hypothesis supported by its tight connectivity with areas involved in grasping (as V6A and MIP [Galletti et al., 2001; Galletti & Fattori, 2003]), its sensitivity to optic flow patterns combined with disparity cues, which are most informative for nearby objects (Cardin & Smith, 2011), and its reported preference to near‐field stimuli in humans (Quinlan & Culham, 2007).

Considering human and macaque data, we previously suggested that V6 is involved in both object‐ and self‐motion recognition (Pitzalis et al., 2010, 2015; Pitzalis, Bozzacchi, et al., 2013; Pitzalis, Fattori, & Galletti, 2013; Pitzalis, Sdoia, et al., 2013). The present results support this hypothesis, as V6 responds well to both self‐ and object‐motion, and highly prefers complex conditions where the two types of motion are simultaneously present (Disjoint condition). Among the several medial motion areas, V6 is the only one preferring complex visual motion.

The VIP (IPSmot) shares a similar functional profile with V6. All the recent neuroimaging results from our and other laboratories have demonstrated that human area VIP is a motion area involved in estimation of egomotion (Pitzalis et al., 2010; 2012; Cardin & Smith, 2010, 2011; Fischer et al., 2012). Additionally, like V6, VIP is able to distinguish between the different optic flow components, an evidence that agrees with the functional properties of macaque area VIP, whose neurons respond selectively to optical flow stimuli (Bremmer, Duhamel, Ben Hamed, & Graf, 2002; Colby, Duhamel, & Goldberg, 1993) and shows a strong response to translational egomotion (Pitzalis et al., 2013). Interestingly, area VIP was found to respond to looming objects (Huang, Chen, Tran, Holstein, & Sereno, 2012; Sereno & Huang, 2006) which explains its strong response to the approaching train present in Offboard. By the way, the greater response of this region for stimuli containing also self‐motion confirms previous human (Cardin & Smith, 2010, 2011; Peuskens, Sunaert, Dupont, Van Hecke, & Orban, 2001; Pitzalis, Sdoia, et al., 2013; Sereno & Huang, 2006; Wall & Smith, 2008) and macaque (Bremmer et al., 2001, 2002; Colby et al., 1993) findings suggesting that this area also processes optic flow and egomotion.

We found that area MST+, as defined by the functional localizer, is not differentially modulated by Onboard and Offboard, responding well to both conditions, and on average responding more to complex motion stimulation. The trend observed in MST+ deserves some comments. In a recent study (Pitzalis, Sdoia, et al., 2013) we showed that MST+ is able to distinguish between different 3D egomotion signals and is more sensitive to translational and radial egomotion than to the circular one. This preference was already observed in the past in both macaques (Duffy, 1998; Duffy & Wurtz, 1991; Eifuku & Wurtz, 1998; Graziano, Andersen, & Snowden, 1994; Nelissen, Vanduffel, & Orban, 2006; Orban et al., 1992; Saito et al., 1986; Tanaka, Fukada, & Saito, 1989; Tanaka & Saito, 1989) and humans (Kovács, Raabe, & Greenlee, 2008; Morrone et al., 2000; Smith et al., 2006; Wall et al., 2008). These past studies led to a general agreement in reporting MST as an area sensitive to the motion coherence and to egomotion signals (Arnoldussen et al., 2011; Helfrich et al., 2013; Kleinschmidt et al., 2002; Kovács et al., 2008; Morrone et al., 2000; Smith et al., 2006; Wall et al., 2008). However, recent studies failed to report positive evidence in favor of a role of MST+ in egomotion (Kleinschmidt et al., 2002; Wall & Smith, 2008) raising doubts about its effective importance in terms of egomotion perception. Although here we show that MST+ responds well to both self‐ and object‐motion, we also found that MST+ does not discriminate between Joint and Onboard and thus it does not seem to have a role in the flow parsing. We expected to find a more selective response profile in this area. In contrast, we found an overall general weaker BOLD signal than that found in MT (see Figure 6c) and a moderate MST+ preference for the self‐motion, meaning that this area responds to egomotion but is not strongly selective for it.

Area V3A is a retinotopic area whose motion sensitivity has been observed in several fMRI studies (Pitzalis et al., 2010; Sereno et al., 2001; Tootell et al., 1997; Wall & Smith, 2008). Many previous works on humans reported greater BOLD signal change in V3A for coherent than for random motion (Braddick, O'Brien, Wattam‐Bell, Atkinson, & Turner, 2000; Braddick et al., 2001; Moutoussis, Keliris, Kourtzi, & Logothetis, 2005; Vaina et al., 2003; but see Pitzalis, Bozzacchi, et al., 2013; Pitzalis, Sdoia, et al., 2013). Several other studies have shown that V3A is responsive to flow fields stimulation (Arnoldussen et al., 2011; Helfrich et al., 2013; Pitzalis et al., 2010; Sereno et al., 2001) and to other types of global motion signals, as those involved in reconstruction of form from motion (Orban, Sunaert, Todd, Van Hecke, & Marchal, 1999). Here, we show that area V3A responds to both object and self‐motion but even more to a complex visual stimulation where both types of motion coexist. Present results support the view of this area as a motion area processing egomotion signals.

Areas LIP and SFS/FEF are part of the dorsal attention network, invariably activated in studies on saccadic eye movements, visual tracking, or attention (covert or overt) shifts in both monkeys (Ben Hamed, Duhamel, Bremmer, & Graf, 2001, 2002; Koyama et al., 2004; Kubanek, Li, & Snyder, 2015; Wardak, Hamed, Olivier, & Duhamel, 2012) and humans (Astafiev et al., 2003; Corbetta et al., 1998; Culham et al., 1998; Perry & Zeki, 2000; Petit & Haxby, 1999; Schluppeck et al., 2005, 2006; Sereno et al., 2001). Although the activity of these two regions is mainly eye/attention‐movement related (see below for a full discussion on the role of eye movements), some of the previous works showed that the activity of these two regions is also related to visual motion (Huang et al., 2015; Pitzalis et al., 2010; Sunaert, Van Hecke, Marchal, & Orban, 1999). Present data support this view and show in detail that LIP and SFS/FEF are differently modulated by the various motion conditions used here: LIP responds indifferently to object‐ and self‐motion, SFS/FEF prefers pure self‐motion and both regions prefer complex motion.

We revealed the presence of some kind of motion sensitivity also in a ventral region, the PPA, which is typically not activated by visual motion in the absence of ecological scene‐like stimuli (Cardin & Smith, 2010; Greenlee et al., 2016; Pitzalis et al., 2010; Pitzalis, Sdoia, et al., 2013). The PPA, although not activated on average by motion conditions relative to the Static condition, revealed a preference for the Disjoint condition relative to Onboard and Offboard. This motion sensitivity of PPA is indirectly supported by recent evidence indicating that this area receives inputs from typical brain motion areas (Schindler & Bartels, 2016; Sherrill et al., 2015). In Tosoni et al. (2015), for instance, we found that human motion area V6 is functionally connected with PPA (see also Boccia, Sulpizio, Nemmi, Guariglia, & Galati, 2016 for a related finding). In line with Tosoni et al. (2015), unpublished data collected in Galletti's lab indicate that monkey V6 is directly connected with the ventral cortex within the occipitotemporal sulcus, a region that seems to be homologous to human PPA. The connections of V6 with area PPA support the view of a possible role of V6 in spatial navigation (Cardin & Smith, 2011; Pitzalis, Fattori, & Galletti, 2013; Pitzalis, Sdoia, et al., 2013).

Finally, we observed evidence of motion‐related responses also in the ventral part of the calcarine scissure, where Mikellidou et al. (2017) have recently identified the human prostriate area, a retinotopic region located medially in between RSC and PPA regions responsive to extremely fast motion over a wide visual field (see Supporting Information for details on the mapping procedures and Figure S2A for the anatomical position, MNI coordinates, and size of the region). The prostriate, although not activated on average by motion conditions relative to the Static condition as the PPA, revealed a preference for the Disjoint condition relative to Onboard (see Figure S2B). Like PPA, the prostriate is not involved in the flow‐parsing phenomenon, being unable to distinguish Onboard from Joint. Our results indicate that the prostriate, in line with its anatomical location, shows a functional profile intermediate between area PPA, which shows a preference for complex motion stimulation, and area RSc which is completely insensitive to any motion stimulation.

### 4.4 Neural basis of the flow‐parsing phenomenon

The neural basis of the flow parsing in humans is still a matter of debate (Arnoldussen et al., 2013; Billington & Smith, 2015; Fischer et al., 2012). Warren and Rushton (2009a, 2009b) have recently proposed that a flow‐parsing mechanism identifies and subtracts the optic flow associated with the observer's movement from the pattern of retinal motion, to estimate the true object‐motion. In monkeys, cells activated by real object movements but not by retinal image movements evoked by self‐movements were found (Galletti & Fattori, 2003). We postulated that the cortical areas containing these “real motion” cells represent the neural basis of the flow‐parsing mechanism (see Galletti & Fattori, 2018). Here, we investigated this phenomenon using a very peculiar motion condition called “Joint.” In this condition, the object‐motion is clearly perceivable by the subject although the image of the object is not moving on the retina. Conversely, the subject clearly perceives as motionless the objects whose images are moving on the retina because of self‐motion. Given that Joint has the same quantity of self‐motion as Onboard condition but only in Joint the subject perceives object‐motion, we speculate that a difference between the BOLD signal in Joint and Onboard conditions could be considered an index of the “real motion” extraction. In this respect, note that a higher response in the Joint condition cannot be attributable to a higher level of self‐motion perception because the psychophysical results revealed that subjects perceived more self‐motion in Onboard than in Joint.

We found a network of cortical areas more responsive to Joint than to Onboard and thus able to recognize the real movement in the visual field. Most of these are motion regions distributed in lateral and medial temporoparietal regions, and their functional in the other examined contrasts is heterogeneous, including regions preferring self‐motion (LOR), object‐motion (MT), and combined self and object‐motion (V6+, V3A, and IPSmot). Macaque and human results suggest that the flow‐parsing mechanism raises from a distributed and integrated network involving early and higher order regions having different functional properties and likely playing different roles in the visual motion processing. In humans, most of these flow parsing‐related regions are well known in the motion literature and have properties that, according to Warren and Rushton (2009a, 2009b), constitute important prerequisites for processing egomotion signals in relation to object‐motion. First, most of these regions (LOR, MT, VIP, V6, and V3A) are activated by optic flow (Smith et al., 2006; Cardin et al., 2012; Morrone et al., 2000; Wall & Smith, 2008; Pitzalis et al., 2010, 2012) which constitutes a rich source of visual cues that can facilitate navigation through the external environment. Second, MT, V3A, and VIP respond to changing heading directions (Furlan, Wann, & Smith, 2014; Huang et al., 2012), which is another important visual cue that contributes to the perception of self‐motion. Third, V6 and VIP are specialized in distinguishing among different types of self‐movement, showing a strong response to translational egomotion (Pitzalis, Sdoia, et al., 2013) that allows to extract information about the relative distance of objects, useful to act on them, or to avoid them (see also Cardin et al., 2012). Finally, area VIP (but not MT or V6) shows vestibular responses and appears to integrate visual and vestibular cues to direct self‐motion (Billington & Smith, 2015; Greenlee et al., 2016; Smith et al., 2012). Overall, some of these flow parsing‐related regions could be involved in the extraction of optic flow for the computation of heading direction (MT, V3A, and VIP), others for obstacle avoidance (V6 and VIP) and/or for visual/vestibular cue integration (VIP). Biagi, Crespi, Tosetti, and Morrone (2015) demonstrated that MT+ and V6 are operative even by 7 weeks of age, likely providing very young infants with a sense of vection. More in general, the flow parsing‐related regions could act as “sensors” of real movement in a neural network that subserves an internal, objective map of the visual field. Such an internal representation of the FOV could allow one to correctly interpret real motion as well as the plethora of sensory changes resulting from exploratory eye and self‐movements in a stable visual world (Galletti & Fattori, 2003). Of course, we are aware that with the analysis implemented here we can only identify which motion areas of the dorsal stream are likely involved in the flow‐parsing phenomenon, but without specific analysis on the timing of these activations (e.g., event‐related peak latency) we cannot assess when the activation occurred and infer directionality.

### 4.5 Ecological stimuli, eye movements, and motion perception

A peculiar aspect of this study is the choice of the visual stimuli. To reproduce a realistic self‐ and object‐motion stimulation, we used a virtual reality software simulating a train moving in a natural landscape and, more importantly, movies as motion conditions. Recent neuroimaging studies have begun to use virtual reality simulation to investigate the neural substrates of egomotion (Billington, Field, Wilkie, & Wann, 2010; Field, Wilkie, & Wann, 2007; Huang et al., 2015). Movies constitute an excellent experimental approximation to the reality and come much closer toward everyday like scenarios than clouds of dots, which are typically used (Billington & Smith, 2015; Pitzalis, Sdoia, et al., 2013; Pitzalis, Sereno, et al., 2013; Smith et al., 2006). The use of wide‐field stimuli increased the realism of the virtual environment and made the subjective experience of self‐motion (vection) particularly compelling (Palmisano et al., 2015).

To further increase the realism of our paradigm and the subjective self‐motion experience, subjects were free to move their eyes. When an individual move around the environment a coherent pattern of image motion known as optic flow reaches the retina and its center of flow (CoF) coincides with the gaze direction (Cardin et al., 2012). In this condition, the CoF indicates the direction toward which the individual is heading. In movies as those used here the CoF continuously change its position on the screen and if subjects are asked to stare at a central fixation cross, the CoF and gaze direction would dissociate through the run. Because of this, the visual percept would be unnatural, not informative about heading and not able to elicit a compelling self‐motion experience (Wann, Swapp, & Rushton, 2000). Therefore, we opted for a natural vision (free scanning). Of course, we are aware that although this is appreciable because it is exactly what happens in everyday life, the eye movements might be considered a critical confound, because part of the observed brain activity might have been induced by these eye movements. But the presence and the amount of eye movements is an ingrained feature of the type of movements we studied here. When, for instance, we move forward, we tend to keep our gaze in correspondence of the CoF. In contrast, when we observe a moving train from a still position, we tend to keep the train still on the fovea by pursuit eye movements. Also, during self‐motion consistent optic flow subjects make compensatory eye movements, and it is known that changes in such eye movements over time are correlated with reported increases in vection strength (Kim & Palmisano, 2010). These results are of course reported only when the observer freely views the self‐motion display (Palmisano, Kim, & Freeman, 2012). Without considering these different eye patterns, we feel that we are excluding important elements toward the real understanding of what really happens in the bran in the everyday life. Additionally, also previous imaging studies allowed participants to view movies reproducing self‐motion (or a combination of self/object‐motion) with no fixation point, thus better emulating the various conditions that observers would encounter in the real world (Bartels et al., 2008; Field et al., 2007). Interestingly, Field et al., (2007) replicated their motion experiment under condition of fixation in order to rule out eye movements as an explanation of the differences in brain activation. They found that just preventing actual eye movements did not prevent activation even in those cortical regions more likely influenced by eye movements, as the cortical eye fields, and the parietal eye field PEFs, which is the homolog to the area lateral intraparietal (LIP) in monkey. This is because, under conditions of fixation, movies as those used here (showing railways and natural background) were likely to produce an increase in planned but unexecuted eye movements relative to static. This is especially likely in the case of the parietal eye fields which are also involved in shifting spatial attention independently of actual eye movements (Bisley & Goldberg, 2003; Gottlieb & Goldberg, 1999). Thus, it could be argued that, in motion conditions emulating self‐motion in the real world, the impact of eye movements on brain activation cannot be completely ruled out, even when eye movements were constrained.

An additional aspect to be considered when using realistic movies reproducing different combination of self/object‐motion is the potential impact of physical motion features embedded in each single frame on the neural activity of motion‐selective regions. We found that SoM, which is a measure of the overall spread of the orientation motion within each movie, significantly influenced the activity in some motion regions, giving a positive contribution to the activation in LOR and a negative contribution to the activation in both MT+ and V6+. Since this motion parameter reflects the *SD* of motion vectors obtained in all pixels and frames within each movie, it represents an index of motion incoherence. Thus the negative relationship between SoM and the activity in both MT+ and V6+ observed in the current study is well in line with the concept that these regions are modulated by motion coherency (Smith et al., 2006; Cardin et al., 2012; Morrone et al., 2000; Wall & Smith, 2008; Pitzalis et al., 2010; 2012; Pitzalis, Bozzacchi, et al., 2013). On the other side, we did not observe significant correlations between the overall QoM and the neural activity in any of the observed motion region. At first glance it may seem to contradict results from Bartels et al. (2008) in which a significant positive correlation between variations of global motion (reflecting self‐motion) and the activity of the medial posterior parietal cortex (mPPC), which likely corresponds to the V6 area, was observed. However, their division of the total motion into a global (reflecting self‐motion) and local (reflecting object‐motion) motion components did not correspond of our motion estimates, which instead reflected the amplitude (QoM) and the direction (SoM) of motion, with only the latter likely reflecting a measure of global/coherent motion. Future studies could address the impact of the quantity and direction of motion with respect to both local and global motion variations on the neural activity of motion‐selective regions.

A final note goes to the role of vection. According to some modern theorists and researchers, our conscious experiences of self‐motion are simply intriguing epiphenomena and vection is irrelevant in simulation based self‐motion experiments (see for review Palmisano et al., 2015). According to other authors, it is possible that our conscious experiences of self‐motion play important functional roles in the perception, control, navigation, or guidance of self‐motion. For example, vection convincingly improves spatial orientation in virtual reality (Chance, Gaunet, Beall, & Loomis, 1998; Kearns, Warren, Duchon, & Tarr, 2002; Klatzky, Loomis, Beall, Chance, & Golledge, 1998; Riecke, 2008; Riecke, Feuereissen, Rieser, & McNamara, 2012). It is known that vection induces (or is based on) differential cortical activity, and many functional neuroimaging studies (including the present one) have attempted to identify the neural correlates of visual self‐motion perception (Beer, Blakemore, Previc, & Liotti, 2002; Brandt, Bucher, Seelos, & Dieterich, 1998; Cardin & Smith, 2010; de Jong, Shipp, Skidmore, Frackowiak, & Zeki, 1994; Deutschländer et al., 2004; Kleinschmidt et al., 2002; Kovács et al., 2008; Previc et al., 2000; Tokumaru, Kaida, Ashida, Yoneda, & Tatsuno, 1999; Wall et al., 2008; Wall & Smith, 2008). However, whether the BOLD signal observed in the cortical areas responding to self‐motion is correlated to the different amount of subjective experience of self‐motion is a still open question. Here, for instance, we found no sign of correlation between vection and BOLD signal even in those areas preferring self‐motion condition, suggesting that the cortical regions respond to self‐motion irrespective of vection and its subjective intensity. The lack of correlation found here seems to support the hypothesis that vection has little or no behavioral relevance. However, an alternative explanation could be that in our study we reached a ceiling effect, being the vection in the onboard condition on average high and uniformly distributed across subjects. In conclusion, the functional significance of vection is still an open and largely unexplored question (Palmisano et al., 2015) and more evidence is needed to verify if vection is or not an important parameter to be considered in future fMRI studies on the self‐motion.

### 4.6 Conclusive remarks

Many years ago, it has been suggested (Previc, 1998; Previc et al., 2000; Rosa & Tweedale, 2001) that lateral and medial motion areas are engaged in the detection of object‐ and self‐motion, respectively. In accordance with this hypothesis, we found a lateral area (MT) particularly sensitive to object‐motion and several medial motion areas (PEc, pCi, CSv, and CMA) preferring self‐motion. However, we failed to find a strict segregation of functions, since the medial motion area V6 responded equally well to both self‐ and object‐motion, and several motion areas of the dorso‐lateral surface (PIC and LOR) preferred self‐motion. In addition, some regions (V6, IPSmot, MST+, V3A/V7, LIP, SFS, and PPA) prefer not pure self‐ or object‐motion, but complex visual stimulation where both self‐ and object‐motion are present in the stimulus, similarly to what happens in daily life when people move in a dynamic and complex environment.

In macaques, information on real movement is encoded in single cells of several areas of the dorsal stream (Galletti & Fattori, 2003). Here we show that in humans the same areas, and others not yet studied in nonhuman primates, participate to the real movement recognition. We suggest that a network of cortical areas able to recognize the real motion ensures a stable and correct perception of the external visual world, necessary to orchestrate eye, arm, and body movements, while navigating in a complex and dynamic environment. We believe that this network of cortical areas is a good candidate for being the neural circuit of the flow parsing—the separation of object‐motion from self‐motion (Warren & Rushton, 2009a, 2009b).

## CONFLICT OF INTEREST

The authors declare no potential conflict of interest.

## Supporting information


**Appendix S1:** Supporting information

**Video S1** Supporting information

**Video S2** Supporting information

**Video S3** Supporting information

**Video S4** Supporting information

**Figure S1** Three regions (LOR, LIP, V3A/V7) are superimposed over the flattened left and right hemispheres of Conte69 atlas (Van Essen et al. 2011). The borders of previously identified areas (Van Essen et al. 2011; Kolster et al. 2010) are highlighted in white. The curvature is shown using light/dark gray to signify convex/concave. LH, Left Hemisphere. RH, right hemisphere. Sup. Temp., Superior Temporal sulcus.

**Figure S2** Area prostriate. **A**. Prostriate mapping by comparing any motion condition against fixation. Results are displayed on the medial and lateral folded representation of the right and left hemispheres of the template brain. The MNI coordinates (mm) and sizes (mm^3^) of the prostriate region are as follows: LH, x = −24, y = −58, z = 0, size = 79; RH, x = 24, y = −57, z = 1, size = 81. **B**. The plot for the prostriate region represents the averaged BOLD percent signal change ± standard error of the mean across subjects and hemispheres for each experimental condition: Static (Black), Offboard (Red), Onboard (Blue), Joint (Yellow) and Disjoint (Green). Significant comparisons are also reported. *p < 0.05; **p < 0.01; ***p < 0.001. Name abbreviations for some of the conditions are as follows: Sta (Static), Offb (Offboard), Onb (Onboard), Disj (Disjoint). Significant comparisons are also reported. *p < 0.05; **p < 0.01; ***p < 0.001.

## Data Availability

Present data will be made available on request in compliance with the requirements of the funding institutes, and with the institutional ethics approval.

## References

[hbm24862-bib-0001] Amiez, C. , & Petrides, M. (2014). Neuroimaging evidence of the anatomo‐functional organization of the human cingulate motor areas. Cerebral Cortex, 24(3), 563–578. 10.1093/cercor/bhs329 23131805

[hbm24862-bib-0002] Antal, A. , Baudewig, J. , Paulus, W. , & Dechent, P. (2008). The posterior cingulate cortex and planum temporale/parietal operculum are activated by coherent visual motion. Visual Neuroscience, 25(1), 17–26. 10.1017/S0952523808080024 18282307

[hbm24862-bib-0003] Arnoldussen, D. M. , Goossens, J. , & van den Ber, A. V. (2013). Differential responses in dorsal visual cortex to motion and disparity depth cues. Frontiers in Human Neuroscience, 7, 815 http://doi.org/103389/fnhum201300815 24339808 10.3389/fnhum.2013.00815 PMC3857528

[hbm24862-bib-0004] Arnoldussen, D. M. , Goossens, J. , & van den Berg, A. V. (2015). Dissociation of retinal and headcentric disparity signals in dorsal human cortex. Frontiers in Systems Neuroscience, 9, 16 http://doi.org/103389/fnsys201500016 25759642 10.3389/fnsys.2015.00016 PMC4338660

[hbm24862-bib-0005] Arnoldussen, D. M. , Goossens, J. , & van den Berg, A. V. (2011). Adjacent visual representations of self‐motion in different reference frames. Proceedings of the National Academy of Sciences of the United States of America, 108(28), 11668–11673. http://doi.org/101073/pnas1102984108 21709244 10.1073/pnas.1102984108 PMC3136271

[hbm24862-bib-0006] Astafiev, S. V. , Shulman, G. L. , Stanley, C. M. , Snyder, A. Z. , Van Essen, D. C. , & Corbetta, M. (2003). Functional organization of human intraparietal and frontal cortex for attending, looking, and pointing. The Journal of Neuroscience, 23(11), 4689–4699. 10.1523/JNEUROSCI.23-11-04689.2003 12805308 PMC6740811

[hbm24862-bib-0007] Bartels, A. , Zeki, S. , & Logothetis, N. K. (2008). Natural vision reveals regional specialization to local motion and to contrast‐invariant, global flow in the human brain. Cerebral Cortex, 18(3), 705–717. 10.1093/cercor/bhm107 17615246

[hbm24862-bib-0008] Bashkaran, V. , & Konstantinides, K. (1995). Image and video compression standards: Algorithms and architectures. Kluwer Academic Publishers 10.1007/978-1-4757-2358-8

[hbm24862-bib-0009] Battaglia‐Mayer, A. , Ferraina, S. , Genovesio, A. , Marconi, B. , Squatrito, S. , Molinari, M. , … Caminiti, R. (2001). Eye‐hand coordination during reaching. II. An analysis of the relationships between visuomanual signals in parietal cortex and parieto‐frontal association projections. Cerebral Cortex, 11, 528–544. 10.1093/cercor/11.6.528 11375914

[hbm24862-bib-0010] Beer, J. , Blakemore, C. , Previc, F. H. , & Liotti, M. (2002). Areas of the human brain activated by ambient visual motion, indicating three kinds of self‐movement. Experimental Brain Research, 143(1), 78–88. 10.1007/s00221-001-0947-y 11907693

[hbm24862-bib-0011] Ben Hamed, S. , Duhamel, J. R. , Bremmer, F. , & Graf, W. (2001). Representation of the visual field in the lateral intraparietal area of macaque monkeys: A quantitative receptive field analysis. Experimental Brain Research, 127–144(144), 140 10.1007/s002210100785 11521146

[hbm24862-bib-0012] Ben Hamed, S. , Duhamel, J. R. , Bremmer, F. , & Graf, W. (2002). Visual receptive field modulation in the lateral intraparietal area during attentive fixation and free gaze. Cereb Cortex Mar, 12(3), 234–245. 10.1093/cercor/12.3.234 11839598

[hbm24862-bib-0013] Biagi, L. , Crespi, S. A. , Tosetti, M. , & Morrone, M. C. (2015). BOLD response selective to flow‐motion in very young infants. PLoS Biology, 13(9), e1002260 10.1371/journal.pbio.1002260 26418729 PMC4587790

[hbm24862-bib-0014] Billington, J. , Field, D. T. , Wilkie, R. M. , & Wann, J. P. (2010). An fMRI study of parietal cortex involvement in the visual guidance of locomotion. Journal of Experimental Psychology. Human Perception and Performance, 36(6), 1495–1507. 10.1037/a0018728 20718562

[hbm24862-bib-0015] Billington, J. , & Smith, A. T. (2015). Neural mechanisms for discounting head‐roll‐induced retinal motion. Journal of Neuroscience, 35(12), 4851–4856. 10.1523/JNEUROSCI.3640-14.2015 25810516 PMC6705369

[hbm24862-bib-0016] Bisley, J. W. , & Goldberg, M. E. (2003). Neuronal activity in the lateral intraparietal area and spatial attention. Science, 299, 81–86. 10.1126/science.1077395 12511644

[hbm24862-bib-0017] Boccia, M. , Sulpizio, V. , Nemmi, F. , Guariglia, C. , & Galati, G. (2016). Direct and indirect parieto‐medial temporal pathways for spatial navigation in humans: Evidence from resting‐state functional connectivity. Brain Structure & Function, 222(4), 1945–1957. 10.1007/s00429-016-1318-6 27704218

[hbm24862-bib-0018] Bottini, G. , Sterzi, R. , Paulesu, E. , Vallar, G. , Cappa, S. F. , Erminio, F. , … Frackowiak, R. S. (1994). Identification of the central vestibular projections in man: A positron emission tomography activation study. Experimental Brain Research, 99, 164–169. 10.1007/BF00241421 7925790

[hbm24862-bib-0019] Braddick, O. J. , O'Brien, J. M. , Wattam‐Bell, J. , Atkinson, J. , Hartley, T. , & Turner, R. (2001). Brain areas sensitive to coherent visual motion. Perception, 30(1), 61–72. 10.1068/p3048 11257978

[hbm24862-bib-0020] Braddick, O. J. , O'Brien, J. M. , Wattam‐Bell, J. , Atkinson, J. , & Turner, R. (2000). Form and motion coherence activate independent, but not dorsal/ventral segregated, networks in the human brain. Current Biology, 10(12), 731–734. 10.1016/S0960-9822(00)00540-6 10873810

[hbm24862-bib-0021] Brandt, T. , Bucher, S. F. , Seelos, K. C. , & Dieterich, M. (1998). Bilateral functional MRI activation of the basal ganglia and middle temporal/medial superior temporal motion‐sensitive areas: Optokinetic stimulation in homonymous hemianopia. Archives of Neurology, 55(8), 1126–1131. 10.1001/archneur.55.8.1126 9708964

[hbm24862-bib-0022] Brandt, T. , Dieterich, M. , & Danek, A. (1994). Vestibular cortex lesions affect the perception of verticality. Annals of Neurology, 35, 403–412. 10.1002/ana.410350406 8154866

[hbm24862-bib-0023] Bremmer, F. , Duhamel, J. R. , Ben Hamed, S. , & Graf, W. (2002). Heading encoding in the macaque ventral intraparietal area (VIP). European Journal of Neuroscience, 16(8), 1554–1568. 10.1046/j.1460-9568.2002.02207.x 12405970

[hbm24862-bib-0024] Bremmer, F. , Schlack, A. , Shah, N. J. , Zafiris, O. , Kubischik, M. , Hoffmann, K. P. , & Fink, G. R. (2001). Polymodal motion processing in posterior parietal and premotor cortex. Neuron, 29, 287–296. 10.1016/S0896-6273(01)00198-2 11182099

[hbm24862-bib-0025] Bridgeman, B. (1973). Receptive fields in single cells of monkey visual cortex during visual tracking. The International Journal of Neuroscience, 6, 141–152. 10.3109/00207457309147657 4213353

[hbm24862-bib-0026] Britten, K. H. (2008). Mechanisms of self‐motion perception. Annual Review of Neuroscience, 31(659), 389–410. 10.1146/annurev.neuro.29.051605.112953 18558861

[hbm24862-bib-0027] Bucher, S. F. , Dieterich, M. , Wiesmann, M. , Weiss, A. , Zink, R. , Yousry, T. A. , & Brandt, T. (1998). Cerebral functional magnetic resonance imaging of vestibular, auditory, and nociceptive areas during galvanic stimulation. Annals of Neurology, 44(1), 120–125. 10.1002/ana.410440118 9667599

[hbm24862-bib-0028] Cardin, V. , Hemsworth, L. , & Smith, A. T. (2012). Adaptation to heading direction dissociates the roles of human MST and V6 in the processing of optic flow. Journal of Neurophysiology, 108(3), 794–801. 10.1152/jn.00002.2012 22592304 PMC3424094

[hbm24862-bib-0029] Cardin, V. , Sherrington, R. , Hemsworth, L. , & Smith, A. T. (2012). Human V6: Functional characterisation and localization. PLoS One, 7(10), e47685 10.1371/journal.pone.0047685 23112833 PMC3480433

[hbm24862-bib-0030] Cardin, V. , & Smith, A. T. (2010). Sensitivity of human visual and vestibular cortical regions to egomotion‐compatible visual stimulation. Cerebral Cortex, 20(8), 1964–1973. 10.1093/cercor/bhp268 20034998 PMC2901022

[hbm24862-bib-0031] Cardin, V. , & Smith, A. T. (2011). Sensitivity of human visual cortical area V6 to stereoscopic depth gradients associated with self‐motion. Journal of Neurophisiology, 106, 1240–1249. 10.1152/jn.01120.2010 PMC3174812 21653717

[hbm24862-bib-0032] Chance, S. , Gaunet, F. , Beall, A. , & Loomis, J. (1998). Locomotion mode affects the updating of objects encountered during travel: The contribution of vestibular and proprioceptive inputs to path integration. Presence (Camb), 7, 168–178. 10.1162/105474698565659

[hbm24862-bib-0033] Chen, Y. S. , Hung, Y. P. , & Fuh, C. S. (2001). Fast block matching algorithm based on the winner‐update strategy. IEEE Transactions on Image Processing, 10(8), 1212–1222. 10.1109/83.935037 18255538

[hbm24862-bib-0034] Chen, Z. (2009). Efficient block matching algorithm for motion estimation. Int J sig Process, 5(2), 133–137. 10.1109/SPCOM.2004.1458406

[hbm24862-bib-0035] Colby, C. L. , Duhamel, J. R. , & Goldberg, M. E. (1993). Ventral intraparietal area of the macaque: Anatomic location and visual response properties. Journal of Neurophysiology, 69(3), 902–914. 10.1152/jn.1993.69.3.902 8385201

[hbm24862-bib-0036] Corbetta, M. , Miezin, F. M. , Shulman, G. L. , & Petersen, S. E. (1993). A PET study of visuospatial attention. The Journal of Neuroscience: The Official Journal of the Society for Neuroscience, 13(3), 1202–1226. Retrieved from. http://www.ncbi.nlm.nih.gov/pubmed/8441008 8441008 10.1523/JNEUROSCI.13-03-01202.1993 PMC6576604

[hbm24862-bib-0037] Corbetta, M. , Akbudak, E. , Conturo, T. E. , Snyder, A. Z. , Ollinger, J. M. , Drury, H. A. , … Shulman, G. L. (1998). A common network of functional areas for attention and eye movements. Neuron, 21(4), 761–773. 10.1016/S0896-6273(00)80593-0 9808463

[hbm24862-bib-0038] Culham, J. C. , Brandt, S. A. , Cavanagh, P. , Kanwisher, N. G. , Dale, A. M. , & Tootell, R. B. (1998). Cortical fMRI activation produced by attentive tracking of moving targets. Journal of Neurophysiology, 80(5), 2657–2670. 10.1152/jn.1998.80.5.2657 9819271

[hbm24862-bib-0039] de Jong, B. M. , Shipp, S. , Skidmore, B. , Frackowiak, R. S. , & Zeki, S. (1994). The cerebral activity related to the visual perception of forward motion in depth. Brain, 117, 1039–1054. 10.1093/brain/117.5.1039 7953587

[hbm24862-bib-0040] Deutschländer, A. , Bense, S. , Stephan, T. , Schwaiger, M. , Dieterich, M. , & Brandt, T. (2004). Rollvection versus linearvection: Comparison of brain activations in PET. Human Brain Mapping, 21(3), 143–153. 10.1002/hbm.10155 14755834 PMC6871853

[hbm24862-bib-0041] Dieterich, M. , Bense, S. , Lutz, S. , Drzezga, A. , Stephan, T. , Bartenstein, P. , & Brandt, T. (2003). Dominance for vestibular cortical function in the non‐dominant hemisphere. Cerebral Cortex, 13, 994–1007. 10.1093/cercor/13.9.994 12902399

[hbm24862-bib-0042] Dieterich, M. , Bucher, S. F. , Seelos, K. C. , & Brandt, T. (1998). Horizontal or vertical optokinetic stimulation activates visual motion‐sensitive, ocular motor and vestibular cortex areas with right hemispheric dominance. An fMRI study. Brain, 121, 1479–1495. 10.1093/brain/121.8.1479 9712010

[hbm24862-bib-0043] Duffy, C. J. (1998). MST neurons respond to optic flow and translational movement. Journal of Neurophysiology, 80(4), 1816–1827. 10.1016/S0042-6989(97)00346-5 9772241

[hbm24862-bib-0044] Duffy, C. J. , & Wurtz, R. H. (1991). Sensitivity of MST neurons to optic flow stimuli I A continuum of response selectivity to large‐field stimuli. Journal of Neurophysiology, 65(6), 1329–1345. 10.1152/jn.1991.65.6.1329 1875243

[hbm24862-bib-0045] Duhamel, J. R. , Colby, C. L. , & Goldberg, M. E. (1998). Ventral intraparietal area of the macaque: Congruent visual and somatic response properties. Journal of Neurophysiology, 79(1), 126–136. 10.1234/12345678 9425183

[hbm24862-bib-0046] Dukelow, S. P. , DeSouza, J. F. X. , Culham, J. C. , van den Berg, A. V. , Menon, R. S. , & Vilis, T. (2001). Distinguishing subregions of the human MT+ complex using visual fields and pursuit eye movements. Journal of Neurophysiology, 86(4), 1991–2000. 11600656 10.1152/jn.2001.86.4.1991

[hbm24862-bib-0047] Dupont, P. , De Bruyn, B. , Vandenberghe, R. , Rosier, A. M. , Michiels, J. , Marchal, G. , … Orban, G. A. (1997). The kinetic occipital region in human visual cortex. Cerebral Cortex, 7(3), 283–292. 9143447 10.1093/cercor/7.3.283

[hbm24862-bib-0048] Eickhoff, S. B. , Amunts, K. , Mohlberg, H. , & Zilles, K. (2006). The human parietal operculum. II. Stereotaxic maps and correlation with functional imaging results. Cerebral Cortex, 16(2), 268–279. 10.1093/cercor/bhi106 15888606

[hbm24862-bib-0049] Eifuku, S. , & Wurtz, R. H. (1998). Response to motion in extrastriate area MSTl: Center‐surround interactions. Journal of Neurophysiology, 80(1), 282–296. 10.1152/jn.1998.80.1.282 9658050

[hbm24862-bib-0050] Epstein, R. , & Kanwisher, N. (1998). A cortical representation of the local visual environment. Nature, 392(6676), 598–601. 10.1038/33402 9560155

[hbm24862-bib-0051] Epstein, R. A. (2008). Parahippocampal and retrosplenial contributions to human spatial navigation. Trends in Cognitive Sciences, 12(10), 388–396. 10.1016/j.tics.2008.07.004 18760955 PMC2858632

[hbm24862-bib-0052] Erickson, R. G. , & Thier, P. (1991). A neuronal correlate of spatial stability during periods of self‐induced visual motion. Experimental Brain Research, 86, 608–616. 10.1007/BF00230534 1761094

[hbm24862-bib-0053] Fasold, O. , von Brevern, M. , Kuhberg, M. , Ploner, C. J. , Villringer, A. , Lempert, T. , & Wenzel, R. (2002). Human vestibular cortex as identified with caloric stimulation in functional magnetic resonance imaging. NeuroImage, 17(3), 1384–1393. 10.1006/nimg.2002.1241 12414278

[hbm24862-bib-0054] Fattori, P. , Pitzalis, S. , & Galletti, C. (2009). The cortical visual area V6 in macaque and human brains. Journal of Physiology, Paris, 103, 88–97. 10.1016/j.jphysparis.2009.05.012 19523515

[hbm24862-bib-0055] Field, D. T. , Wilkie, R. M. , & Wann, J. P. (2007). Neural systems in the visual control of steering. The Journal of Neuroscience, 27(30), 8002–8010. 10.1523/JNEUROSCI.2130-07.2007 17652590 PMC6672731

[hbm24862-bib-0056] Field, D. T. , Inman, L. A. , & Li, L. (2015). Visual processing of optic flow and motor control in the human posterior cingulate sulcus. Cortex, 71, 377–389. 10.1016/j.cortex.2015.07.014 26318342

[hbm24862-bib-0057] Fillimon, F. , Rieth, C. A. , Sereno, M. I. , & Cottrell, G. W. (2015). Observed, executed, and imagined action representations can be decoded from ventral and dorsal areas. Cerebral Cortex, 25(9), 3144–3158. 10.1093/cercor/bhu110 24862848

[hbm24862-bib-0058] Fischer, E. , Bülthoff, H. H. , Logothetis, N. K. , & Bartels, A. (2012). Visual motion responses in the posterior cingulate sulcus: A comparison to V5/MT and MST. Cerebral Cortex, 22(4), 865–876. 10.1093/cercor/bhr154 21709176 PMC3306574

[hbm24862-bib-0059] Frank, S. M. , & Greenlee, M. W. (2014). An MRI‐compatible caloric stimulation device for the investigation of human vestibular cortex. Journal of Neuroscience Methods, 235, 208–218. 10.1016/j.jneumeth.2014.07.008 25064191

[hbm24862-bib-0060] Frank, S. M. , Baumann, O. , Mattingley, J. B. , & Greenlee, M. W. (2014). Vestibular and visual responses in human posterior insular cortex. Journal of Neurophysiology, 112(10), 2481–2491. 10.1152/jn.00078.2014 25185806

[hbm24862-bib-0061] Frank, S. M. , Sun, L. , Forster, L. , Peter, U. T. , & Greenlee, M. W. (2016). Cross‐modal attention effects in vestibular cortex during attentive tracking of moving objects. The Journal of Neuroscience, 36(50), 12720–12728. 10.1523/JNEUROSCI.2480-16.2016 27821579 PMC6705664

[hbm24862-bib-0062] Frank, S. M. , Wirth, A. M. , & Greenlee, M. W. (2016). Visual‐vestibular processing in the human Sylvian fissure. Journal of Neurophysiology, 116(2), 263–271. 10.1152/jn.00009.2016 27075535 PMC4969386

[hbm24862-bib-0063] Frenz, H. , Bremmer, F. , & Lappe, M. (2003). Discrimination of travel distances from ‘situated’ optic flow. Vision Research, 43, 2173–2183. 10.1016/S0042-6989(03)00337-7 12855252

[hbm24862-bib-0064] Friberg, L. , Olsen, T. S. , Roland, P. E. , Paulson, O. B. , & Lassen, N. A. (1985). Focal increase of blood flow in the cerebral cortex of man during vestibular stimulation. Brain, 108, 609–623. 10.1093/brain/108.3.609 3876134

[hbm24862-bib-0065] Furlan, M. , Wann, J. P. , & Smith, A. T. (2014). A representation of changing heading direction in human cortical areas pVIP and CSv. Cerebral Cortex, 24(11), 2848–2858. 10.1093/cercor/bht132 23709643

[hbm24862-bib-0066] Galati, G. , Committeri, G. , Pitzalis, S. , Pelle, G. , Patria, F. , Fattori, P. , & Galletti, C. (2011). Intentional signals during saccadic and reaching delays in the human posterior parietal cortex. The European Journal of Neuroscience, 34, 1871–1885. 10.1111/j.1460-9568.2011.07885.x 22017280

[hbm24862-bib-0067] Gallant, M. , Cote, G. , & Kossentini, F. (1999). An efficient computation‐constrained block‐based motion estimation algorithm for low bit rate video coding. IEEE Transactions on Image Processing, 8(12), 1816–1823. 10.1109/83.806627 18267458

[hbm24862-bib-0068] Galletti, C. , & Fattori, P. (2018). The dorsal visual stream revisited: Stable circuits or dynamic pathways? Cortex, 98, 203–217. 10.1016/j.cortex.2017.01.009 28196647

[hbm24862-bib-0069] Galletti, C. , Battaglini, P. P. , & Aicardi, G. (1988). ‘Real‐motion’ cells in visual area V2 of behaving macaque monkeys. Experimental Brain Research, 69(2), 279–288. 10.1007/BF00230838 3345807

[hbm24862-bib-0070] Galletti, C. , Squatrito, S. , Battaglini, P. P. , & Grazia Maioli, M. (1984). ‘Real‐motion’ cells in the primary visual cortex of macaque monkeys. Brain Research, 301(1), 95–110. 10.1016/0006-8993(84)90406-2 6733490

[hbm24862-bib-0071] Galletti, C. , Battaglini, P. P. , & Fattori, P. (1990). “Real‐motion” cells in area V3A of macaque visual cortex. Experimental Brain Research, 82(1), 67–76. 10.1007/BF00230838 2257915

[hbm24862-bib-0072] Galletti, C. , & Fattori, P. (2003). Neuronal mechanisms for detection of motion in the field of view. Neuropsychologia, 41(13), 1717–1727. 10.1016/S0028-3932(03)00174-X 14527536

[hbm24862-bib-0600] Galletti, C. , Gamberini, M. , Kutz, D.F. , Fattori, P., Luppino, G. , Matelli, M. (2001). The cortical connections of area V6: an occipito‐parietal network processing visual information. European Journal of Neuroscience, 13, 1572–1588. 11328351 10.1046/j.0953-816x.2001.01538.x

[hbm24862-bib-0073] Georgieva, S. S. , Todd, J. T. , Peeters, R. , & Orban, G. A. (2008). The extraction of 3D shape from texture and shading in the human brain. Cerebral Cortex, 18(10), 2416–2438. 10.1093/cercor/bhn002 18281304 PMC2536698

[hbm24862-bib-0074] Gitelman, D. R. , Nobre, A. C. , Parrish, T. B. , LaBar, K. S. , Kim, Y.‐H. , Meyer, J. R. , & Mesulam, M.‐M. (1999). A large‐scale distributed network for covert spatial attention: Further anatomical delineation based on stringent behavioral and cognitive controls. Brain, 122, 1093–1106. 10.1093/brain/122.6.1093 10356062

[hbm24862-bib-0075] Gottlieb, J. , & Goldberg, M. E. (1999). Activity of neurons in the lateral intraparietal area of the monkey during an antisaccade task. Nature Neuroscience, 2, 906–912. 10.1038/13209 10491612

[hbm24862-bib-0076] Graziano, M. S. , Andersen, R. A. , & Snowden, R. J. (1994). Tuning of MST neurons to spiral motions. The Journal of Neuroscience, 14(1), 54–67. 10.1523/JNEUROSCI.14-01-00054.1994 8283251 PMC6576843

[hbm24862-bib-0077] Greenlee, M. W. , Frank, S. M. , Kaliuzhna, M. , Blanke, O. , Bremmer, F. , Churan, J. , … Smith, A. T. (2016). Multisensory integration in self motion perception. Multisensory Research, 29(6–7), 525–556. 10.1163/22134808-00002527

[hbm24862-bib-0078] Grossberg, S. , Mingolla, E. , & Pack, C. (1999). A neural model of motion processing and visual navigation by cortical area MST. Cerebral Cortex, 9(8), 878–895. 10.1093/cercor/9.8.878 10601006

[hbm24862-bib-0079] Grüsser, O. J. , Pause, M. , & Schreiter, U. (1990). Vestibular neurones in the parieto‐insular cortex of monkeys (*Macaca fascicularis*): Visual and neck receptor responses. The Journal of Physiology, 430, 559–583. 10.1113/jphysiol.1990.sp018307 2086774 PMC1181753

[hbm24862-bib-0080] Guldin, W. O. , & Grüsser, O. J. (1998). Is there a vestibular cortex? Trends in Neurosciences, 21(6), 254–259. 10.1016/S0166-2236(97)01211-3 9641538

[hbm24862-bib-0081] Hagler, D. J., Jr. , Riecke, L. , & Sereno, M. I. (2007). Parietal and superior frontal visuospatial maps activated by pointing and saccades. NeuroImage, 35, 1562–1577. 10.1016/j.neuroimage.2007.01.033 17376706 PMC2752728

[hbm24862-bib-0082] Helfrich, R. F. , Becker, H. G. , & Haarmeier, T. (2013). Processing of coherent visual motion in topographically organized visual areas in human cerebral cortex. Brain Topography, 26(2), 247–263. 10.1007/s10548-012-0226-1 22526896

[hbm24862-bib-0083] Huang, R. S. , Chen, C. F. , & Sereno, M. I. (2015). Neural substrates underlying the passive observation and active control of translational egomotion. The Journal of Neuroscience, 35(10), 4258–4267. 10.1523/JNEUROSCI.2647-14.2015 25762672 PMC4355198

[hbm24862-bib-0084] Huang, R. S. , & Sereno, M. I. (2013). Bottom‐up retinotopic organization supports top‐down mental imagery. The Open Neuroimaging Journal, 7(858), 58–67. 10.2174/1874440001307010058 24478813 PMC3905356

[hbm24862-bib-0085] Huang, R. S. , Chen, C. , Tran, A. T. , Holstein, K. L. , & Sereno, M. I. (2012). Mapping multisensory parietal face and body areas in humans. Proceedings of the National Academy of Sciences of the United States of America, 109(44), 18114–18119. 10.1073/pnas.1207946109 23071340 PMC3497759

[hbm24862-bib-0086] Hui, K. C. , & Siu, W. C. (2007). Extended analysis of motion‐compensated frame difference for block‐based motion prediction error. IEEE Transactions on Image Processing, 16(5), 1232–1245. 10.1109/TIP.2007.894263 17491455

[hbm24862-bib-0087] Huk, A. C. , Dougherty, R. F. , & Heeger, D. J. (2002). Retinotopy and functional subdivision of human areas MT and MST. The Journal of Neuroscience, 22, 7195–7205. 10.1523/JNEUROSCI.22-16-07195.2002 12177214 PMC6757870

[hbm24862-bib-0088] Indovina, I. , Maffei, V. , Bosco, G. , Zago, M. , Macaluso, E. , & Lacquaniti, F. (2005). Representation of visual gravitational motion in the human vestibular cortex. Science, 308(5720), 416–419. 10.1126/science.1107961 15831760

[hbm24862-bib-0089] Jain, J. R. , & Jain, A. K. (1981). Displacement measurement and its application in interframe image coding. IEEE Transactions on Communications, 29, 1799–1808. 10.1109/TCOM.1981.1094950

[hbm24862-bib-0090] Kearns, M. , Warren, W. , Duchon, A. , & Tarr, M. (2002). Path integration from optic flow and body senses in a homing task. Perception, 31, 349–374. 10.1068/p3311 11954696

[hbm24862-bib-0091] Kim, Y. H. , Gitelman, D. R. , Nobre, A. C. , Parrish, T. B. , LaBar, K. S. , & Mesulam, M. M. (1999). The large‐scale neural network for spatial attention displays multifunctional overlap but differential asymmetry. NeuroImage, 9(3), 269–277. 10.1006/nimg.1999.0408 10075897

[hbm24862-bib-0092] Kim, J. , & Palmisano, S. (2010). Eccentric gaze dynamics enhance vection in depth. Journal of Vision, 10(12), 1–11. 10.1167/10.12.7 21047739

[hbm24862-bib-0093] Klatzky, R. L. , Loomis, J. M. , Beall, A. C. , Chance, S. S. , & Golledge, R. G. (1998). Spatial updating of self‐position and orientation during real, imagined, and virtual locomotion. Psychological Science, 9, 293–298. 10.1111/1467-9280.00058

[hbm24862-bib-0094] Kleinschmidt, A. , Thilo, K. V. , Büchel, C. , Gresty, M. A. , Bronstein, A. M. , & Frackowiak, R. S. (2002). Neural correlates of visual‐motion perception as object‐ or self‐motion. NeuroImage, 16(4), 873–882. 10.1006/nimg.2002.1181 12202076

[hbm24862-bib-0095] Koenderink, J. J. , & Physics, P. (1986). Optic flow. Vision Research, 26(1), 161–179. 10.1016/0042-6989(86)90078-7 3716209

[hbm24862-bib-0096] Kolster, H. , Peeters, R. , & Orban, G. A. (2010). The retinotopic organization of the human middle temporal area MT/V5 and its cortical neighbors. Journal of Neuroscience, 30(29), 9801–9820. 10.1523/JNEUROSCI.2069-10.2010 20660263 PMC6632824

[hbm24862-bib-0097] Korkmaz Hacialihafiz, D. , & Bartels, A. (2015). Motion responses in scene‐selective regions. NeuroImage, 118, 438–444. 10.1016/j.neuroimage.2015.06.031 26091852

[hbm24862-bib-0098] Kovács, G. , Raabe, M. , & Greenlee, M. W. (2008). Neural correlates of visually induced self‐motion illusion in depth. Cerebral Cortex, 18, 1779–1787. 10.1093/cercor/bhm203 18063566

[hbm24862-bib-0099] Koyama, M. , Hasegawa, I. , Osada, T. , Adachi, Y. , Nakahara, K. , & Miyashita, Y. (2004). Functional magnetic resonance imaging of macaque monkeys performing visually guided saccade tasks: Comparison of cortical eye fields with humans. Neuron, 41(5), 795–807. 10.1016/S0896-6273(04)00047-9 15003178

[hbm24862-bib-0100] Kriegeskorte, N. , Simmons, W. K. , Bellgowan, P. S. , & Baker, C. I. (2009). Circular analysis in systems neuroscience: The dangers of double dipping. Nature Neuroscience, 12(5), 535–540. 10.1038/nn.2303 19396166 PMC2841687

[hbm24862-bib-0101] Kubanek, J. , Li, J. M. , & Snyder, L. H. (2015). Motor role of parietal cortex in a monkey model of hemispatial neglect. Proceedings of the National Academy of Sciences of the United States of America, 112(16), E2067–E2072. 10.1073/pnas.1418324112 25759438 PMC4413322

[hbm24862-bib-0102] Kwong, K. K. , Belliveau, J. W. , Chesler, D. A. , Goldberg, I. E. , Weisskoff, R. M. , Poncelet, B. P. , & Turner, R. (1992). Dynamic magnetic resonance imaging of human brain activity during primary sensory stimulation. Proceedings of the National Academy of Sciences of the United States of America, 89(12), 5675–5679. 10.1073/pnas.89.12.5675 1608978 PMC49355

[hbm24862-bib-0103] Lancaster, J. L. , Woldorff, M. G. , Parsons, L. M. , Liotti, M. , Freitas, C. S. , Rainey, L. , … Fox, P. T. (2000). Automated Talairach atlas labels for functional brain mapping. Human Brain Mapping, 10(3), 120–131. 10.1002/1097-0193(200007)10:33.0.CO;2-8 10912591 PMC6871915

[hbm24862-bib-0104] Larsson, J. , & Heeger, D. J. (2006). Two retinotopic visual areas in human lateral occipital cortex. The Journal of Neuroscience, 26(51), 13128–13142. 10.1523/JNEUROSCI.1657-06.2006 17182764 PMC1904390

[hbm24862-bib-0105] Mazziotta, J. C. , Toga, A. W. , Evans, A. , Fox, P. , & Lancaster, J. (1995). A probabilistic atlas of the human brain: Theory and rationale for its development. The International Consortium for Brain Mapping (ICBM). NeuroImage, 2, 89–101. 10.1006/nimg.1995.1012 9343592

[hbm24862-bib-0106] Medendorp, W. P. , Tweed, D. B. , & Crawford, J. D. (2003). Motion parallax is computed in the updating of human spatial memory. The Journal of Neuroscience, 23, 8135–8142. 10.1523/JNEUROSCI.23-22-08135.2003 12954876 PMC6740481

[hbm24862-bib-0107] Mesulam, M. M. (1999). Spatial attention and neglect: Parietal, frontal and cingulate contributions to the mental representation and attentional targeting of salient extrapersonal events. Philosophical Transactions of the Royal Society B: Biological Sciences, 354(1387), 1325–1346. 10.1098/rstb.1999.0482 PMC1692628 10466154

[hbm24862-bib-0108] Mikellidou, K. , Kurzawski, J. W. , Frijia, F. , Montanaro, D. , Greco, V. , Burr, D. C. , & Morrone, M. C. (2017). Area prostriata in the human brain. Current Biology, 27(19), 3056–3060.e3. 10.1016/j.cub.2017.08.065 28966090

[hbm24862-bib-0109] Morrone, M. C. , Tosetti, M. , Montanaro, D. , Fiorentini, A. , Cioni, G. , & Burr, D. C. (2000). A cortical area that responds specifically to optic flow, revealed by fMRI. Nature Neuroscience, 3(12), 1322–1328. 10.1038/81860 11100154

[hbm24862-bib-0110] Moshe, Y. , & Hel‐Or, H. (2009). Video block motion estimation based on gray‐code kernels. IEEE Transactions on Image Processing, 18(10), 2243–2254. 10.1109/TIP.2009.2025559 19535322

[hbm24862-bib-0111] Moutoussis, K. , Keliris, G. , Kourtzi, Z. , & Logothetis, N. (2005). A binocular rivalry study of motion perception in the human brain. Vision Research, 45(17), 2231–2243. 10.1016/j.visres.2005.02.007 15924938

[hbm24862-bib-0112] Nelissen, K. , Vanduffel, W. , & Orban, G. A. (2006). Charting the lower superior temporal region, a new motion‐sensitive region in monkey superior temporal sulcus. The Journal of Neuroscience, 26(22), 5929–5947. 10.1523/JNEUROSCI.0824-06.2006 16738235 PMC6675228

[hbm24862-bib-0113] Nie, Y. , & Ma, K. K. (2002). Adaptive rood pattern search for fast block‐matching motion estimation. IEEE Transactions on Image Processing, 11(12), 1442–1449. 10.1109/TIP.2002.806251 18249712

[hbm24862-bib-0114] Orban, G. A. , Lagae, L. , Verri, A. , Raiguel, S. , Xiao, D. , Maes, H. , & Torre, V. (1992). First‐order analysis of optical flow in monkey brain. Proceedings of the National Academy of Sciences of the United States of America, 89(7), 2595–2599. 10.1073/pnas.89.7.2595 1557363 PMC48708

[hbm24862-bib-0115] Orban, G. A. , Sunaert, S. , Todd, J. T. , Van Hecke, P. , & Marchal, G. (1999). Human cortical regions involved in extracting depth from motion. Neuron, 24(4), 929–940. 10.1016/S0896-6273(00)81040-5 10624956

[hbm24862-bib-0116] Palmisano, S. , Allison, R. S. , Schira, M. M. , & Barry, R. J. (2015). Future challenges for vection research: Definitions, functional significance, measures, and neural bases. Frontiers in Psychology, 6(193), 1–15. 10.3389/fpsyg.2015.00193 25774143 PMC4342884

[hbm24862-bib-0117] Palmisano, S. , Kim, J. , & Freeman, T. C. A. (2012). Horizontal fixation point oscillation and simulated viewpoint oscillation both increase vection in depth. Journal of Vision, 12(12), 15 10.1167/12.12.15 23184234

[hbm24862-bib-0118] Perry, R. J. , & Zeki, S. (2000). The neurology of saccades and covert shifts in spatial attention: An event‐related fMRI study. Brain, 123(Pt 11), 2273–2288. 10.1093/brain/123.11.2273 11050027

[hbm24862-bib-0119] Petit, L. , & Haxby, J. V. (1999). Functional anatomy of pursuit eye movements in humans as revealed by fMRI. Journal of Neurophysiology, 82(1), 463–471. 10.1152/jn.1999.82.1.463 10400972

[hbm24862-bib-0120] Peuskens, H. , Sunaert, S. , Dupont, P. , Van Hecke, P. , & Orban, G. A. (2001). Human brain regions involved in heading estimation. The Journal of Neuroscience, 21(7), 2451–2461. 10.1523/JNEUROSCI.21-07-02451.2001 11264319 PMC6762416

[hbm24862-bib-0121] Picard, N. , & Strick, P. L. (1996). Motor areas of the medial wall: A review of their location and functional activation. Cerebral Cortex, 6(3), 342–353. 10.1093/cercor/6.3.342 8670662

[hbm24862-bib-0122] Pitzalis, S. , Fattori, P. , & Galletti, C. (2015). The human cortical areas V6 and V6A. Visual Neuroscience, 32, E007 10.1017/S0952523815000048 26241369

[hbm24862-bib-0123] Pitzalis, S. , Serra, C. , Sulpizio, V. , Di Marco, S. , Fattori, P. , Galati, G. , & Galletti, C. (2019). A putative human homologue of the macaque area PEc. NeuroImage, 202, 1–18. 10.1016/j.neuroimage.2019.116092 31408715

[hbm24862-bib-0124] Pitzalis, S. , Bozzacchi, C. , Bultrini, A. , Fattori, P. , Galletti, C. , & Di Russo, F. (2013). Parallel motion signals to the medial and lateral motion areas V6 and MT+. NeuroImage, 67, 89–100. 10.1016/j.neuroimage.2012.11.022 23186916

[hbm24862-bib-0125] Pitzalis, S. , Fattori, P. , & Galletti, C. (2013). The functional role of the medial motion area V6. Frontiers in Behavioral Neuroscience, 6(91), 1–13. 10.3389/fnbeh.2012.00091 PMC3546310 23335889

[hbm24862-bib-0126] Pitzalis, S. , Galletti, C. , Huang, R. S. , Patria, F. , Committeri, G. , Galati, G. , & Sereno, M. I. (2006). Wide‐field retinotopy defines human cortical visual area V6. Journal of Neuroscience, 26(30), 7962–7973. 10.1523/JNEUROSCI.0178-06.2006 16870741 PMC6674231

[hbm24862-bib-0127] Pitzalis, S. , Sdoia, S. , Bultrini, A. , Committeri, G. , Di Russo, F. , Fattori, P. , & Galati, G. (2013). Selectivity to translational egomotion in human brain motion areas. PLoS One, 8(4), 1–14. 10.1371/journal.pone.0060241 PMC3618224 23577096

[hbm24862-bib-0128] Pitzalis, S. , Sereno, M. I. , Committeri, G. , Fattori, P. , Galati, G. , Patria, F. , & Galletti, C. (2010). Human V6: The medial motion area. Cerebral Cortex, 20(2), 411–424. 10.1093/cercor/bhp112 19502476 PMC2803738

[hbm24862-bib-0129] Pitzalis, S. , Sereno, M. I. , Committeri, G. , Fattori, P. , Galati, G. , Tosoni, a. , & Galletti, C. (2013). The human homologue of macaque area V6A. NeuroImage, 82, 517–530. 10.1016/j.neuroimage.2013.06.026 23770406 PMC3760586

[hbm24862-bib-0130] Pitzalis, S. , Strappini, F. , De Gasperis, M. , Bultrini, A. , & Di Russo, F. (2012). Spatio‐temporal brain mapping of motion‐onset VEPs combined with fMRI and retinotopic maps. PLoS One, 7(4), e35771 10.1371/journal.pone.0035771 22558222 PMC3338463

[hbm24862-bib-0131] Previc, F. H. (1998). The neuropsychology of 3‐D space. Psychological Bulletin, 124(2), 123–164. 10.1037/0033-2909.124.2.123 9747184

[hbm24862-bib-0132] Previc, F. H. , Liotti, M. , Blakemore, C. , Beer, J. , & Fox, P. (2000). Functional imaging of brain areas involved in the processing of coherent and incoherent wide field‐of‐view visual motion. Experimental Brain Research, 131(4), 393–405. 10.1007/s002219900298 10803409

[hbm24862-bib-0133] Quinlan, D. J. , & Culham, J. C. (2007). fMRI reveals a preference for near viewing in the human parieto‐occipital cortex. NeuroImage, 36, 167–187. 10.1016/j.neuroimage.2007.02.029 17398117

[hbm24862-bib-0134] Raffi, M. , Carrozzini, C. , Maioli, M. G. , & Squatrito, S. (2010). Multimodal representation of optic flow in area PEc of macaque monkey. Neuroscience, 171, 1241–1255. 10.1016/j.neuroscience.2010.09.026 20870015

[hbm24862-bib-0135] Raffi, M. , Squatrito, S. , & Maioli, M. G. (2002). Neuronal responses to optic flow in the monkey parietal area PEc. Cerebral Cortex, 12, 639–646. 10.1093/cercor/12.6.639 12003863

[hbm24862-bib-0700] Riecke, B.E. (2008). Consistent Left‐Right Reversals for Visual Path Integration in Virtual Reality: More Than a Failure to Update One's Heading?. Presence: Teleoperators and Virtual Environments. 17, 143–175. 10.1162/pres.17.2.143

[hbm24862-bib-0136] Riecke, B. , Feuereissen, D. , Rieser, J. J. , & McNamara, T. P. (2012). Self‐motion illusions (Vection) in VR are they good for anything? IEEE Conference on Virtual Reality, 35–38. 10.1109/VR.2012.6180875

[hbm24862-bib-0137] Rosa, M. G. , & Tweedale, R. (2001). The dorsomedial visual areas in New World and Old World monkeys: Homology and function. The European Journal of Neuroscience, 13(3), 421–427. 10.1046/j.0953-816X.2000.01414.x 11168549

[hbm24862-bib-0138] Saito, H. , Yukie, M. , Tanaka, K. , Hikosaka, K. , Fukada, Y. , & Iwai, E. (1986). Integration of direction signals of image motion in the superior temporal sulcus of the macaque monkey. The Journal of Neuroscience, 6(1), 145–157. 10.1007/BF00248524 3944616 PMC6568620

[hbm24862-bib-0139] Sakata, H. , Shibutan, H. , Kawano, K. , & Harrington, T. L. (1985). Neural mechanisms of space vision in the parietal association cortex of the monkey. Vision Research, 25, 453–463. 10.1016/0042-6989(85)90070-7 4024464

[hbm24862-bib-0140] Schindler, A. , & Bartels, A. (2016). Motion parallax links visual motion areas and scene regions. NeuroImage, 125, 803–812. 10.1016/j.neuroimage.2015.10.066 26515906

[hbm24862-bib-0141] Schluppeck, D. , Curtis, C. E. , Glimcher, P. W. , & Heeger, D. J. (2006). Sustained activity in topographic areas of human posterior parietal cortex during memory‐guided saccades. The Journal of Neuroscience, 26(19), 5098–5108. 10.1523/JNEUROSCI.5330-05.2006 16687501 PMC1538982

[hbm24862-bib-0142] Schluppeck, D. , Glimcher, P. , & Heeger, D. J. (2005). Topographic organization for delayed saccades in human posterior parietal cortex. Journal of Neurophysiology, 94(2), 1372–1384. 10.1152/jn.01290.2004 15817644 PMC2367322

[hbm24862-bib-0143] Sereno, M. I. , Dale, A. M. , Reppas, J. B. , Kwong, K. K. , Belliveau, J. W. , Brady, T. J. , … Tootell, R. B. H. (1995). Borders of multiple visual areas in humans revealed by functional magnetic resonance imaging. Science, 268, 889–893. 10.1126/science.775437 7754376

[hbm24862-bib-0144] Sereno, M. I. , & Huang, R. S. (2006). A human parietal face area contains aligned head‐centered visual and tactile maps. Nature Neuroscience, 9(10), 1337–1343. 10.1038/nn1777 16998482

[hbm24862-bib-0145] Sereno, M. I. , Pitzalis, S. , & Martinez, A. (2001). Mapping of contralateral space in retinotopic coordinates by a parietal cortical area in humans. Science, 294(5545), 1350–1354. 10.1126/science.1063695 11701930

[hbm24862-bib-0146] Serra, C. , Galletti, C. , Di Marco, S. , Fattori, P. , Galati, G. , Sulpizio, V. , & Pitzalis, S. (2019). Egomotion‐related visual areas respond to active leg movements. Human Brain Mapping, 40, 3174–3191. 10.1002/hbm.24589 30924264 PMC6865514

[hbm24862-bib-0147] Sherrill, K. R. , Chrastil, E. R. , Ross, R. S. , Erdem, U. M. , Hasselmo, M. E. , & Stern, C. E. (2015). Functional connections between optic flow areas and navigationally responsive brain regions during goal‐directed navigation. NeuroImage, 118, 386–396. 10.1016/j.neuroimage.2015.06.009 26054874 PMC9441296

[hbm24862-bib-0148] Silson, E. H. , Steel, A. D. , & Baker, C. I. (2016). Scene‐selectivity and retinotopy in medial parietal cortex. Frontiers in Human Neuroscience, 10(412), 1–17. 10.3389/fnhum.2016.00412 27588001 PMC4988988

[hbm24862-bib-0149] Silver, M. A. , Ress, D. , & Heeger, D. J. (2005). Topographic maps of visual spatial attention in human parietal cortex. Journal of Neurophysiology, 94, 1358–1371. 10.1152/jn.01316.2004 15817643 PMC2367310

[hbm24862-bib-0150] Smith, A. T. , Greenlee, M. W. , Singh, K. D. , Kraemer, F. M. , & Hennig, J. (1998). The processing of first‐ and second‐order motion in human visual cortex assessed by functional magnetic resonance imaging (fMRI). The Journal of Neuroscience, 18, 3816–3830. 10.1523/JNEUROSCI.18-10-03816.1998 9570811 PMC6793149

[hbm24862-bib-0151] Smith, A. T. , Wall, M. B. , & Thilo, K. V. (2012). Vestibular inputs to human motion‐sensitive visual cortex. Cerebral Cortex, 22(5), 1068–1077. 10.1093/cercor/bhr179 21743097

[hbm24862-bib-0152] Smith, A. T. , Wall, M. B. , Williams, A. L. , & Singh, K. D. (2006). Sensitivity to optic flow in human cortical areas MT and MST. European Journal of Neuroscience, 23(2), 561–569. 10.1111/j.1460-9568.2005.04526.x 16420463

[hbm24862-bib-0153] Strappini, F. , Gilboa, E. , Pitzalis, S. , Kay, K. , McAvoy, M. , Nehorai, A. , & Snyder, A. Z. (2017). Adaptive smoothing based on Gaussian processes regression increases the sensitivity and specificity of fMRI data. Human Brain Mapping, 38(3), 1438–1459. 10.1002/hbm.23464 27943516 PMC6867115

[hbm24862-bib-0154] Strappini, F. , Pitzalis, S. , Snyder, A. Z. , McAvoy, M. P. , Sereno, M. I. , Corbetta, M. , & Shulman, G. L. (2015). Eye position modulates retinotopic responses in early visual areas: A bias for the straight‐ahead direction. Brain Structure and Function, 220(5), 2587–2601. 10.1007/s00429-014-0808-7 24942135 PMC4549389

[hbm24862-bib-0155] Sulpizio, V. , Committeri, G. , Lambrey, S. , Berthoz, A. , & Galati, G. (2013). Selective role of lingual/parahippocampal gyrus and retrosplenial complex in spatial memory across viewpoint changes relative to the environmental reference frame. Behavioural Brain Research, 242, 62–75. 10.1016/j.bbr.2012.12.031 23274842

[hbm24862-bib-0156] Sulpizio, V. , Committeri, G. , & Galati, G. (2014). Distributed cognitive maps reflecting real distances between places and views in the human brain. Frontiers in Human Neuroscience, 8(716), 1–16. 10.3389/fnhum.2014.00716 25309392 PMC4160952

[hbm24862-bib-0157] Sulpizio, V. , Boccia, M. , Guariglia, C. , & Galati, G. (2018). Neural codes for one's own position and direction in a real‐world "vista" environment. Frontiers in Human Neuroscience, 12, 167 10.3389/fnhum.2018.00167 29760655 PMC5936771

[hbm24862-bib-0158] Sunaert, S. , Van Hecke, P. , Marchal, G. , & Orban, G. A. (1999). Motion‐responsive regions of the human brain. Experimental Brain Research, 127, 355–370. 10.1007/s002210050804 10480271

[hbm24862-bib-0159] Swisher, J. D. , Halko, M. A. , Merabet, L. B. , McMains, S. A. , & Somers, D. C. (2007). Visual topography of human intraparietal sulcus. The Journal of Neuroscience, 27, 5326–5337. 10.1523/JNEUROSCI.0991-07.2007 17507555 PMC6672354

[hbm24862-bib-0160] Talairach, J. , & Tournoux, P. (1988). Co‐planar stereotaxic atlas of the human brain. New York, NY: Thieme.

[hbm24862-bib-0161] Tanaka, K. , Fukada, Y. , & Saito, H. (1989). Underlying mechanisms of the response specificity of expansion/contraction and rotation cells in the dorsal part of the MST area of the macaque monkey. Journal of Neurophysiology, 62, 642–656. 10.1152/jn.1989.62.3.642 2769352

[hbm24862-bib-0162] Tanaka, K. , & Saito, H. (1989). Analysis of motion of the visual field by direction, expansion/contraction, and rotation cells clustered in the dorsal part of the medial superior temporal area of the macaque monkey. Journal of Neurophysiology, 62(3), 626–641. 10.1152/jn.1989.62.3.626 2769351

[hbm24862-bib-0163] Tokumaru, O. , Kaida, K. , Ashida, H. , Yoneda, I. , & Tatsuno, J. (1999). EEG topographical analysis of spatial disorientation. Aviation, Space, and Environmental Medicine, 70, 256–263. 10102738

[hbm24862-bib-0164] Tootell, R. B. , Hadjikhani, N. , Hall, E. K. , Marrett, S. , Vanduffel, W. , Vaughan, J. T. , & Dale, A. M. (1998). The retinotopy of visual spatial attention. Neuron, 21, 1409–1422. 10.1016/S0896-6273(00)80659-5 9883733

[hbm24862-bib-0165] Tootell, R. B. , Reppas, J. B. , Kwong, K. K. , Malach, R. , Born, R. T. , Brady, T. J. , … Belliveau, J. W. (1995). Functional analysis of human MT and related visual cortical areas using magnetic resonance imaging. The Journal of Neuroscience, 15, 3215–3230. 10.1523/JNEUROSCI.15-04-03215.1995 7722658 PMC6577785

[hbm24862-bib-0166] Tootell, R. B. , Mendola, J. D. , Hadjikhani, N. K. , Ledden, P. J. , Liu, A. K. , Reppas, J. B. , & Dale, A. M. (1997). Functional analysis of V3A and related areas in human visual cortex. The Journal of Neuroscience: The Official Journal of the Society for Neuroscience, 17(18), 7060–7078. 10.1523/JNEUROSCI.17-18-07060.1997 9278542 PMC6573277

[hbm24862-bib-0167] Tosoni, A. , Galati, G. , Romani, G. L. , & Corbetta, M. (2008). Sensory‐motor mechanisms in human parietal cortex underlie arbitrary visual decisions. Nature Neuroscience, 11(12), 1446–1453. 10.1038/nn.2221 18997791 PMC3861399

[hbm24862-bib-0168] Tosoni, A. , Pitzalis, S. , Committeri, G. , Fattori, P. , Galletti, C. , & Galati, G. (2015). Resting‐state connectivity and functional specialization in human medial parieto‐occipital cortex. Brain Structure and Function, 220(6), 3307–3321. 10.1007/s00429-014-0858-x 25096286

[hbm24862-bib-0169] Tzourio‐Mazoyer, N. , Landeau, B. , Papathanassiou, D. , Crivello, F. , Etard, O. , Delcroix, N. , … Joliot, M. (2002). Automated anatomical labeling of activations in SPM using a macroscopic anatomical parcellation of the MNI MRI single‐subject brain. NeuroImage, 15(1), 273–289. 10.1006/nimg.2001.0978 11771995

[hbm24862-bib-0170] Uesaki, M. , & Ashida, H. (2015). Optic‐flow selective cortical sensory regions associated with self‐reported states of vection. Frontiers in Psychology, 6(775), 1–9. 10.3389/fpsyg.2015.00775 26106350 PMC4459088

[hbm24862-bib-0171] Vaina, L. M. , Gryzwacz, N. M. , Saiviroonporn, P. , LeMay, M. , Bienfang, D. C. , & Cowey, A. (2003). Can spatial and temporal motion integration compensate for deficits in local motion mechanisms? Neuropsychologia, 41(13), 1817–1836. 10.1016/S0028-3932(03)00183-0 14527545

[hbm24862-bib-0172] Van Essen, D. C. (2005). A population‐average, landmark‐ and surface‐based (PALS) atlas of human cerebral cortex. NeuroImage, 28(3), 635–662. 10.1016/j.neuroimage.2005.06.058 16172003

[hbm24862-bib-0173] Van Essen, D. C. , Glasser, M. F. , Dierker, D. L. , Harwell, J. , & Coalson, T. (2011). Parcellations and hemispheric asymmetries of human cerebral cortex analyzed on surface‐based atlases. Cerebral Cortex, 22(10), 2241–2262. 10.1093/cercor/bhr291 22047963 PMC3432236

[hbm24862-bib-0174] Van Oostende, S. , Sunaert, S. , Van Hecke, P. , Marchal, G. , & Orban, G. A. (1997). The kinetic occipital (KO) region in man: An fMRI study. Cerebral Cortex, 7(7), 690–701. 10.1093/cercor/7.7.690 9373023

[hbm24862-bib-0175] von Pföstl, V. , Stenbacka, L. , Vanni, S. , Parkkonen, L. , Galletti, C. , & Fattori, P. (2009). Motion sensitivity of human V6: A magnetoencephalography study. NeuroImage, 45(4), 1253–1263. 10.1016/j.neuroimage.2008.12.058 19211036

[hbm24862-bib-0176] Wada, A. , Sakano, Y. , & Ando, H. (2016). Differential responses to a visual self‐motion signal in human medial cortical regions revealed by wide‐view stimulation. Frontiers in Psychology, 7, 1–17. 10.3389/fpsyg.2016.00309 26973588 PMC4777731

[hbm24862-bib-0177] Wall, M. B. , Lingnau, A. , Ashida, H. , & Smith, A. T. (2008). Selective visual responses to expansion and rotation in the human MT complex revealed by functional magnetic resonance imaging adaptation. The European Journal of Neuroscience, 27, 2747–2757. 10.1111/j.1460-9568.2008.06249.x 18547254

[hbm24862-bib-0178] Wall, M. B. , & Smith, A. T. (2008). The representation of egomotion in the human brain. Current Biology, 18(3), 191–194. 10.1016/j.cub.2007.12.053 18221876

[hbm24862-bib-0179] Wandell, B. A. , & Winawer, J. (2011). Imaging retinotopic maps in the human brain. Vision Research, 51(7), 718–737. 10.1016/j.visres.2010.08.004 20692278 PMC3030662

[hbm24862-bib-0180] Wann, J. P. , Swapp, D. , & Rushton, S. K. (2000). Heading perception and the allocation of attention. Vision Research, 40, 2533–2543. 10.1016/S0042-6989(00)00115-2 10915891

[hbm24862-bib-0181] Wardak, C. , Hamed, S. B. , Olivier, E. , & Duhamel, J.‐R. (2012). Differential effects of parietal and frontal inactivations on reaction times distributions in a visual search task. Frontiers in Integrative Neuroscience, 29(6), 39 10.3389/fnint.2012.00039 PMC3386550 22754512

[hbm24862-bib-0182] Warren, P. A. , & Rushton, S. K. (2009a). Optic flow processing for the assessment of object movement during ego movement. Current Biology, 19(18), 1555–1560. 10.1016/j.cub.2009.07.057 19699091

[hbm24862-bib-0183] Warren, P. A. , & Rushton, S. K. (2009b). Perception of scene‐relative object movement: Optic flow parsing and the contribution of monocular depth cues. Vision Research, 49(11), 1406–1419. 10.1016/j.visres.2009.01.016 19480063

[hbm24862-bib-0184] Watson, J. D. , Myers, R. , Frackowiak, R. S. , Hajnal, J. V. , Woods, R. P. , Mazziotta, J. C. , … Zeki, S. (1993). Area V5 of the human brain: Evidence from a combined study using positron emission tomography and magnetic resonance imaging. Cerebral Cortex, 3, 79–94. 10.1093/cercor/3.2.79 8490322

