# Supplementary material for: Neural bases of self‐ and object‐motion in a naturalistic vision
Source: Hum Brain Mapp. 2019 Nov 11;41(4):1084–111. doi: 10.1002/hbm.24862 (PMC7267932; doi:10.1002/hbm.24862)
Supplement: Supplementary file 1 — Appendix S1: Supporting information [file HBM-41-1084-s002.docx]

Supplementary Material

**Neural bases of self- and object motion in a naturalistic vision**

Sabrina Pitzalis, Chiara Serra, Valentina Sulpizio, Giorgia Committeri, Francesco de Pasquale, Patrizia Fattori, Claudio Galletti, Rosamaria Sepe, Gaspare Galati

**Supplementary Experimental Procedures**

## **Localizer scans.**

In a second set of fMRI experiments, several localizer scans were conducted to define V6+, MT/MST+ and PPA/RSC regions of interest (ROIs) following standard procedures described in detail below.

*Localizers for V6+ ROI*. Two functional scans were acquired in separate sessions to define the ‘medial motion sensitive area V6’ as described in detail in Pitzalis et al. (2010) and as currently used in our laboratory (Pitzalis et al., 2012, 2013a,b,c, 2015; Tosoni et al., 2015; Serra et al., 2019). Briefly, while fixating a central cross, subjects viewed 16 s blocks of structured moving random dot fields (dilations, contractions, spirals and rotations) versus 16 s blocks of scrambled moving dot controls (Fig. 3A). A new field of white dots was generated every 500 ms (total number of dots= 2000; dot size 0.4 x 0.4°). The average luminance of the stimulus was 31 cd/m^2^. Flowfields stimuli were presented using a full-field stimulation and a wide field set-up (70 x 55 deg, see Apparatus). In a previous paper (Pitzalis et al., 2010) we showed that area V6 is selectively driven by flowfields even with a standard size stimulus (23 x 12 deg). Both the wide field and regular versions of the optic flow stimulus induced vection according to subject verbal reports after the session. However, induced vection was particularly compelling with wide-field stimuli. Originally, we found that the flowfields stimulus could be used as a V6 localizer, because it was able to map a region in the Parieto-Occipital sulcus (POs) overlapping with the retinotopically defined human area V6 (Pitzalis et al., 2006). Later, we found that also the neighboring retinotopic area V6Av responds to flowfields (Pitzalis et al., 2013d, 2015; Tosoni et al., 2015), although to a lesser extent than V6. Hence, given that V6 as defined with the flowfields localizer may also marginally include the more anterior V6Av, here we refer to this region as V6 complex (or V6+).

*Localizers for MT and MST+ ROIs*. In a subgroup of eight subjects, MST was defined (and distinguished from MT) using the criterion originally introduced by Dukelow et al. (2001) and later used also in other laboratories (Huk et al., 2002; Smith et al., 2006; Wall et al., 2008; Wall and Smith, 2008; Cardin and Smith, 2011; Cardin et al., 2012; Furlan et al., 2014; Smith et al., 2012; Pitzalis et al., 2013c; Frank et al., 2014). Stimuli consisted in 16 s blocks of high contrast (100%) moving dots alternated with 16 s blocks of stationary dots (Fig. 3B). Dots moved (15 deg/s) alternately inwards and outwards along the radial axes (thus creating alternating contraction and expansion). Inwards and outwards motion were alternated every 2 seconds. The appearance of new dots was controlled to maintain a constant dot density (0.04 dots/degrees^2^ on average). The dots (0.4 x 0.4 deg) were restricted to a peripheral circular aperture (15 deg diameter) presented with its center placed 10 deg to the left or right of fixation. Stimuli were therefore restricted to either the left or right hemifield (one scanning run was completed for each hemifield). These peripheral moving stimuli would be expected to evoke neuronal activity in the contralateral hemisphere in both MT and MST, but in the ipsilateral hemisphere only in MST, where the receptive fields are large enough to extend into the ipsilateral hemifield (e.g., Dukelow et al., 2001; Huk et al., 2002). Hence, with this procedure, MT and MST can be differentiated in terms of the absence or presence of ipsilateral responses, respectively. However, a limit of the MST definition introduced by Huk et al (2002) is that at least four nearby monkey areas (MSTv, MSTd, FST and LST) have the properties used to define MST (ipsilateral representation and motion sensitivity; (e.g., Nelissen et al., 2006; Kolster et al., 2010). Thus, what is labeled here MST is likely a mosaic of cortical areas and for these reasons hereafter we will call this region MST complex (or MST+).

*Localizers for PPA and RSC ROIs*. Two functional scans were acquired to define the parahippocampal place area (PPA) and the retrosplenial complex (RSC) as in previous studies (see Epstein, 2008 for a review) and as currently used in our laboratory (e.g., Sulpizio et al., 2013, 2014; Tosoni et al., 2008). Stimuli consisted of pictures of faces and pictures of places/scenes (Fig. 3C). Photos of places consisted of common indoor (50%) and outdoor (50%) scenes. Photos of face represented faces with neutral expressions of male (50%) and female (50%) young adults. Faces were set on a solid gray background. White fixation crosses on a black background were shown between successive stimuli. Stimuli were presented for 300 ms and every 500 ms in 16 s blocks and alternated with 15 s fixation block.

*Statistical analyses of fMRI data and ROI definition*

*Train experiment*. Beyond the twelve regions defined from the motion vs. static (M-S) contrast, we verified the presence of motion-related responses also in the very recently defined visual cortical area prostriata (Mikellidou et al., 2017). This region has a retinotopic representation of the contralateral visual field, and, more importantly for the present paper, it has a preference for peripheral fast motion. To explore this ventral activity, we used an omnibus F-contrast comparing any motion condition against fixation at a more liberal statistical threshold (p < 0.01 unc; extent threshold = 20 voxels) to define a spherical ROI of 8 mm of radius in the left and right hemispheres centered on MNI coordinates (see caption of Supplementary Figure 2) in line with those provided by Glasser et al. (2016) and Mikellidou et al. (2017).

*Localizer scans.* For the localizer scans, stimulation blocks were modelled as box-car functions spanning the whole block duration and convolved with a standard hemodynamic response function. We used individually adjusted uncorrected thresholds to define these regions (p < 0.001 or higher). Then, we followed a specific procedure to define each ROI.

To define the V6+ ROI, we used the same procedure described in our previous paper (Pitzalis et al., 2010). V6+ was defined as all contiguous voxels that were more active during coherent optic flow than random motion in the medial POs. Specifically, V6+ was defined as a region located in or near the dorsalmost portion of the posterior and anterior banks of the POs, medially to V3A. This position is consistent with the original retinotopic definition of human V6 of Pitzalis et al. (2006) although it extends anteriorly to marginally include also the neighboring V6Av (Pitzalis et al., 2013d; Tosoni et al., 2015). V6+ was successfully defined in 28/28 hemispheres.

To define the MT/MST+ ROI, we followed the same procedure described several times in previous papers (e.g., Wall and Smith, 2008; Smith et al., 2006; Dukelow et al., 2001; Huk et al., 2002; Wall et al., 2008; Pitzalis et al., 2013c) that can be summarized as follows. The entire MT+ is activated by contralateral stimuli, whereas ipsilateral stimuli drive only MST+. MST+ was defined as all contiguous voxels within MT+ that were significantly active during ipsilateral motion stimulation. MT was defined as all contiguous active voxels that were active during contralateral but not ipsilateral stimulation, with the proviso that any MT voxels situated further anterior than the median value of the MST+ ROI on the horizontal (axial) plane were removed from the MT ROI (e.g., Wall et al., 2008). MT was successfully defined in 16/16 hemispheres. Although ipsilateral responses were relatively weak compared with contralateral responses, a subregion of ipsilateral activity was clearly identifiable in 15/16 hemispheres and was marked as MST+.

To define the PPA and RSC ROIs we followed the criterion described in Epstein (2008) and used in previous papers from our laboratory (e.g., Sulpizio et al., 2013, 2014). Shortly, the PPA and the RSC were identified in individual subjects as the regions responding more strongly to places/scenes than to faces in the posterior parahippocampal cortex and in the retrosplenial cortex, respectively. Specifically, PPA was defined as a region at the boundary between posterior parahippocampal cortex and the anterior lingual gyrus. According to Epstein (2008), the RSC was defined as a region including the posterior cingulate (Brodmann areas 23–31), the retrosplenial cortex proper (Brodmann areas 29–30), and the nearby ventral parietal-occipital sulcus and anterior calcarine sulcus. Both PPA and RSC were successfully defined in 26/28 hemispheres.

Finally, the regional hemodynamic responses from the regions defined by functional localizers (V6+, MT/MST+, PPA/RSC) and from the prostriate, were analysed separately for each region by means of one-way ANOVAs with condition (static, offboard, onboard, joint and disjoint) as factor. In this ANOVA, post-hoc comparisons were conducted using the Duncan’s New Multiple Range Test which applies the multiple comparison correction.

*Anatomical image processing*

Structural images were analyzed using FreeSurfer 5.1 (<http://surfer.nmr.mgh.harvard.edu/>) to obtain a surface representation of each individual cortical hemisphere in a standard space. We used the “recon-all” fully automated processing pipeline, which performs intensity correction, transformation to Talairach space, normalization, skull-stripping, subcortical and white-matter segmentation, surface tessellation, surface refinement, surface inflation, sulcus-based nonlinear morphing to a cross-subject spherical coordinate system, and cortical parcellation (Dale et al., 1999; Fischl et al., 1999; Desikan et al., 2006).

**Supplementary** **Results**

We studied the functional response profile of seventeen regions [V6 (V6+), PEc, pCi, CSv, CMA, PIC, LOR, MT+ (MT and MST+), V3A/V7, IPSmot, LIP, SFS, PPA and RSC] to explore their sensitivity to different types of motion conditions and to the amount of retinal and perceived motion. The mean percentage signal changes we observed in the motion and static conditions relative to the fixation baseline are plotted in the column histograms of Figures 5, 6, 7, 8 and 9. Statistical results of this analysis are also detailed in the supplementary Table 1.

In absence of a retinotopic mapping or functional localizers, we defined some regions (V3A/V7, LOR and LIP) based on their anatomical positions, MNI coordinates, neighboring relations with the other regions and position respect to the sulcal pattern. In addition, to illustrate the relationship between our findings and the location of specific visual areas, we overlaid these three regions onto the Conte69 surface-based atlas (Van Essen et al. 2011) along with the borders of many known retinotopic areas, as done in a previous paper from our lab (Tosoni et al., 2015). The critical advancement of using the Conte69 atlas is that we could put our three regions directly in relation to parcellations of human cerebral cortex already ported to this atlas, such as those of early visual areas and of the lateral occipito-temporal MT complex (MT+) and of the intraparietal sulcus based on retinotopic data (Kolster et al. 2010; Schluppeck et al. 2005; Silver et al. 2005; Swisher et al. 2007). As shown in Supplementary Figure 1, this overlay shows a good consistency between our regions and the atlas, especially for LOR and LIP. As V3A, the consistency is less striking in that it seems to overlap mainly the retinotopic dorsal area V7 (Tootell et al., 1998) encompassing also area V3A (Tootell et al., 1997), especially in the left hemisphere (Sereno et al., 1995) but not V3B (Smith et al., 1998). Note, however, that because these parcellations are typically based on retinotopic or functional data from single subjects or small groups of subjects, descriptions of the overlay between our group regions and areal borders from these parcellations are only descriptive and should be considered with caution. For these reasons, we conservatively labelled the region V3A/V7.

Finally, we studied the functional response profile of the prostriate region defined as described above, to explore its sensitivity to different types of motion conditions. Supplementary Figure 2A shows the position of the prostriate region on the template brain. This region is located along the medial wall, at the junction between the calcarine cortex and the very ventral end of the POs (Mikellidou et al., 2017).

The one-way repeated-measures ANOVA with motion condition as factor revealed a significant main effect of motion condition (F_(4,108)_= 5.33, p < .001). As shown in the plots of Supplementary Figure 2B, post hoc tests indicated that the prostriate responds more to Disjoint than Onboard (p=0.007), thus preferring complex combinations of self & object motion. It is not involved in the flow parsing phenomenon, being unable to distinguish Onboard from Joint (p= 0.19, n.s.)

**Supplementary Figure legends**

**Figure 1.** Three regions (LOR, LIP, V3A/V7) are superimposed over the flattened left and right hemispheres of Conte69 atlas (Van Essen et al. 2011). The borders of previously identified areas (Van Essen et al. 2011; Kolster et al. 2010) are highlighted in white. The curvature is shown using light/dark gray to signify convex/concave. LH, Left Hemisphere. RH, right hemisphere. Sup. Temp., Superior Temporal sulcus.

**Figure 2.** Area prostriate. **A**. Prostriate mapping by comparing any motion condition against fixation. Results are displayed on the medial and lateral folded representation of the right and left hemispheres of the template brain. The MNI coordinates (mm) and sizes (mm^3^) of the prostriate region are as follows: LH, x= -24, y= -58, z=0, size=79; RH, x=24, y= -57, z=1, size= 81. **B**. The plot for the prostriate region represents the averaged BOLD percent signal change ± standard error of the mean across subjects and hemispheres for each experimental condition: Static (Black), Offboard (Red), Onboard (Blue), Joint (Yellow) and Disjoint (Green). Significant comparisons are also reported. *p<0.05; **p< 0.01; ***p< 0.001. Name abbreviations for some of the conditions are as follows: Sta (Static), Offb (Offboard), Onb (Onboard), Disj (Disjoint). Significant comparisons are also reported. *p<0.05; **p< 0.01; ***p< 0.001.

**Supplementary References**

Cardin V, Hemsworth L, Smith AT (2012): Adaptation to heading direction dissociates the roles of human MST and V6 in the processing of optic flow. J Neurophysiol 108(3): 794-801. <https://doi.org/10.1152/jn.00002.2012>

Cardin V, Smith AT (2011): Sensitivity of human visual cortical area V6 to stereoscopic depth gradients associated with self-motion. J Neurophysiol 106 (3): 1240-9. <https://doi.org/10.1152/jn.01120.2010>

Dale AM, Fischl B, Sereno MI (1999): Cortical surface-based analysis I Segmentation and surface reconstruction. Neuroimage 9: 179-194. [https://doi.org/10.1006/nimg.1998.0395](https://doi.org/10.1006/nimg.1998.0395)

Desikan, R.S., Ségonne, F., Fischl, B., Quinn, B.T., Dickerson, B.C., Blacker, D., Buckner, R.L., Dale, A.M., Maguire, R.P., Hyman, B.T. (2006). An automated labeling system for subdividing the human cerebral cortex on MRI scans into gyral based regions of interest. *Neuroimage 31*, 968-980. [https://doi.org/10.1016/j.neuroimage.2006.01.021](https://doi.org/10.1016/j.neuroimage.2006.01.021)

Dukelow SP, DeSouza JF, Culham JC, van den Berg AV, Menon RS, Vilis T (2001): Distinguishing subregions of the human MT+ complex using visual fields and pursuit eye movements. J Neurophysiol 86(4): 1991-2000. <https://doi.org/10.1152/jn.2001.86.4.1991>

Epstein RA (2008): Parahippocampal and retrosplenial contributions to human spatial navigation. Trends Cognit Sci 12: 388–396. <https://doi.org/10.1016/j.tics.2008.07.004>

Fischl B, Sereno MI, Dale AM (1999): Cortical surface-based analysis: II: inflation, flattening, and a surface-based coordinate system. Neuroimage, 9: 195-207. [https://doi.org/10.1006/nimg.1998.0396](https://doi.org/10.1006/nimg.1998.0396)

Frank SM, Baumann O, Mattingley JB, Greenlee MW (2014): [Vestibular and visual responses in human posterior insular cortex](https://www.ncbi.nlm.nih.gov/pubmed/25185806). J Neurophysiol 112(10): 2481-91. <https://doi.org/10.1152/jn.00078.2014>

Furlan M, Wann JP, Smith AT (2014): A representation of changing heading direction in human cortical areas pVIP and CSv. Cereb Cortex 24(11): 2848-58. <https://doi.org/10.1093/cercor/bht132>

Glasser, M.F., Coalson, T.S., Robinson, E.C., Hacker, C.D., Harwell, J., Yacoub, E., Ugurbil, K., Andersson, J., Beckmann, C.F., Jenkinson, M., et al. (2016). A multi-modal parcellation of human cerebral cortex. Nature 536, 171–178.  doi: 10.1038/nature18933.

Huk AC, Dougherty RF, Heeger DJ (2002): Retinotopy and functional subdivision of human areas MT and MST. J Neurosci 22: 7195–7205. https://doi.org/10.1523/JNEUROSCI.22-16-07195.2002

Kolster H, Peeters R, Orban GA (2010): The retinotopic organization of the human middle temporal area MT/V5 and its cortical neighbors. J Neurosci, 30(29): 9801-20.  https://doi.org/10.1523/JNEUROSCI.2069-10.2010

Mikellidou, K., Kurzawski, J. W., Frijia, F., Montanaro, D., Greco, V., Burr, D. C., & Morrone, M. C. (2017). Area prostriata in the human brain. Current Biology, 27(19), 3056–3060.e3. http://doi.org/10.1016/j.cub.2017.08.065

Nelissen K, Vanduffel W, Orban GA (2006): Charting the lower superior temporal region, a new motion-sensitive region in monkey superior temporal sulcus. J Neurosci 26(22): 5929-47. https://doi.org/10.1523/JNEUROSCI.0824-06.2006

Pitzalis S, Bozzacchi C, Bultrini A, Fattori P, Galletti C, Di Russo F (2013a): Parallel motion signals to the medial and lateral motion areas V6 and MT+. Neuroimage 67: 89-100. [https://doi.org/10.1016/j.neuroimage.2012.11.022](https://doi.org/10.1016/j.neuroimage.2012.11.022)

Pitzalis S, Fattori P, Galletti C (2013b): The functional role of the medial motion area V6. Front Behav Neurosci 6: 91. <https://doi.org/10.3389/fnbeh.2012.00091>

Pitzalis S, Fattori P, Galletti C (2015): The human cortical areas V6 and V6A. Vis Neurosci 32: E007.  <https://doi.org/10.1017/S0952523815000048>

Pitzalis S, Galletti C, Huang RS, Patria F, Committeri G, Galati G, Fattori P, Sereno MI (2006): Wide-field retinotopy defines human cortical visual area V6. J Neurosci 26: 7962-7973. https://doi.org/10.1523/JNEUROSCI.0178-06.2006

Pitzalis S, Sdoia S, Bultrini A, Committeri G, Di Russo F, Fattori P, Galletti C, Galati G (2013c): Selectivity to translational egomotion in human brain motion areas. PloS One, 8(4): e60241. <http://doi.org/10.1371/journal.pone.0060241>

Pitzalis S, Sereno MI, Committeri G, Fattori P, Galati G, Patria F, Galletti C (2010): Human V6: the medial motion area. Cerebral Cortex 20(2): 411-24. <http://doi.org/10.1093/cercor/bhp112>

Pitzalis S, Sereno MI, Committeri G, Fattori P, Galati G, Tosoni A, Galletti C (2013d): The human homologue of macaque area V6A. Neuroimage 82: 517–530. <http://doi.org/10.1016/j.neuroimage.2013.06.026>

Pitzalis S, Strappini F, De Gasperis M, Bultrini A, Di Russo F (2012): Spatio-Temporal Brain Mapping of Motion-Onset VEPs combined with fMRI and Retinotopic Maps. Plos One 7(4): e3577. <http://doi.org/10.1371/journal.pone.0035771>

Schluppeck D, Glimcher P, Heeger DJ (2005): Topographic organization for delayed saccades in human posterior parietal cortex. J Neurophysiol 94(2): 1372-84. <https://doi.org/10.1152/jn.01290.2004>

Sereno MI, Dale AM, Reppas JB, Kwong KK, Belliveau JW, Brady TJ, Rosen BR, Tootell RBH (1995): Borders of multiple visual areas in humans revealed by functional magnetic resonance imaging. Science 268: 889–893. https://doi.org/10.1126/science.775437

Serra C, Galletti C, Di Marco S, Fattori P, Galati G, Sulpizio V, Pitzalis S. (2019). Egomotion-related visual areas respond to active leg movements. Hum Brain Mapp. Mar 28. https://doi.org/10.1002/hbm.24589

Silver MA, Ress D, Heeger DJ (2005): Topographic maps of visual spatial attention in human parietal cortex. J Neurophysiol 94(2): 1358–1371. <https://doi.org/10.1152/jn.01316.2004>

Smith AT, Greenlee MW, Singh KD, Kraemer FM, Hennig J (1998): The processing of first- and second-order motion in human visual cortex assessed by functional magnetic resonance imaging (fMRI). J Neurosci 18: 3816-3830. https://doi.org/10.1523/JNEUROSCI.18-10-03816.1998

Smith AT, Wall MB, Thilo KV (2012): Vestibular inputs to human motion-sensitive visual cortex. Cereb Cortex 22(5): 1068-77. <http://doi.org/10.1093/cercor/bhr179>

Smith T, Wall MB, Williams AL, Singh KD (2006): Sensitivity to optic flow in human cortical areas MT and MST. Eur J Neurosci 23: 561–569. <http://doi.org/10.1111/j.1460-9568.2005.04526.x>

Sulpizio V, Committeri G, Galati G (2014): Distributed cognitive maps reflecting real distances between places and views in the human brain. Front Hum Neurosci 8: 716. <http://doi.org/10.3389/fnhum.2014.00716>

Sulpizio V, Committeri G, Lambrey S, Berthoz A, Galati G (2013): Selective role of lingual/parahippocampal gyrus and retrosplenial complex in spatial memory across viewpoint changes relative to the environmental reference frame. Behav Brain Res 242: 62-75. [http://doi.org/10.1016/j.bbr.2012. 12.031](http://doi.org/10.1016/j.bbr.2012.%2012.031)

Swisher JD, Halko MA, Merabet LB, McMains SA, Somers DC (2007): Visual topography of human intraparietal sulcus. J Neurosci, 27: 5326–5337. https://doi.org/10.1523/JNEUROSCI.0991-07.2007

Tootell RB, Hadjikhani N, Hall EK, Marrett S, Vanduffel W, Vaughan JT, Dale AM (1998): The retinotopy of visual spatial attention. Neuron 21: 1409 –1422. <https://doi.org/10.1016/S0896-6273(00)80659-5>

Tootell RBH, Mendola JD, Hadjikhani NK, Ledden PJ, Liu AK, Reppas JB, Sereno MI, Dale AM (1997): Functional analysis of V3A and related areas in human visual cortex. J Neurosci 17: 7076-7078. <https://doi.org/10.1523/JNEUROSCI.17-18-07060.1997>

Tosoni A, Galati G, Romani GL, Corbetta M (2008): Sensory-motor mechanisms in human parietal cortex underlie arbitrary visual decisions. Nat Neurosci 11(12): 1446–1453. <http://doi.org/10.1038/nn.2221>

Tosoni A, Pitzalis S, Committeri G, Fattori P, Galletti C, Galati G (2015): Resting-state Connectivity and Functional Specialization in Human Medial Parietooccipital Cortex. Brain Struct Funct 220(6): 3307-21. <http://doi.org/10.1007/s00429-014-0858-x>

Van Essen DC, Glasser MF, Dierker DL, Harwell J, Coalson T (2011): Parcellations and hemispheric asymmetries of human cerebral cortex analyzed on surface-based atlases. Cereb Cortex 22(10): 2241–2262. <http://doi.org/10.1093/cercor/bhr291>

Wall MB, Lingnau A, Ashida H Smith AT (2008): Selective visual responses to expansion and rotation in the human MT complex revealed by functional magnetic resonance imaging adaptation. Eur J Neurosci 27: 2747-2757. https://doi.org/10.1111/j.1460-9568.2008.06249.x

Wall MB, Smith AT (2008): The Representation of Egomotion in the Human Brain. Curr Biol 18: 191-194. <http://doi.org/10.1016/j.cub.2007.12.053>

| **Region** | **Hemisphere** | **Condition** | **Post-hoc comparisons** |
| --- | --- | --- | --- |
| **PEc** | 14/28 Hs | F _(3,39)_= 3.06* | ONB>OFFB*; ONB= DISJ, p=0.3, n.s.; JOINT=ONB, p=0.7, n.s.; |
| **pCi** | 28/28 H | F _(3,81)_= 6.61*** | ONB>OFFB*; ONB= DISJ, p=0.07, n.s.; JOINT=ONB, p=0.07, n.s.; |
| **CSv** | 28/28 Hs | F _(3,81)_= 28.18*** | ONB>OFFB***; ONB= DISJ, p=0.33, n.s.; JOINT=ONB, p=0.7, n.s.; |
| **CMA** | 14/28 Hs | F _(3,39)_= 10.16*** | ONB>OFFB**; DISJ =ONB, p=0.11, n.s.; JOINT> ONB**; |
| **PIC** | 14/28Hs | F _(3,39)_= 4.028* | ONB>OFFB*; ONB= DISJ, p=0.38, n.s.; JOINT=ONB, p=0.5, n.s.; |
| **LOR** | 28/28 Hs | F _(3,81)_= 36.06*** | ONB>OFFB***; JOINT>DISJ***; JOINT> ONB**; |
| **MT+** | 28/28 Hs | F _(3,81)_= 13.62*** | OFFB> ONB**; DISJ> OFFB**; JOINT> ONB***; |
| **V6** | 28/28 Hs | F _(3,81)_= 7.77*** | ONB= OFFB, p=0.82, n.s.; DISJ>JOINT*, ONB***, OFFB***; JOINT=ONB, p=0.08, n.s.; |
| **V3A/V7** | 28/28 Hs | F _(3,81)_= 9.53*** | ONB= OFFB, p=0.14, n.s.; DISJ> OFFB***, ONB***; JOINT>ONB**; |
| **IPSmot** | 28/28 Hs | F _(3,81)_= 6.59*** | ONB= OFFB, p=0.51, n.s.; DISJ> ONB***, OFFB*; JOINT>ONB**; |
| **LIP** | 28/28 Hs | F _(3,81)_= 4.85** | ONB= OFFB, p=0.42, n.s.; DISJ> OFFB**, ONB***; |
| **SFS** | 28/28 Hs | F _(3,81)_= 13.61*** | ONB>OFFB***; DISJ> ONB**; JOINT>ONB*; |

**Supplementary Table 1**. Statistical results of the one-way ANOVA with condition (Offboard, Onboard, Joint, Disjoint) as factor conducted in each brain region defined by the motion vs. static contrast. *p<0,05; **p< 0.001, ***p>0.0001. Experimental conditions are labelled as follows: ONB, Onboard; OFFB, Offboard; JOINT; DISJ, Disjoint. Hs, hemispheres.

| **Region** | **Hemisphere** | **Condition** | **Post-hoc comparisons** |
| --- | --- | --- | --- |
| **MT** | 16/16 Hs | F _(4,60)_= 6.23*** | OFFB> ONB*; OFFB= DISJ, p=0.3, n.s.; JOINT> ONB**; |
| **MST** | 15/16 Hs | F _(4,56)_= 3.77** | ONB= OFFB, p=0.47, n.s.; DISJ> ONB*; JOINT=ONB, p=0.08, n.s.; |
| **V6+** | 28/28 Hs | F _(4,108)_= 21.08*** | ONB= OFFB, p=0.09, n.s.; DISJ> OFFB***, ONB***, JOINT*; Any condition> STA*; JOINT>ONB*; |
| **RSC** | 26/28 Hs | F _(4,100)_= 1.38, n.s. | n.s. |
| **PPA** | 26/28 Hs | F _(4,100)_= 11.63*** | ONB= OFFB, p=0.81, n.s.; DISJ> OFFB**, ONB**; JOINT=ONB, p=0.08, n.s.; |

**Supplementary Table 2**. Statistical results of the one-way ANOVA with condition (Offboard, Onboard, Joint, Disjoint) as factor conducted in each brain region defined by the three functional localizers (MT, MST+, V6+, RSC and PPA). *p<0,05; **p< 0.001, ***p>0.0001. Experimental conditions are labelled as follows: ONB, Onboard; OFFB, Offboard; JOINT; DISJ, Disjoint. Hs, hemispheres.
