# Supplementary figures and images for: Neural bases of self‐ and object‐motion in a naturalistic vision
Source: Hum Brain Mapp. 2019 Nov 11;41(4):1084–111. doi: 10.1002/hbm.24862 (PMC7267932; doi:10.1002/hbm.24862)

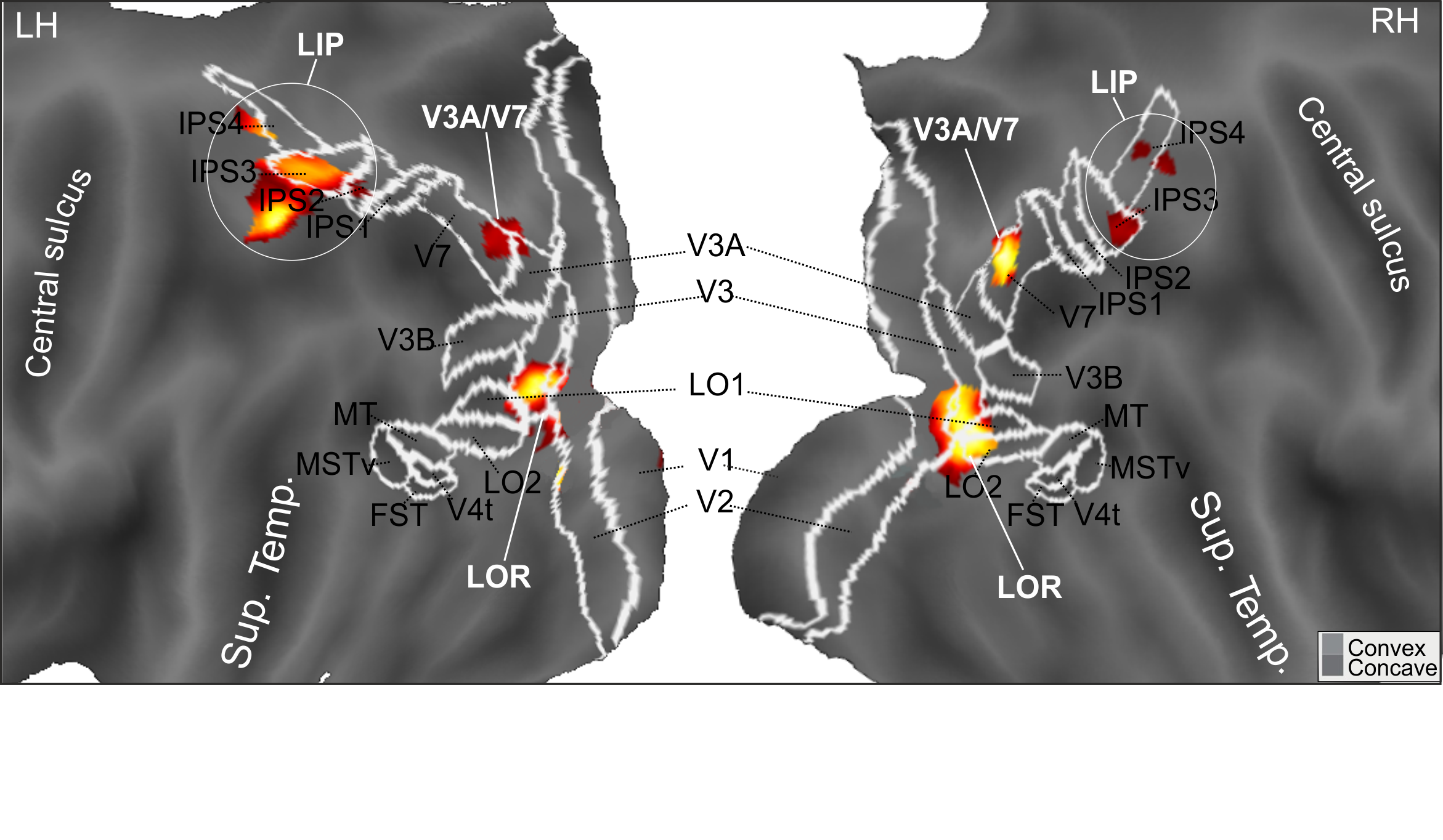

Supplement: Supplementary file 6 — Figure S1 Three regions (LOR, LIP, V3A/V7) are superimposed over the flattened left and right hemispheres of Conte69 atlas (Van Essen et al. 2011). The borders of previously identified areas (Van Essen et al. 2011; Kolster et al. 2010) are highlighted in white. The curvature is shown using light/dark gray to signify convex/concave. LH, Left Hemisphere. RH, right hemisphere. Sup. Temp., Superior Temporal sulcus. [file HBM-41-1084-s001.tif]

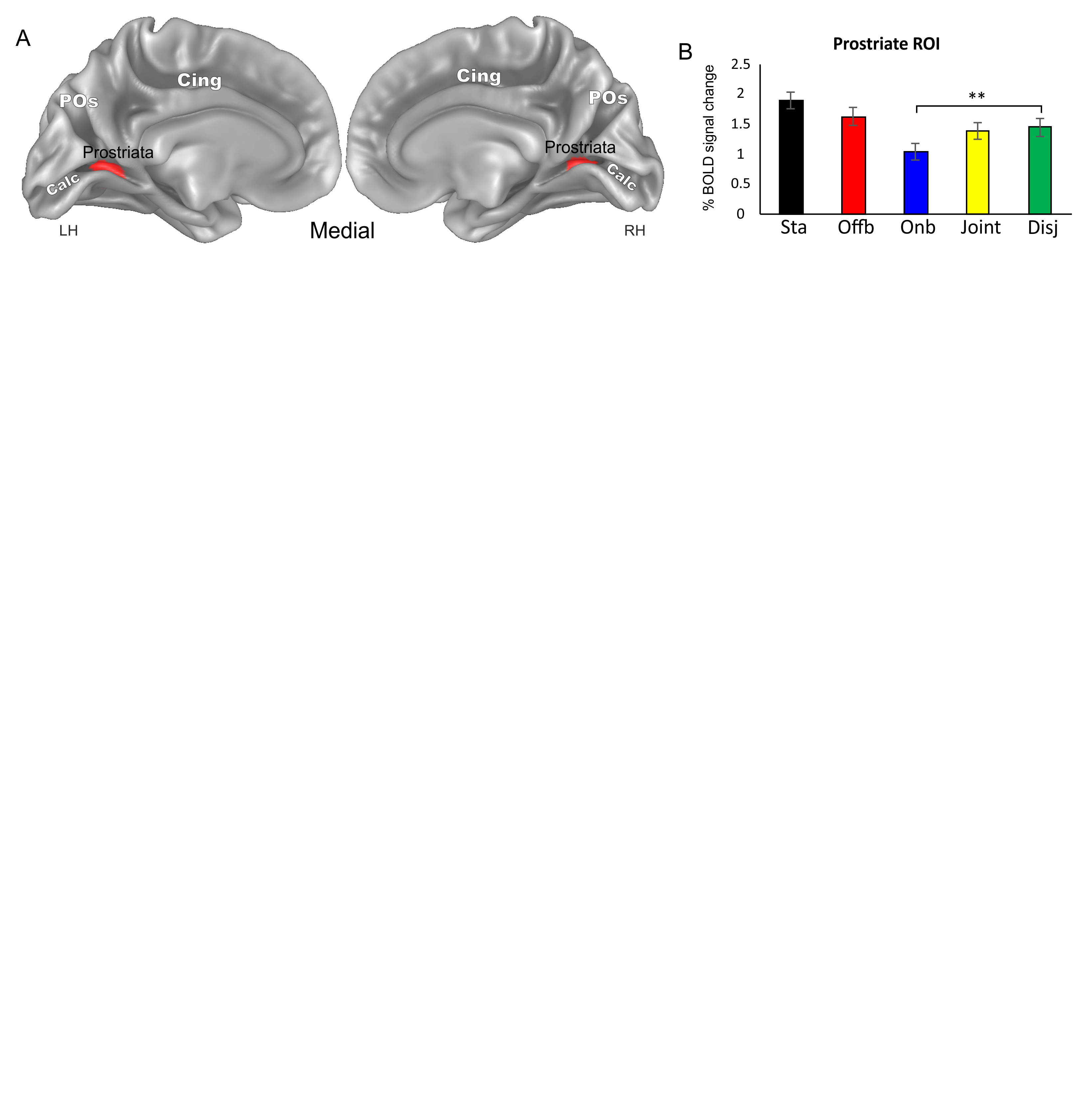

Supplement: Supplementary file 7 — Figure S2 Area prostriate. A. Prostriate mapping by comparing any motion condition against fixation. Results are displayed on the medial and lateral folded representation of the right and left hemispheres of the template brain. The MNI coordinates (mm) and sizes (mm3) of the prostriate region are as follows: LH, x = −24, y = −58, z = 0, size = 79; RH, x = 24, y = −57, z = 1, size = 81. B. The plot for the prostriate region represents the averaged BOLD percent signal change ± standard error of the mean across subjects and hemispheres for each experimental condition: Static (Black), Offboard (Red), Onboard (Blue), Joint (Yellow) and Disjoint (Green). Significant comparisons are also reported. *p < 0.05; **p < 0.01; ***p < 0.001. Name abbreviations for some of the conditions are as follows: Sta (Static), Offb (Offboard), Onb (Onboard), Disj (Disjoint). Significant comparisons are also reported. *p < 0.05; **p < 0.01; ***p < 0.001. [file HBM-41-1084-s004.tif]
